# Transcriptome profiling reveals that feeding wild zooplankton to larval Atlantic cod (*Gadus morhua*) influences suites of genes involved in oxidation-reduction, mitosis, and selenium homeostasis

**DOI:** 10.1186/s12864-015-2120-1

**Published:** 2015-11-26

**Authors:** Matthew L. Rise, Jennifer R. Hall, Gordon W. Nash, Xi Xue, Marije Booman, Tomer Katan, A. Kurt Gamperl

**Affiliations:** Department of Ocean Sciences, Memorial University of Newfoundland, St. John’s, NL A1C 5S7 Canada; Aquatic Research Cluster, CREAIT Network, Ocean Sciences Centre, Memorial University of Newfoundland, St. John’s, NL A1C 5S7 Canada; Present address: Pacific Biological Station, Fisheries and Oceans Canada, Nanaimo, BC V9T 6N7 Canada

**Keywords:** Atlantic cod, Diet, Gene expression, Growth, Larvae, Microarray, mRNA, Zooplankton

## Abstract

**Background:**

Larval nutrition and growth are key issues for wild and cultured cod. While it was shown previously that larval cod fed wild zooplankton grow faster than those fed only rotifers, the mechanisms involved in this enhanced growth are not completely understood. We used microarrays to identify larval cod transcripts that respond to feeding with small amounts of wild zooplankton (5–10 % of live prey items). The larval transcriptome was compared between 3 treatment groups [fed rotifers (RA), rotifers with protein hydrolysate (RA-PH), or rotifers with zooplankton (RA-Zoo)] at 9–10 mm length [26–30 days post-hatch (dph)] to identify a robust suite of zooplankton-responsive genes (i.e. differentially expressed between RA-Zoo and both other groups).

**Results:**

The microarray experiment identified 147 significantly up-regulated and 156 significantly down-regulated features in RA-Zoo compared with both RA and RA-PH. Gene ontology terms overrepresented in the RA-Zoo responsive gene set included “response to selenium ion” and several related to cell division and oxidation-reduction. Ten selenoprotein-encoding genes, and 2 genes involved in thyroid hormone generation, were up-regulated in RA-Zoo compared with both other groups. Hierarchical clustering of RA-Zoo responsive genes involved in oxidation-reduction and selenium homeostasis demonstrated that only the zooplankton treatment had a considerable and consistent impact on the expression of these genes. Fourteen microarray-identified genes were selected for QPCR involving 9–13 mm larvae, and 13 of these were validated as differentially expressed between RA-Zoo and both other groups at ~9 mm. In contrast, in age-matched (34–35 dph; ~11 mm RA and RA-PH, ~13 mm RA-Zoo) and size-matched (~13 mm) older larvae, only 2 and 3 genes, respectively, showed the same direction of RA-Zoo-responsive change as in ~9 mm larvae.

**Conclusions:**

The modulation of genes involved in selenium binding, redox homeostasis, and thyroid hormone generation in ~9 mm RA-Zoo larvae in this study may be in response to the relatively high levels of selenium, iodine, and LC-PUFA (potentially causing oxidative stress) in zooplankton. Nonetheless, only a subset of zooplankton-responsive genes in ~9 mm larvae remained so in older larvae, suggesting that the observed transcriptome changes are largely involved in initiating the period of growth enhancement.

**Electronic supplementary material:**

The online version of this article (doi:10.1186/s12864-015-2120-1) contains supplementary material, which is available to authorized users.

## Background

The Atlantic cod (*Gadus morhua*) is an economically and ecologically important species. Due to unpredictable and variable harvests of wild cod, several countries including Canada, Norway, Iceland, the UK, Ireland, and the Faroe Islands (Denmark) invested in the development of Atlantic cod aquaculture to meet consumer demand for this species [1–4]. Larval diet/nutrition, growth, and survival are key issues for both wild and cultured Atlantic cod populations [5–7].

The main food for wild marine fish larvae is zooplankton (e.g. copepods), whereas intensively cultured marine fish larvae are often fed rotifers followed by *Artemia* [8]. Survival of wild cod larvae, and recruitment, are influenced by the quantity and quality of zooplankton among other factors, such as temperature [5, 9, 10]. In culture, larval Atlantic cod exclusively fed wild zooplankton (primarily copepod nauplii) for even a brief period (2 weeks) have been shown to grow faster and with fewer deformities than larval cod fed only enriched rotifers, and the growth benefits associated with feeding zooplankton to cod larvae extend into the juvenile stage [11–14]. It is thought that differences in the nutritional composition of zooplankton and rotifers may underlie the enhanced growth performance of zooplankton-fed cod larvae [14]. For example, zooplankton often have higher levels of the ω3 long-chain polyunsaturated fatty acids (LC-PUFA), eicosapentaenoic acid (EPA) and docosahexaenoic acid (DHA), as well as several trace elements (e.g. selenium, iodine, copper, and zinc), than rotifers [15, 16]. Furthermore, zooplankton-fed cod larvae are reported to have higher levels of EPA, DHA, iodine, manganese, and selenium than cod larvae fed rotifers [14, 17]. While nutritional quality likely plays a key role, the precise mechanisms by which consumption of zooplankton accelerates larval cod growth are not completely understood.

We used a functional genomics approach to identify genes and molecular pathways in larval cod that respond to feeding with small amounts of wild zooplankton (i.e. 5–10 % of live prey items). The feeding regime providing samples for this microarray-based research, as well as the impact of dietary zooplankton on larval and juvenile cod growth, are reported in Katan et al. [18]. At approximately 30 days post-hatch (30 dph), the lengths and weights of cod larvae fed rotifers supplemented with wild zooplankton (RA-Zoo) from 2 dph began to separate from those fed either rotifers alone (RA) or rotifers enriched with protein hydrolysate (RA-PH); i.e. this was the first developmental stage where the RA-Zoo larvae were significantly longer and heavier. Thus, we selected RA-Zoo, RA and RA-PH larval samples at 9–10 mm length (26–30 dph, henceforth referred to as the 9 mm stage; see Methods for sampling details), with the goal of identifying zooplankton-responsive genes associated with the onset of the accelerated growth phase in the RA-Zoo group as compared with both other groups. The RA and RA-PH groups had virtually identical lengths and weights at this life-history stage [18], and thus, these groups were used in the current study as “non-zooplankton” groups for the purpose of identifying a robust suite of zooplankton-responsive genes. Information on the impact of incorporating zooplankton and PH into larval diets on cod growth and survival, as well as expression of well-known growth and appetite related genes (e.g. *IGF*, *myostatin*, *GH*, and *GHR*, and *NPY* and *CART*, respectively) can be found in Katan et al. [18]. These genes were not differentially expressed in RA-Zoo larvae as compared to both other groups at 9 mm [18], suggesting that other factors are involved in the initial acceleration in growth rate experienced by zooplankton-fed cod larvae.

We initially conducted whole-body transcript expression profiling with the 20,000 probe (20K) Atlantic cod oligonucleotide microarray platform [19], and then validated selected microarray-identified genes using real-time quantitative polymerase chain reaction (QPCR). Over 19,000 of the 20K microarray’s 50-mer oligonucleotide probes were designed based on unique candidate sequences arising from the assembly of over 150,000 ESTs from 42 cDNA libraries [23 normalized, 19 suppression subtractive hybridization (SSH)] representing various tissues, treatments, and life stages (including over 20,000 ESTs from a normalized larval cDNA library) [4, 19]. The EST assembly process (including steps taken to reduce redundancy), and the rationale for sequence selection and probe design for the 20K microarray platform, were previously described [4, 19]. Since it has been estimated that the Atlantic cod genome contains over 22,000 genes [20], the 20K microarray is necessarily missing some cod genes; however, this microarray platform has been effectively used for a variety of global transcript expression profiling applications [19, 21–24] and is a suitable resource for the current larval transcriptome study. While microarrays have been used to study larval transcriptomes for fish species including Atlantic halibut (*Hippoglossus hippoglossus* L.) and common sole (*Solea solea* L.) [25, 26], to our knowledge this is the first microarray- and QPCR-based study to identify and validate larval fish genes that respond to feeding with zooplankton.

Recently, RNA sequencing (RNAseq) was used to study the larval cod transcriptome response to a diet of copepods versus enriched rotifers [27, 28]. That RNAseq-based study identified 46 redox-relevant genes as significantly copepod-responsive, potentially contributing to enhanced growth [27, 28]. Similarities and discrepancies between the results of our study and the RNAseq study [27] will be discussed in light of the different rotifer enrichment methods that were employed (e.g. selenium enrichment in the RNAseq study [27, 28] versus no selenium enrichment in the current study).

## Results

### Larval dry mass

**Fig. 1:**
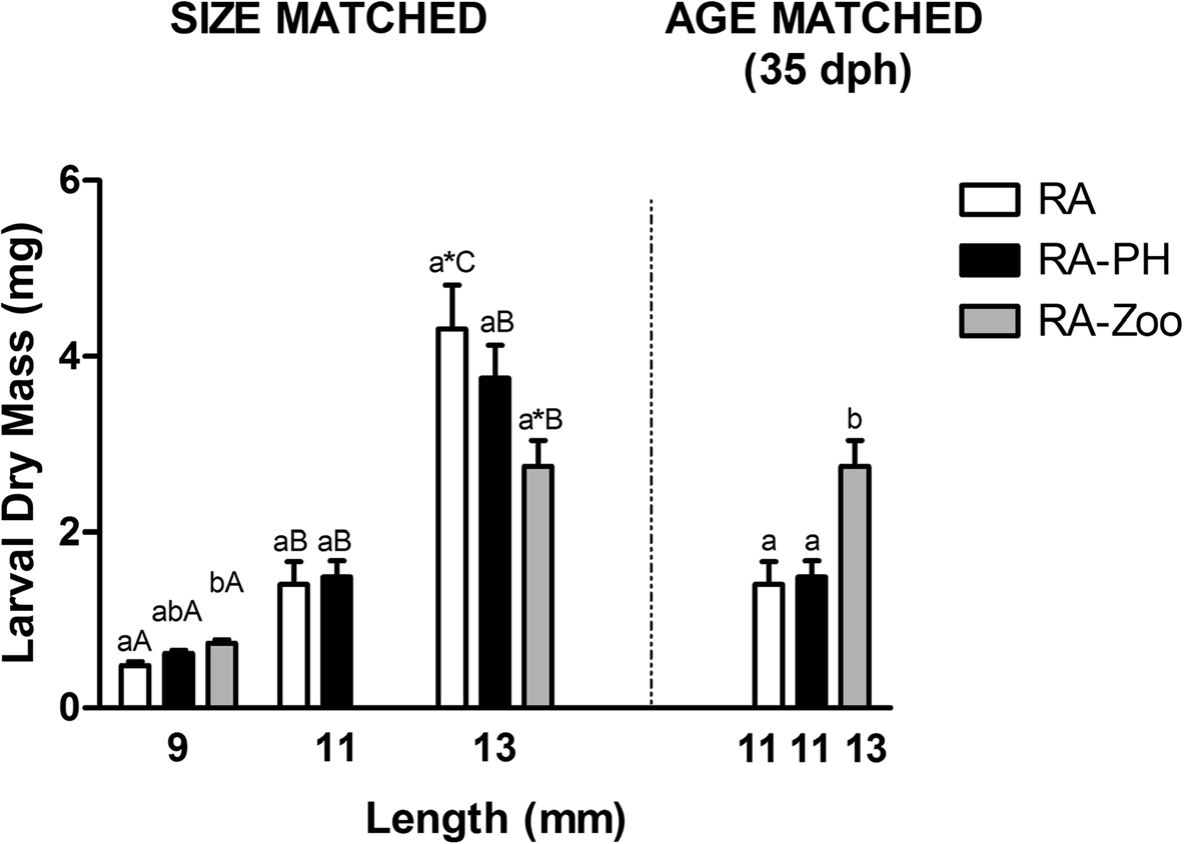
Larval dry mass data for larvae fed the different diets. Dissimilar lower case letters indicate a significant difference (*p* < 0.05) between treatments (RA; RA-PH; RA-Zoo) within a size-matched (~9 mm; ~ 11 mm; ~ 13 mm) or age-matched (34–35 dph) group. Dissimilar upper case letters indicate a significant difference (*p* < 0.05) between sizes (9 mm; 11 mm; 13 mm) within a treatment (RA; RA-PH; RA-Zoo) group. * indicates a difference at *p* = 0.051 between treatment groups marked. Values are means + 1 S.E

At the 9 mm stage sampling time (26–30 dph), the RA-Zoo larvae were significantly heavier than RA larvae, while RA-PH larval dry mass was not significantly different from either of the other two treatments (Fig. 1). When the larvae were 34–35 dph (i.e. “age-matched”; RA and RA-PH larvae ~11 mm, RA-Zoo larvae ~13 mm), the RA-Zoo larvae were significantly heavier than both RA and RA-PH larvae (Fig. 1). However, when they were size-matched (~13 mm), there were no significant differences in dry mass between the treatment groups although the RA versus RA-Zoo comparison approached significance (*p* = 0.051). Within each treatment (e.g. RA 9 mm vs. RA 11 mm vs. RA 13 mm), dry mass differed between sampling points, with one exception. This parameter was not significantly different between RA-PH larvae at 11 and 13 mm (Fig. 1).

### Microarray analysis of global transcript expression in ~9 mm cod larvae

**Fig. 2:**
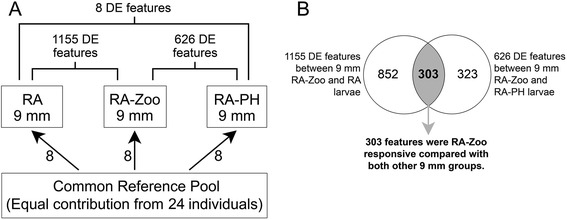
Overview of microarray experimental design and results. **a**. Reference design microarray experiment. Arrows represent microarrays with the numbers of biological replicates shown next to the arrows. The base of the arrow shows the Cy3-labeled sample (i.e. common reference pool), and the arrowhead shows the Cy5-labeled sample (i.e. experimental sample). The numbers of differentially expressed (DE) features between RA-Zoo and the other 2 treatment groups (FDR < 0.05) are shown. **b**. Venn diagram identifying microarray features that were DE between RA-Zoo and both RA and RA-PH larvae at 9 mm stage. Of the 303 overlapping genes, 147 were up-regulated in RA-Zoo and 156 genes were down-regulated in RA-Zoo compared to the RA and RA-PH groups (Additional file 1: Table S1 and Additional file 2: Table S2, respectively). Selected genes from the 303 gene list were included in the QPCR study (Tables 1 and 2)

The larval transcriptome was compared between treatment groups at the 9 mm stage. Between 9 mm RA and RA-Zoo larvae, 1155 differentially expressed (DE) microarray features were identified (FDR < 0.05), whereas 626 DE features were identified between 9 mm RA-PH and RA-Zoo larvae (Fig. 2a). In contrast, only 8 DE features were identified between 9 mm RA and RA-PH larvae. Since the aim of this study was to identify a robust set of RA-Zoo responsive genes in ~9 mm larvae, we used a Venn diagram approach to identify overlapping genes in the aforementioned 1155 and 626 gene lists. This identified 303 microarray features that were RA-Zoo responsive compared with both of the other 9 mm groups (Fig. 2b). Of these, 147 features were significantly up-regulated in RA-Zoo larvae compared with those in both the RA and RA-PH groups (Additional file 1: Table S1), and 156 features were significantly down-regulated in RA-Zoo larvae compared with both RA and RA-PH larvae (Additional file 2: Table S2). BLAST identification, gene ontology (GO) functional annotation based on Blast2GO (see Methods), and microarray fold change values for all 303 overlapping RA-Zoo responsive features are contained in Additional file 1: Table S1 and Additional file 2: Table S2.

### GO term enrichment analysis

**Fig. 3:**
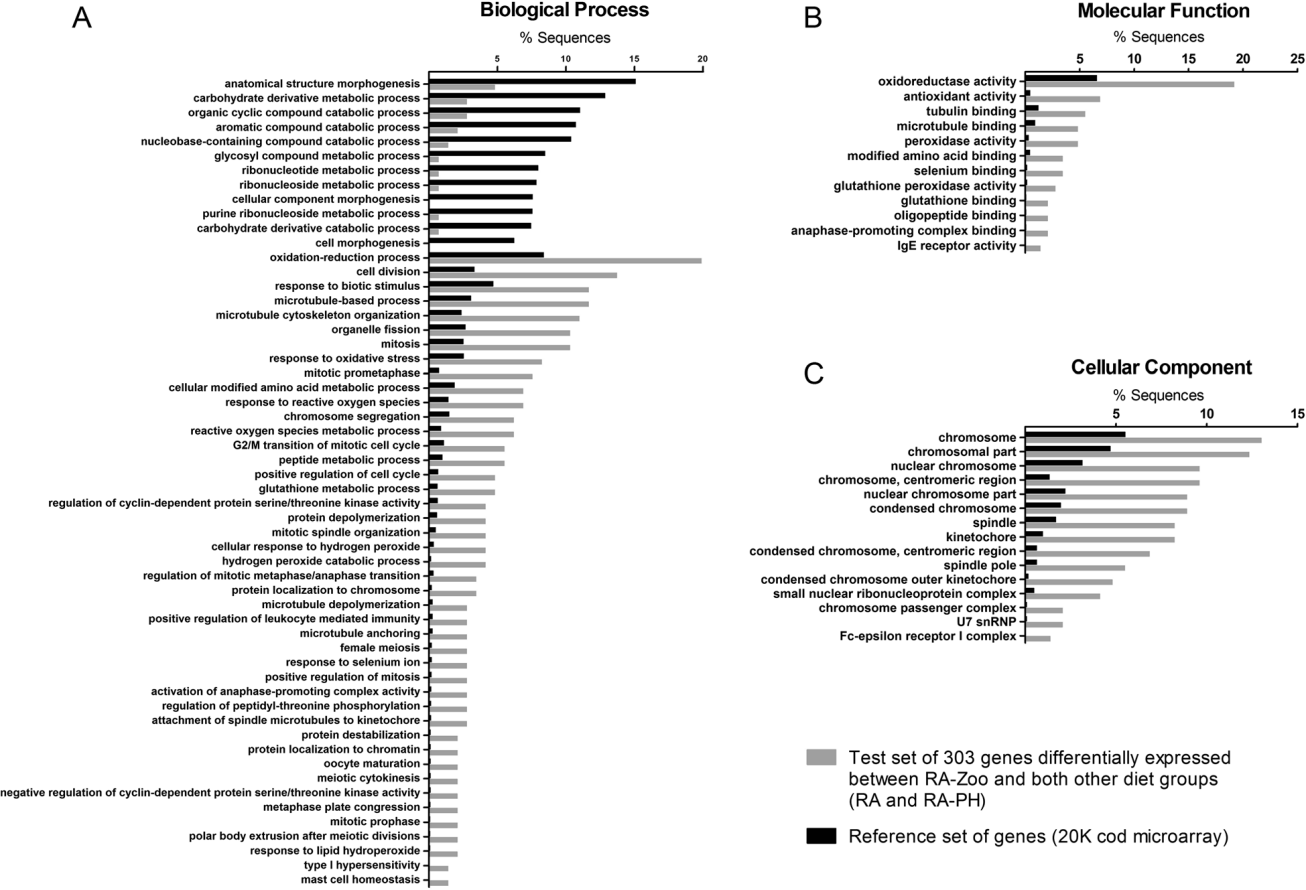
Gene ontology (GO) term enrichment analysis for the 303 overlapping RA-Zoo-responsive gene set. **a**. Enriched biological process GO terms. **b**. Enriched molecular function GO terms. **c**. Enriched cellular component GO terms. Selected enriched GO terms are included in this figure, and a complete list of enriched GO terms is available in Additional file 3: Table S3

GO Biological Process (BP) terms that were underrepresented in the set of 303 RA-Zoo responsive genes compared with the 20K cod microarray included “anatomical structure morphogenesis”, “aromatic compound catabolic process”, “ribonucleotide metabolic process”, and “carbohydrate derivative catabolic process” (Fig. 3a). GO BP terms overrepresented in the RA-Zoo responsive gene set included several related to cell division (e.g. “cell division”, “mitosis”, “chromosome segregation”, “mitotic spindle organization”, “positive regulation of cell cycle”, “mitotic prometaphase”) and oxidation-reduction (e.g. “oxidation-reduction process”, “response to oxidative stress”, “response to reactive oxygen species”, “reactive oxygen species metabolic process”) (Fig. 3a). In the Molecular Function (MF) and Cellular Component (CC) categories, all enriched GO terms were overrepresented in the RA-Zoo responsive gene set compared with the reference gene set; this included GO MF terms “oxidoreductase activity”, “antioxidant activity”, “tubulin binding”, “peroxidase activity”, and “selenium binding” (Fig. 3b), and GO CC terms primarily involved in cell division (e.g. “chromosome”, “spindle”, “kinetochore”) (Fig. 3c). Selected GO terms are shown in Fig. 3, and all enriched GO terms are provided in Additional file 3: Table S3.

### Hierarchical clustering analyses

**Fig. 4:**
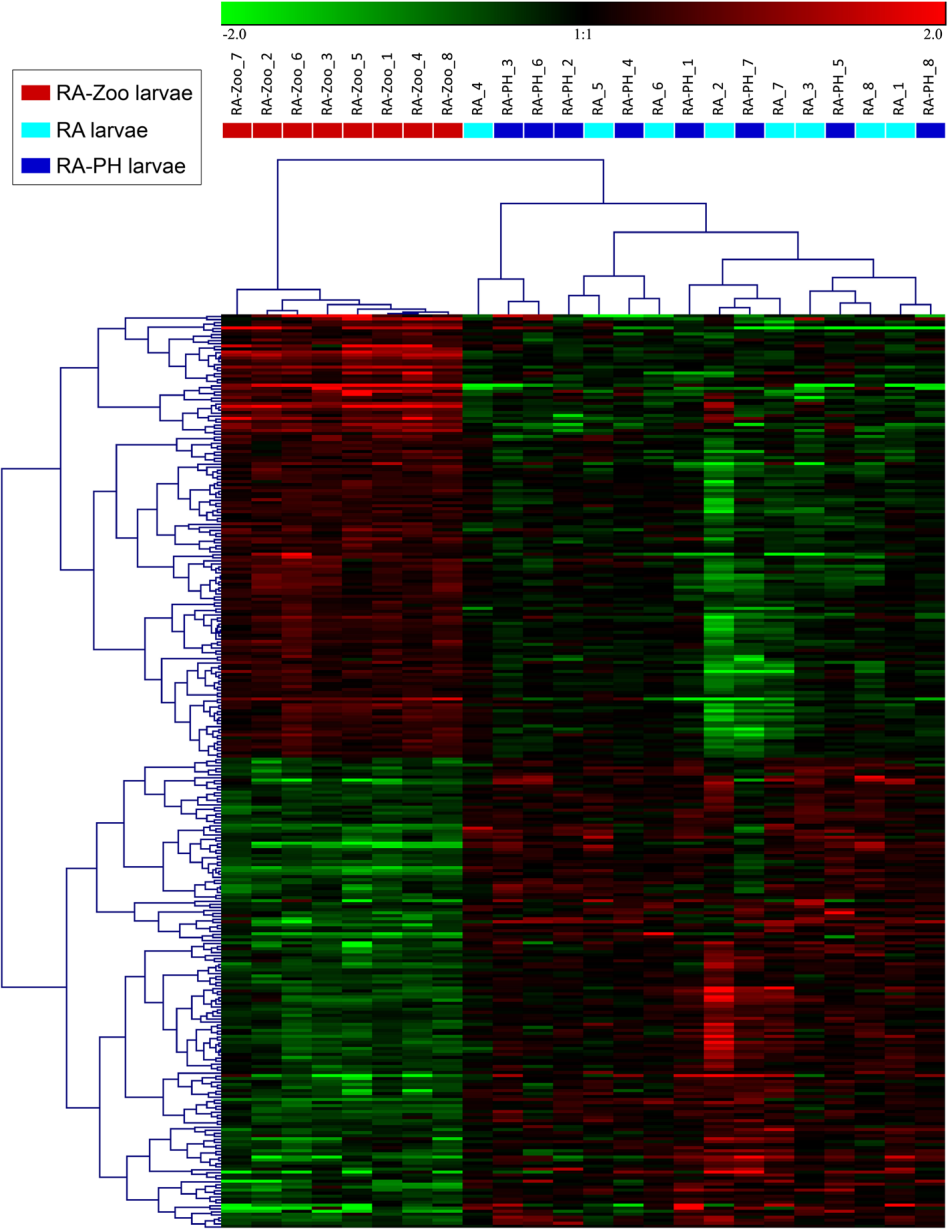
Hierarchical clustering analysis of zooplankton-responsive genes. RA-Zoo, RA, and RA-PH (*n* = 8 each) samples were clustered based on a collection of 303 genes that were differentially expressed (FDR < 0.05) between RA-Zoo and both RA and RA-PH. Genes were median-centered and clustered using Pearson correlation and complete linkage hierarchical clustering. Coloured blocks indicate treatment groups (see legend)

Hierarchical clustering was performed on the collection of 303 genes that were differentially expressed (FDR < 0.05) between RA-Zoo larvae and both RA larvae and RA-PH larvae to determine if treatment groups could be separated based on gene expression profiles. In this analysis, all RA-Zoo larvae clustered in a separate branch from all other larvae (as expected, since SAM analysis identified these genes as RA-Zoo responsive). However, among the RA and RA-PH larvae, there were no separate subclusters for treatment (Fig. 4). This indicates that there was no discernible impact of RA or RA-PH treatments on expression of the complete set of 303 RA-Zoo responsive genes.

**Fig. 5:**
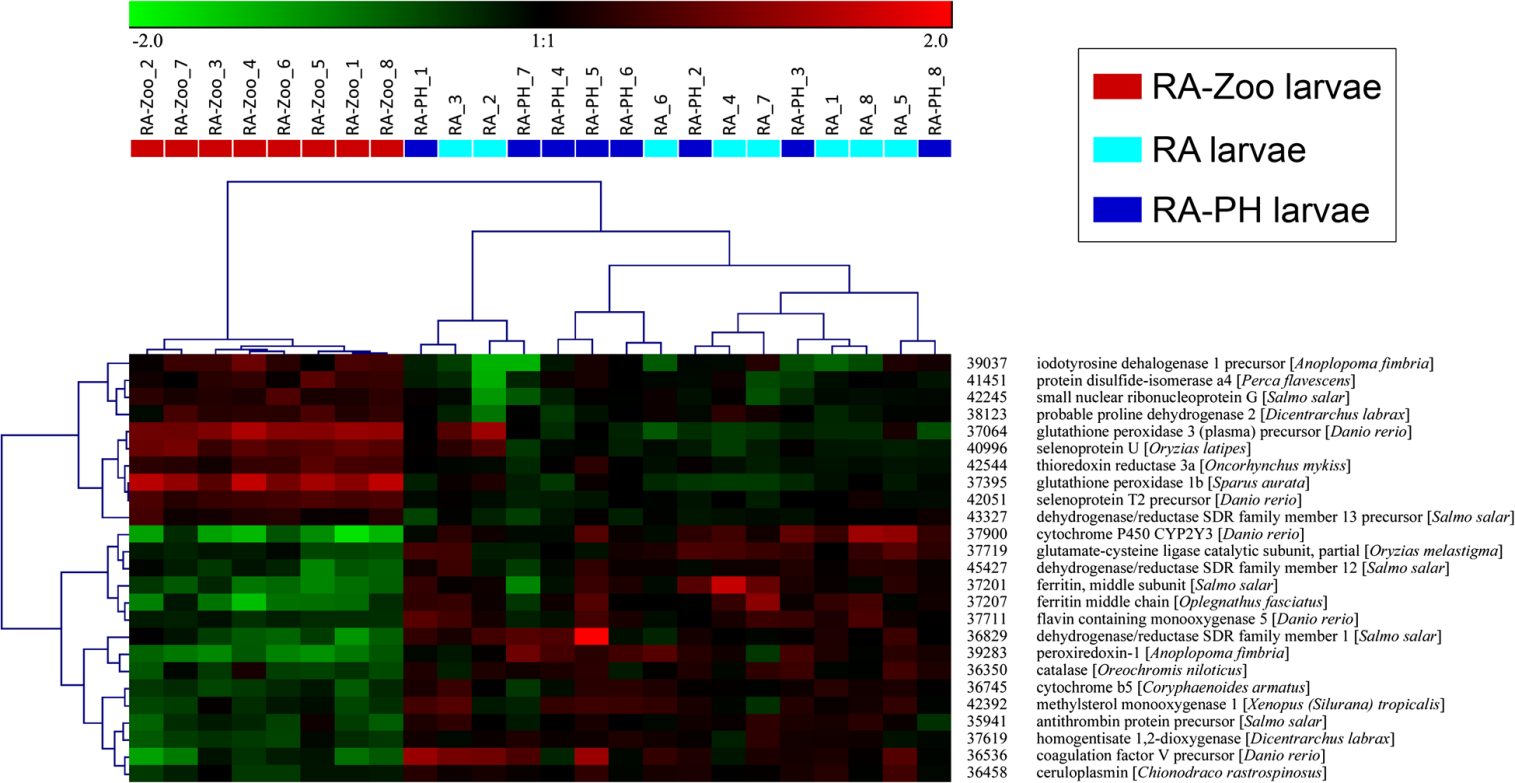
Hierarchical clustering analysis for a subset of RA-Zoo responsive genes involved in oxidation-reduction. RA-Zoo, RA and RA-PH (*n* = 8 each) samples were clustered based on features having the associated GO terms “oxidation-reduction process” and/or “cell redox homeostasis”. Gene names were taken from the most significant BLASTx hits with associated protein names (avoiding “predicted” and “unnamed” hits if possible)

**Fig. 6:**
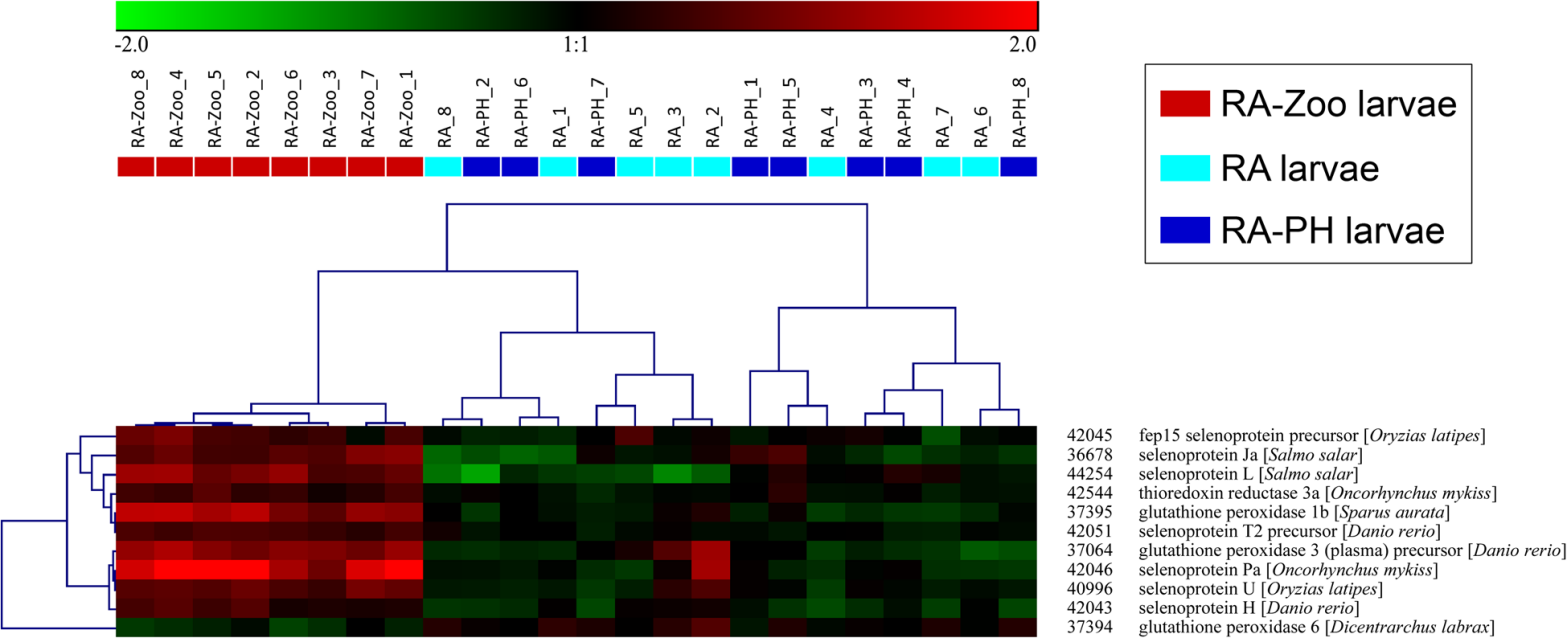
Hierarchical clustering analysis for a subset of RA-Zoo responsive genes potentially involved in selenium homeostasis. RA-Zoo, RA and RA-PH (n = 8 each) samples were clustered based on features having the associated GO terms “response to selenium ion” and/or “selenium binding”, and five additional selenoprotein-encoded transcripts were also included in this analysis. Gene names were taken from the most significant BLASTx hits with associated protein names (avoiding “predicted” and “unnamed” hits if possible)

**Fig. 7:**
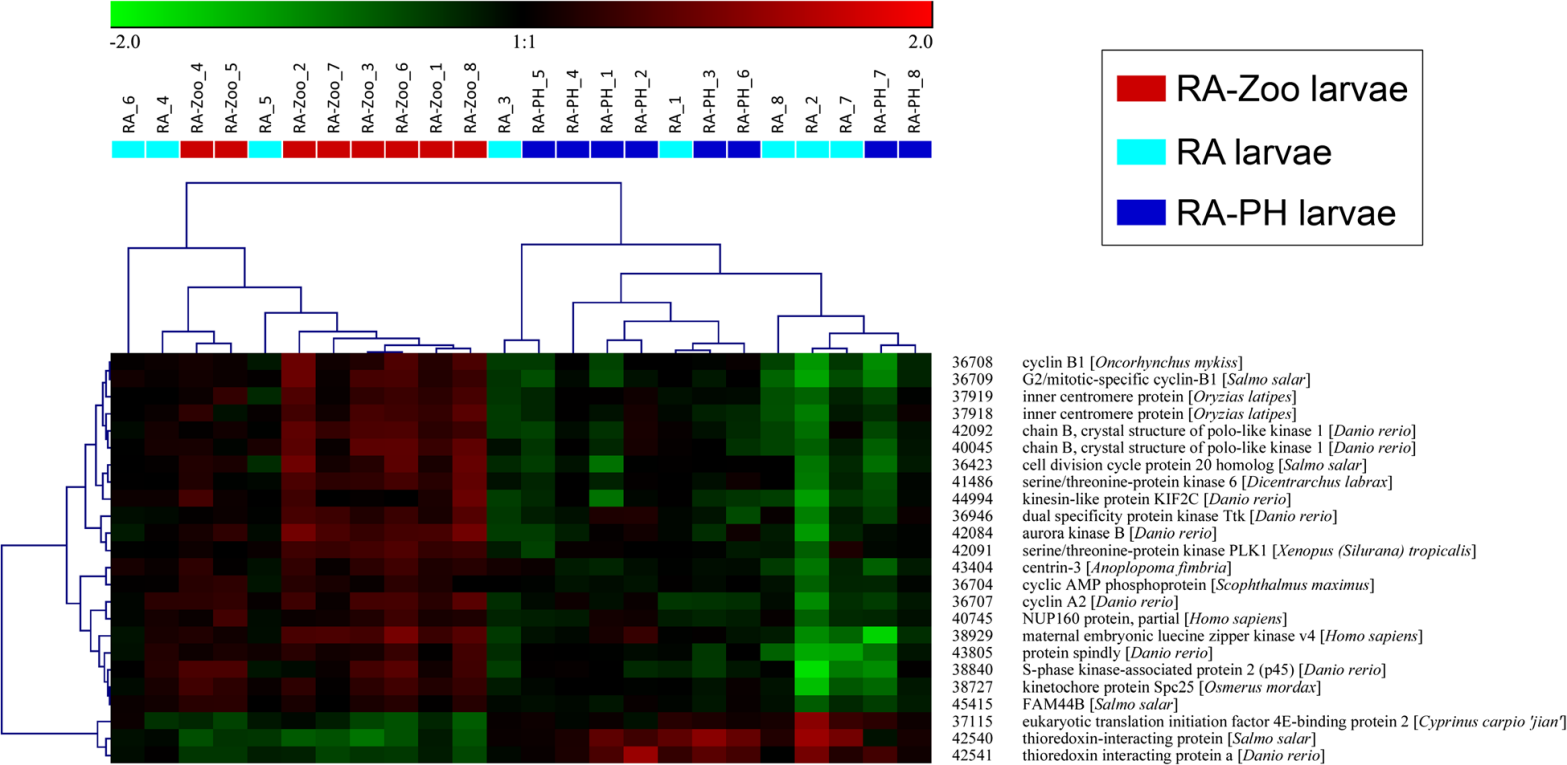
Hierarchical clustering analysis for a subset of RA-Zoo responsive genes involved in mitosis. RA-Zoo, RA and RA-PH (*n* = 8 each) samples were clustered based on features having the associated GO terms “mitosis”, “cell division”, and/or any term including the word “mitotic”. Gene names were taken from the most significant BLASTx hits with associated protein names (avoiding “predicted” and “unnamed” hits if possible)

Hierarchical clustering was performed for subsets of the 303 genes identified in the GO term enrichment analysis. Since large numbers of enriched GO terms pertained to oxidation-reduction and cell division, genes with these functional annotations (e.g. *iodotyrosine dehalogenase 1*, *cytochrome P450 CYP2Y3*, *ferritin middle subunit*, *dehydrogenase/reductase SDR family member 1*) were included in the hierarchical clustering analyses. Further, since selenium binding was an overrepresented GO term (Fig. 3b) suggesting that selenium homeostasis may be involved in the larval cod response to zooplankton, RA-Zoo responsive genes with selenium-relevant functional annotations (and other selenoprotein-encoding genes) were subjected to hierarchical clustering. In the hierarchical clustering analyses of genes involved in oxidation-reduction (Fig. 5) and selenium homeostasis (Fig. 6), all RA-Zoo larvae clustered separately from all other larvae; among RA and RA-PH larvae, there were no separate subclusters for treatment. These results demonstrate that the zooplankton diet had a considerable and consistent impact on larval expression of genes involved in these processes, while the other treatments did not have a discernible impact. Note that there were some genes in common between the oxidation-reduction and selenium homeostasis clustering studies (e.g. *glutathione peroxidase 3*, *selenoprotein U*, *selenoprotein T2*) (Figs. 5 and 6). In the hierarchical clustering analysis involving mitosis-relevant genes (e.g. *cyclin B1*, *cyclin A2*, *aurora kinase B*, *inner centromere protein*), three RA larvae grouped with the RA-Zoo in a branch separate from the remaining RA and RA-PH larvae (Fig. 7). Thus, compared with oxidation-reduction and selenium homeostasis, the influence of the zooplankton diet on mitosis appears to be less consistent.

Of the 303 overlapping RA-Zoo responsive genes, approximately half (147 genes) were up-regulated by zooplankton and approximately half (156 genes) were down-regulated by this dietary treatment (Fig. 4; Additional file 1: Table S1 and Additional file 2: Table S2). Of the 25 genes included in the oxidation-reduction clustering analysis, 10 genes were up-regulated and 15 genes were down-regulated in response to zooplankton (Fig. 5). Unlike the results for all RA-Zoo responsive genes or oxidation-reduction relevant genes, nearly all of the genes included in the selenium homeostasis (10 out of 11) and mitosis (21 out of 24) clustering analyses were up-regulated in RA-Zoo larvae compared with the other two treatment groups (Figs. 6 and 7).

### RA-Zoo responsive genes involved in the thyroid hormone (TH) biosynthesis pathway

The microarray experiment identified two genes involved in TH production [*iodotyrosine dehalogenase I precursor* (alias *iodotyrosine deiodinase*) and *putative iodothyronine deiodinase type I*] that were significantly up-regulated in zooplankton-fed larvae as compared with both other groups (Additional file 1: Table S1). *Iodotyrosine dehalogenase I precursor*, associated with GO BP terms including thyroid hormone generation and oxidation-reduction process, was 1.71 and 1.54 fold higher expressed in RA-Zoo versus RA and RA-PH larvae, respectively. *Putative iodothyronine deiodinase type I*, with GO BP terms including hormone metabolic process and cellular metabolic process, was 1.49 and 1.86 fold higher expressed in RA-Zoo versus RA and RA-PH larvae, respectively (Additional file 1: Table S1).

### QPCR analysis

**Fig. 8:**
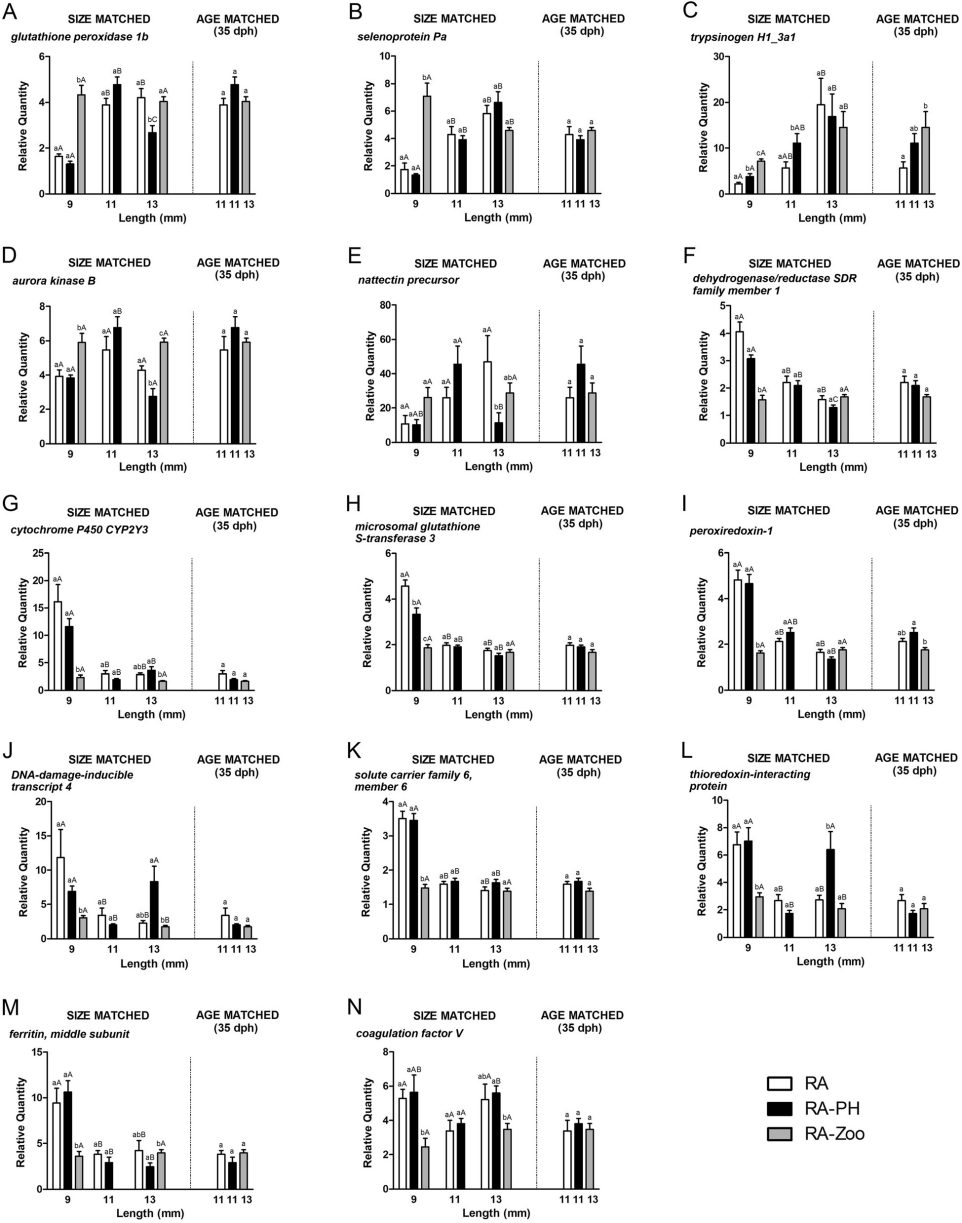
QPCR results for selected zooplankton-responsive genes-identified by microarray. Dissimilar lower case letters indicate a significant difference (*p* < 0.05) between treatments (RA; RA-PH; RA-Zoo) within a size-matched (~9 mm; ~ 11 mm; ~ 13 mm) or age-matched (34–35 dph) group. Dissimilar upper case letters indicate a significant difference (*p* < 0.05) between sizes (9 mm; 11 mm; 13 mm) within a treatment group.  A) *glutathione peroxidase 1b*; B) *selenoprotein Pa*; C) *trypsinogen H1_3a1*; D) *aurora kinase B*; E) *nattectin precursor*; F) *dehydrogenase/reductase SDR family member 1*; G) *cytochrome P450 CYP2Y3*; H) *microsomal glutathione S-transferase 3*; I) *peroxiredoxin-1*; J) *DNA-damage-inducible transcript 4*; K) *solute carrier family 6, member 6*; L) *thioredoxin-interacting protein*; M) *ferritin, middle subunit*; N) *coagulation factor V*.

Fourteen microarray-identified genes, representing a variety of functional annotations (Table 1), were selected for QPCR studies involving 9–13 mm larvae templates. BLAST identification, manually collected gene ontology (GO) functional annotations associated with the best named BLASTx hits, and microarray and QPCR fold change values for this set of 14 genes are contained in Table 1. Of these, 5 genes were up-regulated and 9 genes were down-regulated in RA-Zoo compared with both other treatment groups at the 9 mm stage (Table 1). Four of the five up-regulated genes selected for QPCR (*glutathione peroxidase 1b*, *selenoprotein Pa*, *trypsinogen H1_3a1*, and *aurora kinase B*) were QPCR validated as significantly up-regulated in RA-Zoo compared with both other treatment groups at ~9 mm (Fig. 8a-d). While the microarray and QPCR results for the transcript encoding Nattectin precursor agreed in direction of change, this gene was not considered to be validated as QPCR showed its expression level in 9 mm RA-Zoo, RA, and RA-PH larvae was not significantly different (Table 1; Fig. 8e). As shown by QPCR, the most highly up-regulated gene in 9 mm RA-Zoo compared with both other 9 mm groups was *selenoprotein Pa* (4.10 and 5.34 fold higher than RA and RA-PH groups, respectively) (Table 1; Fig. 8b). All nine of the genes identified by microarray as being suppressed in zooplankton-fed larvae were QPCR validated as significantly down-regulated in RA-Zoo larvae compared with both RA and RA-PH larvae at the 9 mm stage (Fig. 8f-n). QPCR showed that the most highly down-regulated gene in 9 mm RA-Zoo larvae compared with both other 9 mm groups was *cytochrome P450 CYP2Y3* (6.94 and 4.99 fold lower than in RA and RA-PH larvae, respectively) (Table 1; Fig. 8g).

**Table 1:** Identification and transcript expression results for 14 microarray-identified features selected for QPCR study^a^

Microarray probe ID^b^	Protein name (species affiliation, GenBank accession number, and associated E-value for the best named BLASTx hit)^c^	Functional annotation associated with best named BLASTx hit^d^	Microarray fold change in RA-Zoo versus:		QPCR fold change in RA-Zoo versus^e^:	
			RA	RA-PH	RA	RA-PH
37395	Glutathione peroxidase 1b (*Sparus aurata*, AFY97791, 1.81E-101)	Response to oxidative stress (BP); oxidation-reduction process (BP); peroxidase activity (MF); glutathione peroxidase activity (MF); oxidoreductase activity (MF).	2.57	2.68	2.66	3.33
42046	Selenoprotein Pa (*Oncorhynchus mykiss*, CCX35038, 1.97E-54)	NA	3.31	3.89	4.10	5.34
42811	Trypsinogen H1_3a1 (*Dissostichus mawsoni*, AEA08590, 2.07E-120)	Proteolysis (BP); catalytic activity (MF); serine-type endopeptidase activity (MF); hydrolase activity (MF).	5.62	3.40	3.28	1.88
42084	Aurora kinase B (*Danio rerio*, NP_997731, 2.47E-33)	ATP binding (MF); histone serine kinase activity (MF); metal ion binding (MF); cellular response to UV (BP); chromosome segregation (BP); mitotic cytokinesis (BP); spindle midzone assembly involved in mitosis (BP); multicellular organismal development (BP); negative regulation of B cell apoptotic process (BP); histone H3-S28 phosphorylation (BP); transferase activity (MF)^f^.	1.79	1.59	1.51	1.54
46230	Nattectin precursor (*Anoplopoma fimbria*, ACQ58341, 7.10E-31)	Carbohydrate binding (MF).	2.04	2.13	2.45	2.58
36829	Dehydrogenase/reductase SDR family member 1 (*Salmo salar*, NP_001134326; 1.87E-59)	Metabolic process (BP); oxidation-reduction process (BP); oxidoreductase activity (MF).	−1.60	−2.29	−2.58	−1.96
37900	Cytochrome P450 CYP2Y3 (*Danio rerio*, AAX37329, 6.47E-90)^g^	Oxidation-reduction process (BP); monooxygenase activity (MF); iron ion binding (MF); oxidoreductase activity, acting on paired donors, with incorporation or reduction of molecular oxygen (MF); heme binding (MF).	−3.14	−2.61	−6.94	−4.99
38166	Microsomal glutathione S-transferase 3 (*Oncorhynchus mykiss*, ACO07847, 1.25E-73)	Metabolic process (BP); transferase activity (MF).	−1.88	−1.99	−2.45	−1.79
39283	Peroxiredoxin-1 (alias Natural killer enhancing factor) (*Anoplopoma fimbria*, ACQ58049, 3.73E-125)	Oxidation-reduction process (BP); antioxidant activity (MF); oxidoreductase activity (MF); peroxiredoxin activity (MF).	−2.05	−2.51	−2.99	−2.89
41986	DNA-damage-inducible transcript 4 protein (alias REDD1; RTP801) (*Dicentrarchus labrax*, CBN81525, 9.89E-88)	Negative regulation of signal transduction (BP).	−3.06	−1.89	−3.88	−2.26
42481	Solute carrier family 6, member 6 (alias Sodium- and chloride-dependent taurine transporter) (*Salmo salar*, NP_001117102, 1.53E-104)	Transport (BP); neurotransmitter transport (BP); organic acid transport (BP); transmembrane transport (BP); neurotransmitter:sodium symporter activity (MF); taurine:sodium symporter activity (MF).	−1.84	−1.77	−2.37	−2.33
42540	Thioredoxin-interacting protein (*Salmo salar*, ACN10667, 1.80E-92)	NA	−2.02	−2.10	−2.29	−2.38
37201	Ferritin, middle subunit (*Salmo salar*, ACI66864, 2.69E-12)	Iron ion transport (BP); cellular iron ion homeostasis (BP); ferric iron binding (MF).	−2.25	−1.86	−2.61	−2.95
36536	Coagulation factor V (*Danio rerio*, AAN71005, 9.03E-15)	Cell adhesion (BP); copper ion binding (MF).	−1.98	−2.04	−2.15	−2.30

*NA* not applicable (i.e. no functional annotation found for best named BLASTx hit)

^a^These 14 genes were selected from the list of 303 microarray features that were RA-Zoo responsive compared with both RA and RA-PH in 9 mm stage samples. The complete list of 303 microarray features is available in Additional file 1: Table S1 (147 genes significantly up-regulated in RA-Zoo compared with both RA and RA-PH) and Additional file 2: Table S2 (156 genes significantly down-regulated in RA-Zoo compared with both RA and RA-PH)

^b^Probe identifier (ID) numbers are 5-digit unique identifiers for the 50mer probes on the Atlantic cod 20K microarray [19]

^c^The BLASTx hits with the lowest E-values and protein names (e.g. not “unnamed protein product” or “predicted”) are shown for the Atlantic cod expressed sequence tag (EST) contiguous sequences (contigs) or singletons used for probe design [19]

^d^Functional annotation associated with the best named BLASTx hit of the Atlantic cod cDNA sequence represented by the informative microarray probe. Biological Process (BP) and Molecular Function (MF) Gene Ontology (GO) terms are listed in this table. If multiple, similar terms were available, a representative term was selected for inclusion in this table

^e^QPCR mean fold-change was calculated as mean RA-Zoo relative quantity (RQ) divided by mean RA or RA-PH RQ for genes up-regulated in RA-Zoo, and as mean RA or RA-PH RQ divided by mean RA-Zoo RQ for genes down-regulated in RA-Zoo (indicated by a negative sign). All genes in this table except Nattectin were QPCR validated as significantly (*p* < 0.05) differentially expressed between RA-Zoo and both RA and RA-PH larvae at the 9 mm stage

^f^Since there are over 20 GO terms associated with this sequence, a selection of GO terms are included in this table

^g^The best named hit was Cytochrome P450 precursor (*Danio rerio*, NP_001018658). The second-best hit is listed as it has a more descriptive name and a similar E-value

In age-matched older larvae (~11 mm length for RA and RA-PH, ~13 mm length for RA-Zoo), 12 out of 14 genes subjected to QPCR showed no significant difference in expression between treatment groups; the exceptions to this were *trypsinogen H1_3a1* (higher expressed in RA-Zoo compared with RA larvae) and *peroxiredoxin-1* (with lower expression in RA-Zoo as compared with RA-PH larvae) (Fig. 8).

In size-matched larvae (~13 mm in length), 6 out of 14 genes subjected to QPCR showed no significant difference in expression between treatment groups (Fig. 8b,c,f,h,i,k). Of the remaining genes subjected to QPCR, one gene (*aurora kinase B*) was significantly up-regulated in RA-Zoo larvae compared with both other treatment groups (Fig. 8d), and two genes (*cytochrome P450 CYP2Y3* and *coagulation factor V*) were significantly down-regulated in the RA-Zoo larvae compared with the RA-PH group alone despite there being no significant difference in transcript expression between RA and RA-PH larvae (Fig. 8g,n). Five genes in size-matched 13 mm larvae appeared to respond to the RA-PH treatment (i.e. they were differentially expressed between RA-PH and at least one of the other groups, with no significant difference between RA and RA-Zoo expression). Two of these genes (*DNA-damage-inducible transcript 4*, *thioredoxin-interacting protein*) were up-regulated and 3 genes (*glutathione peroxidase 1b*, *nattectin*, *ferritin middle subunit*) were down-regulated in 13 mm RA-PH larvae compared with other size-matched groups (Fig. 8a,e,j,l,m).

## Discussion

The microarray component of this study revealed that suites of genes involved in biological processes including oxidation-reduction, selenium binding, mitosis, and thyroid hormone generation were responsive in 9–10 mm cod larvae (herein referred to as the 9 mm stage) that were fed wild zooplankton. However, the QPCR study showed that most of the genes identified as differentially expressed in the 9 mm larvae were no longer zooplankton-responsive in age-matched (34–35 dph; ~11 mm in length for RA and RA-PH groups, and ~13 mm in length for the faster-growing RA-Zoo group) or size-matched (all ~13 mm) larvae with the exception of: 1) *trypsinogen H1_3a1* (associated with GO terms including “proteolysis” and “serine-type endopeptidase activity”, up-regulated in RA-Zoo) and *peroxiredoxin-1* (involved in oxidation-reduction process, down-regulated in RA-Zoo) in the age-matched groups; and 2) 3 genes in the size-matched 13 mm comparison which showed the same direction of expression change as was observed in 9 mm larvae (*aurora kinase B*, associated with GO terms including “spindle midzone assembly involved in mitosis” and “multicellular organismal development”, up-regulated in RA-Zoo larvae; *cytochrome P450 CYP2Y3* and *coagulation factor V*, involved in oxidation-reduction process, both down-regulated in RA-Zoo larvae). These findings suggest that, while genes identified and validated in this study are likely involved in the initiation of the enhanced growth phenotype in zooplankton-fed larvae (i.e. at 9 mm), only a subset of these genes appear to be zooplankton-responsive (and potentially involved in sustaining enhanced growth) in older larvae. Genes analyzed by QPCR that were RA-Zoo-responsive only in 9 mm larvae and not differentially expressed between groups of older larvae include *selenoprotein Pa* (associated with GO terms including “response to selenium ion”, “selenium binding”, and “response to oxidative stress”, up-regulated in 9 mm RA-Zoo larvae), and three genes that were down-regulated in 9 mm RA-Zoo larvae (*dehydrogenase/reductase SDR family member 1*, associated with GO terms including “metabolic process” and “oxidation-reduction process”; *microsomal glutathione S-transferase 3*, associated with GO terms “metabolic process” and “transferase activity”; and *solute carrier family 6, member 6* (*slc6a6*; alias: Na^+^-Cl^−^-dependent taurine transporter), associated with GO terms including “neurotransmitter transport”, “organic acid transport”, and “taurine:sodium symporter activity” (Fig. 8b,f,h,k; Table 1; Additional file 1: Table S1). The zooplankton-responsiveness of some genes at 9 mm, but not at the 11–13 mm stage, may be due to the fact that 9 mm larvae were fed rotifers while 11–13 mm larvae were fed *Artemia* (with RA-Zoo receiving supplemental zooplankton throughout this developmental period), and *Artemia* are more similar to zooplankton than are rotifers for several important nutrients such as selenium and taurine (reviewed by Hamre et al. [29]).

As previously mentioned, Penglase et al. [27] used RNAseq to investigate transcript expression in cod larvae that were fed zooplankton versus rotifers. Unlike our study, which used Ori-Culture and Ori-Green (Skretting) enrichment of rotifers with no selenium or other enrichment (see Methods for details), the RNAseq-based study used new enrichment protocols that provide rotifers with ‘zooplankton levels’ of some nutrients (e.g. selenium, manganese, copper) but not others (e.g. iodine, zinc, taurine) [28]. Similar to our study, the RNAseq-based study found that there was no significant difference in the growth of cod larvae fed zooplankton versus rotifers up to 22 dph, with zooplankton-fed larvae experiencing significantly accelerated growth between 22 and 36 dph [27, 28]. The RNAseq-based study evaluated larval growth, nutrient levels, and gene expression at 5 larval stages, with stage 3 (29–31 dph, 8.5–10.1 mm length) [27, 28] approximately corresponding to the 9 mm stage in our microarray and QPCR studies. Some of the stage 3 gene expression results from Penglase et al. [27] are similar to our microarray results; for example, *thioredoxin interacting protein a* (*txnipa*), *catalase*, and *glutathione-cysteine ligase catalytic subunit* were down-regulated (significantly for all 3 genes in our study and for *txnipa* in the RNAseq study) in zooplankton-fed ~ 9 mm larvae in both studies (Additional file 2: Table S2; [27]). Also, both studies found that the larval redox system and growth rate were strongly influenced by feeding with copepods (even though our study included only 5–10 % zooplankton). However, other results from Penglase et al. [27] do not agree with our results. In our study, *glutathione peroxidase 1b* (*gpx1b*) and *selenoprotein Pa* (*sepp1a*) were significantly up-regulated in 9 mm RA-Zoo larvae (Fig. 8a,b), whereas Penglase et al. [27] found that *gpx1a* and *sepp1a* were down-regulated (not significantly) in zooplankton-fed ~ 9 mm cod larvae. Since glutathione peroxidases and selenoprotein P are selenium-dependent (see subsequent section), differences in their transcript expression may have been influenced by differences in rotifer enrichment methods utilized in the RNAseq-based study versus our study. In the RNAseq study, rotifer enrichment included Sel-Plex (Alltech, Denmark), a selenium-supplemented yeast, whereas in our study, rotifer enrichment included only Ori-Culture and Ori-Green (Skretting) without a selenium supplement. Therefore, the 9 mm RA and RA-PH larvae in our study may have been deficient in selenium, while the comparably staged rotifer-fed larvae in the RNAseq study were selenium replete due to the rotifer enrichment methods [27, 28]. Even with adequate levels of selenium, however, the rotifer-fed cod larvae in the RNAseq study still grew more slowly than the zooplankton-fed larvae, leading Karlsen and colleagues to hypothesize that protein and taurine (which were present at lower levels in rotifers compared with copepods in that study) may be involved in copepod-associated enhanced growth [28].

In our study, the expression results for *slc6a6* support the notion that taurine plays a role in the enhanced growth of zooplankton-fed cod larvae at the 9 mm stage. This taurine transporter-encoding transcript was shown by microarray and QPCR to be significantly down-regulated in RA-Zoo larvae compared with both other groups at 9 mm (Table 1; Fig. 8k). In Atlantic salmon (*Salmo salar*), *slc6a6* was shown by microarray to be significantly up-regulated in distal intestine during the early response (day 3) to a soybean meal containing diet, and therefore, potentially associated with the development of soybean meal induced enteritis [30]. Therefore, the higher expression of *slc6a6* in both non-zooplankton groups (i.e. RA and RA-PH) suggests that the groups fed only rotifers may have had an inflammatory reaction. In Japanese eel (*Anguilla japonica*) testis during hCG-induced spermatogenesis, higher Slc6a6 transcript and protein expression levels are associated with lower taurine levels [31]. This inverse relationship between *slc6a6* expression and taurine agrees with our study. Rotifers have lower levels of taurine than *Artemia* or zooplankton (reviewed by Hamre et al. [29]), and *slc6a6* expression was higher in cod larvae fed rotifers (9 mm, likely taurine deficient) but not in older cod larvae fed *Artemia* (13 mm, likely taurine replete) (Fig. 8k). Interestingly, trypsin promotes the uptake of taurine into eel germ cells by transactivating Slc6a6, and *trypsinogen H1-3a1* was shown in our study to be induced in zooplankton-fed cod larvae. The potential roles of taurine, taurine transporter (Slc6a6), and trypsin in copepod-associated enhanced growth of cod larvae warrant further investigation.

Since the zooplankton-associated enhanced growth phenotype is first apparent when cod larvae are approximately 9–10 mm long [18, 28], we propose that transcriptome changes in zooplankton-fed larvae as compared with other larvae at ~9 mm provide significant insight into the mechanisms involved in the initiation of accelerated growth. In addition to oxidation-reduction, selenium-binding, and mitosis, the microarray experiment identified other key growth-relevant genes (e.g. involved in thyroid hormone production) as zooplankton-responsive. The remainder of the discussion will focus on how these genes and pathways, and their potential interactions, may be involved in enhanced growth.

### Influence of zooplankton diet on oxidation-reduction processes and selenium homeostasis

Wild zooplankton (e.g. copepods) are high in LC-PUFA such as DHA and EPA [15, 32]. Likewise, cultured marine fish larvae fed wild zooplankton have higher levels of LC-PUFA than larvae without zooplankton in the diet [14, 33, 34], and wild cod larvae also have high levels of DHA and EPA [32]. High levels of LC-PUFA in marine fish larvae, along with other factors (e.g. increased metabolic rate and oxygen demand), make this life stage particularly vulnerable to molecular and cellular damage caused by oxidative stress [35]. The antioxidant system, including enzymes such as catalase and glutathione peroxidase, is key to protecting larval fish from reactive oxygen species generated by cellular metabolism [35]. The current microarray experiment showed that genes encoding several important antioxidant enzymes and other proteins involved in redox homeostasis were responsive to the feeding of zooplankton at the 9 mm sampling time point. This agrees at the pathway level with the RNAseq results of Penglase et al. [27], who showed that 46 redox-relevant genes were differentially expressed in cod larvae that were fed zooplankton versus rotifers. QPCR confirmed our microarray results, and revealed that the influence of dietary zooplankton on the transcription of genes related to the antioxidant system in larval cod was less apparent in 11–13 mm larvae. All of the genes in the QPCR study that were annotated with the GO term “oxidation-reduction process” and/or “cell redox homeostasis” (i.e. *glutathione peroxidase*, *dehydrogenase/reductase SDR family member 1*, *cytochrome P450 CYP2Y3, peroxiredoxin-1*, *ferritin middle subunit*, and *coagulation factor V*) were differentially (*p* < 0.05) expressed between zooplankton-fed larvae and both of the other two larval groups at 9 mm, while none of these genes were differentially expressed between RA-Zoo larvae and both RA and RA-PH larvae in the size- or age-matched comparisons (i.e. at 11–13 mm). As previously stated, this may be due to dietary differences between the 11–13 mm larvae (fed *Artemia* with or without zooplankton) and the 9 mm larvae (fed rotifers with or without zooplankton); the rotifers used in our study were potentially deficient in some nutrients (e.g. selenium, copper, taurine) that are known to be at higher levels in *Artemia* and zooplankton compared with rotifers (reviewed by Hamre et al. [29]).

In Asian seabass (*Lates calcarifer*) selenium-dependent glutathione peroxidase activity and lipid peroxidation increase through larval development, with glutathione peroxidase likely serving to detoxify lipid peroxides [36]. Sea bass larvae fed high-DHA diets have elevated glutathione peroxidase transcript expression levels compared with larvae fed a lower DHA diet [35]. Our finding that *gpx1b* was significantly up-regulated (i.e. by over two-fold) in the zooplankton-fed cod larvae compared with the non-zooplankton groups at 9 mm, but not responsive to dietary zooplankton in 11–13 mm larvae, suggests that the 9 mm cod larvae in this study may have been more sensitive than the 11–13 mm larvae to oxidative stress potentially caused by the high-lipid (including LC-PUFA) zooplankton diet. Our microarray results also showed that some redox-relevant genes (e.g. *gpx1b*, *gpx3*) were up-regulated while others (e.g. *peroxiredoxin-1*, *catalase*) were down-regulated in zooplankton-fed cod larvae. Interestingly, similar results were seen in larval Senegal sole (*Solea senegalensis*) fed live *Artemia* nauplii as compared with larvae on inert feeds, where those fed live prey experienced enhanced growth, higher total glutathione peroxidase activity and lipid peroxidation, and lower catalase activity [37]. The ontogeny of *gpx1*, *gpx3*, and *catalase* transcript expression was previously studied by QPCR with templates from cod larvae fed only rotifers and formulated diet (i.e. without zooplankton) [38]. During late larval stages (20–62 dph with a similar temperature regime, and therefore representing a similar period of larval development, as the current study), Hamre et al. [38] found that *catalase* transcript expression was stable whereas *gpx1* and *gpx3* transcripts showed increased expression in successive larval stages. These three transcripts encode enzymes that reduce metabolically generated H_2_O_2_, and therefore play key roles in the control of this reactive oxygen species [39]. H_2_O_2_, which serves as a second messenger in insulin signaling and in the signaling cascades mediated by several growth factors (e.g. EGF, FGF), also plays a significant role in diverse biological processes including proliferation, inflammation, and aging (reviewed by Sies [39]). Collectively, our results suggest that the maintenance of homeostatic oxidative status in 9 mm cod larvae on a high-lipid zooplankton containing diet is complex, involving numerous redox-relevant genes. The RNAseq results of Penglase et al. [27] also show that redox homeostasis is zooplankton-responsive, with some gene expression differences between that study and ours likely due to aforementioned differences in rotifer enrichment methods. Whether or not alterations in H_2_O_2_ signaling play a role in the enhanced growth phenotype of zooplankton-fed cod larvae is currently unknown.

In our microarray study, several genes encoding selenium-dependent antioxidant enzymes (e.g. glutathione peroxidase 1b, glutathione peroxidase 3, thioredoxin reductase 3a, iodothyronine deiodinase type I) and other selenoproteins (e.g. selenoproteins H, L, Ja, and Pa) were up-regulated in zooplankton-fed cod larvae compared with the other larval groups at the 9 mm stage. This suggests that selenium homeostasis was altered in these zooplankton fed larvae. Selenium is an essential micronutrient that is present in copepods at much higher levels than in rotifers (3–5 compared with 0.08–0.09 mg kg^−1^ dry weight, respectively) [16]. Cod larvae fed copepods, likewise, have higher selenium levels than those fed rotifers [14]. Since selenium levels in rotifers are well below the required level for cold-water fish (0.25–0.30 mg kg^−1^ dry weight) [40], it has been suggested that selenium may be the trace mineral most likely to be deficient in this live feed [16], and this has led to studies on the effect of selenium-enriched rotifers on larval cod performance. For example, although they only assessed larval performance metrics until 26–29 dph, (i.e. prior to the period of enhanced growth) larval cod fed rotifers enriched with selenium and iodine (to levels found in copepods) had improved survival [17], and Penglase et al. [41] used QPCR to show that transcripts encoding glutathione peroxidases 1 and 3 were significantly up-regulated in selenium-supplemented cod larvae at 29 dph. In light of these results, the up-regulation of *gpx1b* and *gpx3* transcripts in zooplankton-fed larvae in our study suggests that these cod genes may have responded to the high selenium levels known to be present in zooplankton. As mentioned previously, the lack of a difference in *gpx1a* and *gpx3* expression in ~9 mm larval cod fed rotifers versus copepods in Penglase et al. [27] is likely due to their use of selenium supplemented rotifer enrichment.

Research involving selenium supplementation of larval diets has been conducted with marine fish species in addition to Atlantic cod. For example, Red sea bream (*Pagrus major*) larvae fed selenium-enriched rotifers had higher whole body selenium levels and accelerated growth and development compared with larvae fed a control diet [8]. As well, Betancor et al. [35] used high DHA and vitamin E diets with and without selenium to show that selenium improved larval sea bass growth and prevented lipid oxidation. These data, and previous results from Ribeiro et al. [42], led this group to speculate that selenium supplementation may be more important in earlier life stage larval sole. Both deficient and excess levels of selenium can be detrimental to fish, with selenium deficiency potentially causing reduced growth and glutathione peroxidase activity in plasma and liver [43], and excess selenium causing toxicity [44]. Therefore, an appropriate level of dietary selenium (i.e. meeting the requirement without reaching toxicity) is likely needed for optimal larval cod performance. Benner et al. [45] showed that selenium supplementation in diets for zebrafish (*Danio rerio*) influenced behaviour and increased the brain transcript expression of several selenoprotein-encoding genes including *sepp1a*. In mammals, selenium supply influences the transcript expression of genes encoding some selenoproteins such as glutathione peroxidase 1 and selenoprotein W (reviewed by Hesketh [46]), although selenium status does not appear to influence *selenoprotein P* transcript expression in rat liver (reviewed by Burk and Hill [47]). Still, the known regulation of some selenoprotein-encoding genes by selenium supply supports our hypothesis that dietary selenium was at least partially responsible for the observed up-regulation of selenoprotein-encoding genes in zooplankton-fed cod larvae. In the current study, *selenoprotein Pa* (*sepp1a*) transcript was shown by microarray and QPCR to be over 3-fold up-regulated in zooplankton-fed cod larvae as compared with both other groups. In addition to functioning as an antioxidant and a selenium transporter [48], mammalian selenoprotein P also plays roles in spermatogenesis, immunity, and neurological function (reviewed in [49], whereas suppressed *selenoprotein P* transcript expression is seen in human lung cancer and other malignancies [50]. Consistent with these mammalian data, transcript expression studies in fish suggest that *sepp1a* plays several important roles. For example, Atlantic salmon *sepp1a* transcript was shown by microarray and QPCR to be down-regulated in *Piscirickettsia salmonis* infected head kidney and cultured macrophages [51], suggesting an immune-related function. Xu et al. [52] used QPCR to show that *sepp1a* transcripts were up-regulated in the liver of fast-growing triploid growth hormone transgenic Atlantic salmon. Penglase et al. [27], who as previously noted fed cod larvae with zooplankton versus selenium (and other nutrient) supplemented rotifers, reported that the transcript expression of *sepp1a* was higher in zooplankton-fed larvae at ~ 5 mm stage, but not in 7–25 mm larvae (i.e. during the accelerated growth phase). Our results and those of Penglase et al. [27] collectively suggest that, while *sepp1a* may be a useful biomarker of selenium deficiency, it does not appear to be necessary for zooplankton-associated accelerated growth of larval cod.

Research in mammals suggests that the acute phase response (APR), defined as “a complex systemic early-defense system activated by trauma, infection, stress, neoplasia, and inflammation” [53], inhibits selenoprotein P production (reviewed by Burk and Hill [47]). Interestingly, transcripts encoding positive acute phase proteins (i.e. up-regulated in the APR: e.g. ferritin, ceruloplasmin, and hepcidin [54, 55]) and CCAAT/enhancer-binding protein delta (associated with GO term “acute-phase response”) were identified by microarray in the current study as being significantly up-regulated in both RA and RA-PH larvae compared with RA-Zoo larvae at the 9 mm stage (Additional file 2: Table S2). Therefore, the suppressed *selenoprotein Pa* transcript expression observed in 9 mm RA and RA-PH larvae may be due to an APR that was potentially related to nutritional insufficiency. Since selenium deficiency in humans leads to oxidative stress and inflammation [56], it is possible that selenium deficiency in the RA and RA-PH diets and larvae may have contributed to oxidative stress (as evidenced by responses of several redox related genes) and inflammation/an APR (as evidenced by up-regulation of genes encoding positive acute phase proteins). However, the results of Penglase et al. [27], i.e. differential expression of many redox-relevant genes in cod larvae fed zooplankton versus selenium-replete rotifers, reveal that factors other than selenium are involved in the redox pathway response to dietary zooplankton. The roles and interactions of nutritional status (e.g. dietary selenium, and biomarkers of selenium homeostasis including glutathione peroxidase and selenoprotein P) and APR-associated transcripts and proteins in larval cod growth warrant further investigation.

In addition to the production of selenium-replete rotifers, mineral enrichment trials have been conducted to produce rotifers with copepod levels of selenium, manganese, copper, and zinc [57]. Zooplankton contain higher levels of all of these minerals (as well as iodine) compared with rotifers [16]. Interestingly, 4 genes with copper-relevant functional annotations were identified in this microarray study: 3 down-regulated (*coagulation factor V*, with GO term “copper ion binding”; *catalase*, with GO term “response to copper ion”; and *ceruloplasmin*, with GO terms “copper ion binding”, “response to copper ion”, and “plasma membrane copper ion transport”) and 1 up-regulated (*thioredoxin reductase 3a*, with GO term “cellular response to copper ion”) in zooplankton-fed cod larvae compared with both other larval groups. These genes may have responded to copper deficiency in the rotifer-fed 9 mm larvae in our study. However, since Karlsen et al. [28] used rotifer enrichment methods that resulted in significantly higher levels of copper in rotifers and rotifer-fed larvae compared with zooplankton and zooplankton-fed larvae, respectively, and growth acceleration was still observed in the zooplankton-fed cod, copper in not likely to be a limiting nutrient for growth in larvae of this species.

Studies by our group, using the same larval batches and treatments as those used in the current functional genomics study, showed that accelerated growth in zooplankton-fed cod larvae first appeared in 9–10 mm (30 dph) larvae [18]. This is in agreement with Karlsen et al. [28] who reported that the onset of accelerated growth in copepod-fed cod larvae was between 22 and 36 dph. Since Karlsen et al. [28] used selenium-supplemented rotifers to ensure that the rotifer-fed larvae in their study were not selenium deficient and still saw decreased growth in the absence of copepods, it was proposed that nutrients other than selenium (e.g. taurine) may be limiting for growth in cod larvae fed only rotifers. Collectively, it appears that ~ 9–10 mm in length represents a developmental window in which larval cod growth becomes responsive to nutrients present in the zooplankton supplemented diet (i.e. nutrients that were higher in zooplankton compared with the nutrient-enriched rotifers used in Karlsen et al. [28], such as protein, taurine, iodine, and zinc). Our microarray results and the RNAseq results of Karlsen et al. [28] provide snapshots of the impact of dietary zooplankton on the larval transcriptome at this pivotal developmental stage. However, the results of these studies must be considered in light of the very different rotifer enrichment methods employed.

### Influence of the zooplankton diet on genes involved in thyroid hormone production

Two genes involved in thyroid hormone (TH) production [*iodotyrosine dehalogenase I* (alias *iodotyrosine deiodinase*) and *putative iodothyronine deiodinase type I*] were up-regulated in zooplankton-fed larvae as compared with both other groups (Additional file 1: Table S1). Thyroid hormones, which require iodine for their biosynthesis, play key roles in controlling metamorphosis in larval fish [58], as well as development and metabolism in adult vertebrates [59]. Iodothyronine deiodinases are selenoproteins (i.e. using selenium in the form of selenocysteine in the active site) that catalyze the deiodination of the prohormone thyroxine (T_4_) to the active hormone triiodothyronine (T_3_) [60, 61]. Iodotyrosine deiodinase, which is not a selenoprotein, acts in iodine salvage by catalyzing the deiodination of TH synthesis byproducts [62, 63]. Selenium regulates the expression and activity of selenoproteins including iodothyronine deiodinases and glutathione peroxidases, and selenium treatment has been shown to increase *iodothyronine deiodinase* mRNA levels in chicken and mammalian cells [64, 65]. To our knowledge, it is not known if selenium status affects *iodotyrosine deiodinase* mRNA levels.

Zooplankton contain higher levels of iodine compared with rotifers [16, 28], and in cod larvae of similar age and size to those used in our microarray experiment, Karlsen et al. [28] showed that rotifer-fed larvae at 30 dph (8.5 mm) had significantly lower levels of iodine compared with 28 dph (10.1 mm) growth-accelerated zooplankton-fed larvae. However, Atlantic cod larvae fed rotifers enriched with copepod levels of iodine can experience iodine toxicity [66]. Since zooplankton-fed cod larvae have higher iodine levels than those associated with toxicity in the iodine-enriched rotifer study ([66], 29 vs. 13 mg kg^−1^ dry weight, respectively), others have speculated that nutrient interactions, possibly involving bromine anion (Br^−^, known to be present at high levels in marine organisms), may prevent iodine toxicity in zooplankton-fed larvae [14, 66]. It should be noted that the current study involved the feeding of relatively small amounts of zooplankton (5–10 % of live prey items), which would limit the larval intake of iodine. Still, the potential roles of nutrient interactions in larval cod response to being fed various amounts of zooplankton should be investigated.

### Influence of zooplankton diet on expression of mitosis-relevant genes

Our microarray study identified numerous genes involved in mitosis/cell cycle that responded to dietary zooplankton, and most of these genes were up-regulated in RA-Zoo compared with the other larval groups. Diet-associated growth differences in larval cod have been studied at the histological level, with larvae fed high DHA:EPA ratio rotifers experiencing faster growth (associated with increased muscle hyperplasia, i.e. more muscle fibres) than larvae fed low DHA:EPA ratio rotifers [67]. The diet-associated rapid growth of larval cod by hyperplasia (increased cell number) seen by Galloway et al. [67], and in Katan et al. [18] on the same larval population used in this study, agrees with our results showing dietary zooplankton-associated up-regulation of genes involved in various aspects of cell division including mitotic prometaphase (e.g. *aurora kinase B*; *cyclin B1*; *inner centromere protein*; *cell division cycle protein 20*), mitotic spindle organization (*kinetochore protein Spc25*), and regulation of chromosome segregation (*kinesin-like protein KIF2C*) (Additional file 1: Table S1). Murray et al. [25] used microarrays and QPCR to study the transcript expression responses of larval Atlantic halibut to a microencapsulated diet and showed that larvae fed the microencapsulated diet along with *Artemia* grew significantly slower than larvae fed *Artemia* alone. In the slow-growing halibut larvae, genes involved in cell division (e.g. *mitotic spindle assembly checkpoint protein MAD2A*) were down-regulated while *glutathione S-transferase A1* was up-regulated compared with controls [25]. These results agree with our study, in which slower-growing RA and RA-PH cod larvae experienced lower expression (compared with RA-Zoo) of many genes involved in cell division (including some that encode mitotic spindle components) and higher expression of *glutathione S-transferase*. Three *glutathione S-transferase*-like transcripts were down-regulated in RA-Zoo larvae as compared with those from both other groups (*glutathione S-transferase pi*, *omega class glutathione S-transferase*, and *microsomal glutathione S-transferase*), while no *glutathione S-transferase*-like transcripts were among the genes up-regulated in RA-Zoo compared with both other groups (Additional file 1: Table S1 and Additional file 2: Table S2).

## Conclusion

Numerous studies have previously shown that larval cod that are fed zooplankton experience accelerated growth compared with those that are only fed rotifers. In this study, microarrays and QPCR were used to investigate the larval cod transcriptome response to partial zooplankton supplementation in the diet. Our study complements the recently published work of Karlsen et al. [28] and Penglase et al. [27], and collectively, these studies suggest that altered larval TH synthesis/metabolism and redox homeostasis, as well as taurine and trace mineral (e.g. selenium, iodine) levels in wild zooplankton, are involved in the initiation of accelerated growth experienced by zooplankton-fed cod larvae. However, the precise mechanisms involved and other important details (e.g. optimal levels of zooplankton-associated nutrients in the larval diet; nutrient interactions) remain to be elucidated.

## Methods

### Live feed

Rotifers (*Brachionus plicatilis*) were enriched with Ori-Culture (ORI-GO, Skretting, Vervins, France) (0.25–0.35 g million^−1^ rotifers) for 4 days, and then with Ori-Green (ORI-GO, Skretting) (0.15–0.25 g million^−1^ rotifers) for 2 h prior to being fed to cod larvae. *Artemia* (8 h post-hatch) were enriched with Ori-Culture for 8 h, and then with Ori-Green for 12 h, before being fed to cod larvae. Fish (pollock; *Pollachius virens*) protein hydrolysate (IceProtein Ltd., Iceland) was fed to the rotifers or *Artemia* (0.1 g liter^−1^) for 2 h before the live feeds were offered to cod larvae. Zooplankton were collected from Conception Bay, Newfoundland, and consisted primarily (>90 %) of copepods (*Temora* sp., *Oithona* sp. and *Pseudocalanus* sp.).

### Experimental design and larval rearing

All experiments complied with the guidelines of the Canadian Council on Animal Care, and were approved by the Institutional Animal Care Committee of Memorial University of Newfoundland (protocol number 11-30-KG). The adult Atlantic cod broodstock used herein were wild-caught from Smith Sound (Newfoundland) several years prior to this study. These fish were maintained at the Dr. Joe Brown Aquatic Research Building (JBARB), Memorial University of Newfoundland, St. John’s, Newfoundland, in 37.7 m^3^ tanks supplied with filtered, aerated/oxygenated and UV-treated flow-through seawater at 6.5–7.0 °C. These fish were maintained on a six month advanced photoperiod and fed mackerel and herring with a vitamin supplement twice each week. Eggs for this study were collected from these communally spawning broodstock, disinfected with ozone and distributed into two 0.3 m^3^ cone-shaped incubators with flow-through seawater (32 ppt) at 6.0–7.0 °C until 100 % hatch. The eggs had an average diameter of 1.6 mm, a 95 % fertilization rate and 91 % symmetrical cleavage. At 100 % hatch (93.4 degree days) the larvae were transferred to 16, 400 L flow-through tanks at a density of 50 larvae L^−1^. These tanks were then divided randomly into 3 different treatments based on the following feeding regimes/diets:

**Treatment 1** (6 replicate tanks): Rotifers and *Artemia* (**RA**) 3 feedings per day (9 am, 3 pm and 9 pm), with rotifers fed until the larvae reached ~ 9 mm [26–30 days post-hatch (dph)] and then *Artemia* until they reached ~ 13 mm in length (44–50 dph). This is the standard larval feeding regime used in the JBARB and at many other commercial cod rearing facilities. Initial rotifer and *Artemia* densities during feeding were 800–9000 and 1200–5400 L^−1^ (depending on age of larvae), respectively.

**Treatment 2** (4 replicate tanks): Rotifers and *Artemia* as in Treatment 1, except supplemented with 5–10 % wild caught zooplankton (**RA-Zoo**) from 2 dph until 30 dph, and then *Artemia* alone until they reached ~ 13 mm in length (34 dph, as RA-Zoo grew faster than the other 2 groups). This treatment had four replicates because of limited quantities of zooplankton available for collection at the time of the study. The number of rotifers or *Artemia* fed to each tank was reduced according to the number of zooplankton added (~250,000 per tank per feeding) to maintain consistent numbers of prey items between tanks. During feedings (9 am the first week, and 9 am and 3 pm thereafter), cod larvae were fed only zooplankton for 1 h before the required number of rotifers or *Artemia* was added. Gut squashes were conducted periodically, and confirmed that cod larvae were feeding on the zooplankton.

**Treatment 3** (6 replicate tanks): Rotifers and *Artemia* as in Treatment 1, except with Ori-Green enriched Rotifers/*Artemia* 4 days per week and protein hydrolysate enriched Rotifers/*Artemia* 3 days per week (**RA-PH**); as with the other treatments, rotifers were fed until the larvae reached ~ 9 mm and *Artemia* until they reached ~ 13 mm. This feeding protocol was based on that used in previous experiments conducted by a collaborator, Dr. Rannveig Bjornsdottir (personal communication).

Potter’s clay (400 ml) was added to all the larval rearing tanks, twice a day, to increase tank turbidity and to reduce bacterial numbers within the tanks [68]. Rearing temperature was increased gradually from 6.0–7.0 °C (incubation temperature) to 10.5 °C over a period of 10 days (0–10 dph), and water flow rate was increased gradually from 0.8 L  min^−1^ (at 0 dph) to 4.5 L  min^−1^ at 35 dph. Seawater was passed through sand filters (30 μm), UV-sterilized, degassed, passed through foam fractionation, and oxygenated prior to delivery to the larval tanks. This ensured high water quality and a pathogen-free environment. Dissolved oxygen levels in the tanks were measured daily (YSI, ProODO, OH, USA) and kept at an average value of 117 % saturation to alleviate any possible issues with nitrogen super-saturation.

### Larval sampling and dry weight determination

Larvae were sampled (4–6 per tank) when fish in a particular tank reached an average length of 9–10 mm (referred to as the 9 mm stage), when the RA-Zoo larvae reached ~ 13 mm (at this stage the RA and RA-PH larvae were only ~ 11 mm), and again when the RA and RA-PH larvae reached ~ 13 mm (the latter to provide “size-matched” samples for comparison with RA-Zoo larvae). Six to ten larvae were measured to determine average larval length per tank. The larvae were measured using a dissecting microscope, with length determined as the distance from the tip of the snout to the end of the hypurals using an ocular micrometer. One RA-PH tank was terminated before the 9 mm sampling and one RA-PH tank was terminated before the 11 mm sampling due to high mortality. For the 9 mm stage sampling, all four RA-Zoo tanks and two RA tanks were sampled at 26 dph, three RA and four RA-PH tanks were sampled at 27 dph, and one tank each for RA and RA-PH was sampled at 30 dph. For the age-matched later stage sampling (~13 mm RA-Zoo, ~ 11 mm RA and RA-PH), all RA-Zoo tanks were sampled at 34 dph and all RA and RA-PH tanks were sampled at 35 dph. The ~ 13 mm RA and RA-PH larvae were sampled from 44–50 dph.

The sampled larvae were anaesthetized in MS-222 (tricaine methane-sulphonate; 0.05 g L ^−1^, Syndel Laboratories, BC, Canada). For molecular analysis samples, anaesthetized larvae were rinsed in UV-treated filtered seawater on a mesh strainer, patted dry on a Kimwipe, transferred to RNase/DNase-free 1.5 mL microcentrifuge tubes, flash-frozen in liquid nitrogen and stored at −80 °C until RNA extraction. For dry weight (DW) determination: duplicate samples were collected for each tank (8–12 total samples per treatment), consisting of 10 larvae per sample at 9 mm, and 5 larvae per sample at each 11 mm and 13 mm tank average lengths. For each sample, larvae were counted into a Millipore glass filter holder apparatus (Fisher Scientific) containing a previously dried and weighed Whatman GF/C glass microfiber filter (VWR International), and the larvae were then rinsed down onto the filter with a small volume of seawater under slight vacuum to remove the liquid and finally rinsed with 5–10 mL isotonic (3 %) ammonium formate under slight vacuum. The vacuum dried larvae and filter paper were then transferred to pre-weighed aluminum weigh boats and dried at 80 °C for a minimum of 24 h before being weighed on an analytical balance (Denver Instrument APX-60, Arvada, Co, USA).

The dry mass data was analysed by one-way ANOVA after Shapiro-Wilk normality and an equal variance test were performed, and a Holm-Sidak pairwise multiple comparison was used to identify significant differences between groups. Where the normality or equal variance tests failed, a Kruskal-Wallis one-way analysis of variance on ranks was performed followed by a Dunn’s pairwise multiple comparison. Dunn’s pairwise multiple comparison was used for the dry mass data set due to unequal treatment group sizes. In all, the following comparisons were performed: 1) treatment groups (RA; RA-PH; RA-Zoo) within a size-matched category (9 mm; 11 mm; 13 mm); 2) treatment groups (RA; RA-PH; RA-Zoo) within an age-matched category (34–35 dph); and 3) sizes (9 mm; 11 mm; 13 mm) within a treatment category (RA; RA-PH; RA-Zoo). All statistical analyses were performed using Sigmaplot Version 2.0 (Systat Software Inc., San Jose, CA, USA), and *p* < 0.05 was used as the level of statistical significance.

### RNA extraction, DNAse treatment, and purification

Larval samples were homogenized in 300 μL of TRIzol Reagent (Invitrogen/Life Technologies) using a motorized Kontes RNase-Free Pellet Pestle Grinder (Kimble Chase, Vineland, NJ). An additional 450 μL of TRIzol Reagent (Invitrogen/Life Technologies) was added, mixed by pipetting, and the homogenates frozen on dry ice and stored at −80 °C. Frozen homogenates were further processed by slowly thawing on ice and then passing through a QIAshredder (QIAGEN, Mississauga, ON) spin column following the manufacturer’s instructions. Two-hundred and fifty μL of TRIzol (Invitrogen/Life Technologies) was then added to each sample to make a total homogenate volume of approximately 1 mL, and the TRIzol total RNA extractions were then completed following the manufacturer’s instructions.

Individual total RNA samples were treated with 6.8 Kunitz units of DNaseI (RNase-Free DNase Set, QIAGEN) with the manufacturer’s buffer (1× final concentration) at room temperature for 10 min to degrade any residual genomic DNA. DNase-treated RNA samples were column-purified using the RNeasy MinElute Cleanup Kit (QIAGEN) following the manufacturer’s methods. RNA integrity was verified by 1 % agarose gel electrophoresis, and RNA purity was assessed by A260/280 and A260/230 NanoDrop UV spectrophotometry for both the pre-cleaned and the column-purified RNA samples. Column-purified RNA samples had A260/280 ratios between 2.0 and 2.2 and A260/230 ratios between 2.0 and 2.4.

### Microarray hybridization and data acquisition

Individual DNAse-treated, column-purified total RNA samples from 8 larvae per group (9 mm stage for each RA, RA-PH, and RA-Zoo larvae; 2 larvae from each of 4 replicate tanks) were used for microarray analysis using the Atlantic cod 20K oligonucleotide microarray platform [19] and a common reference experimental design. On each array, RNA from an individual larva was hybridized together with a common reference RNA.

For each larva, 1 μg of RNA was amplified using the Amino Allyl MessageAmp™ II aRNA Amplification Kit (Ambion/Life Technologies) according to the manufacturer's protocol. To generate the common reference pool, 10 μg of aRNA from each of the twenty-four 9 mm individuals was pooled. Twenty μg of each individual sample aRNA was labeled with Cy5, and 20 μg aliquots of the common reference aRNA were labeled with Cy3 (CyDye Post-Labeling Reactive Dye Packs, GE Healthcare Life Sciences) using the Amino Allyl MessageAmp™ II aRNA Amplification Kit (Ambion/Life Technologies) according to the manufacturer's protocol.

For each array, 3 μg Cy5-labeled aRNA from an individual larva was combined with 3 μg Cy3-labeled aRNA from the common reference. Labeled aRNA was then fragmented using Ambion Fragmentation Reagents (Ambion). Fragmented aRNA and 2 μL of LNA blocker (Genisphere, Hatfield, Pennsylvania, USA) were combined in formamide-based hybridization buffer (Genisphere), applied to the array and hybridized overnight (∼16 h) at 42 °C. Detailed protocols including prehybridization and washing are available in Booman et al. [19].

Hybridized arrays were scanned at 5 μm resolution using a ScanArray Gx Plus scanner and ScanExpress v4.0 (Perkin Elmer, Waltham, Massachusetts, USA), and signal intensity data was extracted using Imagene 9.0 (BioDiscovery, El Segundo, California, USA). Using R and the Bioconductor package marray, control spots and Imagene-flagged spots were removed and data was log_2_-transformed and Loess-normalized per subgrid. Spots with signal values below a threshold of median background + 3× SD were removed. Duplicate spots were averaged to obtain a normalized, thresholded and averaged dataset of 20,000 probes. Finally, probes that were absent in more than 25 % of all arrays were completely removed from the dataset, resulting in a final dataset of 15,740 probes and 24 arrays. Detailed protocols for these procedures are available in Booman et al. [19]. This microarray experiment was performed according to MIAME guidelines [69]. The full microarray dataset is submitted to NCBI’s Gene Expression Omnibus (GEO) repository with series accession number GSE68792.

### Microarray data analysis

Two-class comparison analysis was performed using Significance Analysis of Microarrays (SAM) as implemented in the Bioconductor package siggenes. Missing values were imputed using the EM_array method from LSimpute and a false discovery rate (FDR) cutoff of 0.05 was used to determine significant gene expression differences. Blast2GO [70] was used for automated functional annotation of the gene list containing 303 overlapping microarray features (i.e. differentially expressed between RA-Zoo and both RA and RA-PH) with protein names (Blastx against nr database, E-value < 1.00E-05) and Gene Ontology (GO) terms, based on the expressed sequence tags (ESTs) or contiguous sequences (contigs) from which the oligonucleotide probes were designed. GO enrichment analysis of this differentially expressed gene list was performed using Fisher’s Exact test with an FDR cutoff of 0.05, using the 20K cod array as the reference set. Hierarchical clustering and heatmaps were constructed in Genesis [71]; all data were median-centered and clustered using Pearson correlation and complete linkage hierarchical clustering as in Booman et al. [21].

### Real-time quantitative polymerase chain reaction (QPCR)

First-strand cDNA templates for QPCR were synthesized in 20 μL reactions from 1 μg of DNaseI-treated, column-purified total RNA using random primers (250 ng; Invitrogen/Life Technologies) and M-MLV reverse transcriptase (200 U; Invitrogen/Life Technologies) with the manufacturer’s first strand buffer (1× final concentration) and DTT (10 mM final concentration) at 37 °C for 50 min.

QPCR analyses of transcript (mRNA) expression levels were performed using SYBR Green I dye chemistry and the 7500 Fast Real Time PCR system (Applied Biosystems/Life Technologies). Reaction volume for the PCR amplifications was 13 μL and contained 1× Power SYBR Green PCR Master Mix (Applied Biosystems/Life Technologies), 50 nM of both the forward and reverse primers, and the indicated cDNA quantity (see below). The real-time analysis program consisted of 1 cycle of 50 °C for 2 min, 1 cycle of 95 °C for 10 min, and 40 cycles of 95 °C for 15 s and 60 °C for 1 min, with fluorescence detection at the end of each 60 °C step.

The sequences of all primer pairs used in QPCR analyses are presented in Table 2. Each primer pair was quality tested to ensure that a single product was amplified (dissociation curve analysis) and that there was no primer-dimer present in the no-template control. Amplicons were electrophoretically separated on 2 % agarose gels and compared with a 1 kb plus ladder (Invitrogen/Life Technologies) to verify that the correct size fragment was being amplified. Amplification efficiencies [72] were calculated using cDNA synthesized from a 9 mm RA and from a 9 mm RA-Zoo RNA sample for the target genes [i.e. transcripts of interest (TOI)] identified as differentially expressed in the 9 mm stage microarray analyses, and from a 9 mm RA and a 13 mm RA RNA sample for the candidate normalizer genes. The reported efficiencies (Table 2) are an average of the two values. Standard curves were generated using a 5-point 1:3 dilution series starting with cDNA representing 10 ng of input total RNA.

**Table 2:** Primers used in QPCR studies

Gene name	Primer name	Nucleotide sequence (5′-3′)	Efficiency (%)^a^	Amplicon size (bp)
*coagulation factor V*	36536-1F	GGGAACACGGATAACAATGG	103.4	132
	36536-1R	AAAGTCACAGCCGAGCAACT		
*dehydrogenase/reductase SDR family member 1*	36829-1F	GTCTTGATGACCTGCGACCT	85.0	110
	36829-1R	AGGTAAGGGACCTGGGACAC		
*glutathione peroxidase 1b*	37395-2F	TGCTTCAGAAGTCGGATGTG	101.6	135
	37395-2R	AGACTGGGCTCCAGATGATG		
*cytochrome P450 CYP2Y3*	37900-2F	CAGGAACGGAGACAACCAGT	105.0	109
	37900-2R	ACCCAATGACAGAGGCAATC		
*microsomal glutathione S-transferase 3*	38166-1F	TGATCGCCATGGATATGCTA	104.0	113
	38166-1R	TGCACCCCCAACTTTTACTC		
*peroxiredoxin-1* (alias *natural killer enhancing factor*)	39283-1F	GTGCTTTCAGAGGGCTGTTC	100.7	146
	39283-1R	GCACACTTCCCCGTATTTGT		
*DNA-damage-inducible transcript 4* (alias *REDD1*)	41986-1F	ACTGCTCACGTCACAAGGTG	99.7	118
	41986-1R	GTTCCTAACGACGCTTCTGC		
*selenoprotein Pa*	42046-1F	GGGCAGGGTCATGTAGAGAA	101.5	116
	42046-1R	CTGGTCACTACTGGCCTGGT		
*solute carrier family 6, member 6*	42481-2F	GTCTGAGGGACCTCTGATCG	101.4	102
	42481-2R	AGCTCGCTGTCCTTCACAAT		
*thioredoxin-interacting protein*	42540-2F	GGCGATGACGAAGTGTGTAA	91.7	111
	42540-2R	TGGCTCACCTCCACGATTAT		
*trypsinogen H1_3a1*	42811-2F	GACGCTGGACTACGACATCA	81.4	124
	42811-2R	TCCAGACACGACACACTGGT		
*aurora kinase B*	42084-2F	AGCCACTCGGAGACGTACAC	97.6	89
	42084-2R	TTGGAGATCAGGTCCTTTGC		
*nattectin precursor*	46230-1F	GGCATTGAGGCAGATAGAGG	98.9	150
	46230-1R	TCAGACTGTGGGTCTTGCAG		
*ferritin, middle subunit*	37201-1F	TGGCTTGGATTCCATAAAGC	107.7	102
	37201-1R	GGTCAAAAGTGCTCCGTCAT		
*RNA polymerase II elongation factor ELL2*	41953-2F	GCTTCCGCATAAAGACAAGG	93.8	150
	41953-2R	GGATAACAGCGGCGTGTACT		

^a^Amplification efficiencies were calculated using a 5-point 1:3 dilution series starting with cDNA representing 10 ng of input RNA. See Methods for additional details

To select the normalizer gene for this study, QPCR primers pairs were designed for five candidate normalizers [*60S ribosomal protein L34*; *ATPase, H+ transporting, lysosomal, V1 subunit H*; *proteasome 26S subunit, non-ATPase, 12*; *RNA polymerase II elongation factor ELL2*; *transcription elongation factor A (SII)*] that exhibited a stable expression profile in the microarray studies. The primer pairs were quality tested as described above. Furthermore, the fluorescence threshold cycle (C_T_) values of 24 samples representing the 8 different experimental groups [3 from the each of the RA and RA-PH (9, 11 and 13 mm) groups and RA-Zoo (9 and 13 mm) groups] were measured using cDNA representing 5 ng of input total RNA, and then analyzed using geNorm. *RNA polymerase II elongation factor ELL2* was chosen as the normalizer gene for this QPCR study as it had the best combination of amplification efficiency (92 %) and stability (geNorm M = 0.289).

Transcript levels were measured in 8 larvae (2 larvae from each of 4 replicate tanks) for each of the 8 groups. In all cases, cDNA representing 5 ng of input RNA was used as template in the PCR reactions. On each plate, for every sample, the target gene and endogenous control were tested in triplicate, and a plate linker sample (i.e. a sample that was run on all plates in a given study) and a no-template control were included. The C_T_ values were determined using the 7500 Software Relative Quantification Study Application (Version 2.0) (Applied Biosystems/Life Technologies). The relative quantity (RQ) of each transcript was determined using the Pfaffl method [72], with amplification efficiencies (Table 2) incorporated. For each TOI, the sample with the lowest normalized expression (mRNA) level was set as the calibrator sample (i.e. assigned an RQ value = 1); transcript expression data are presented as RQ normalized to *RNA polymerase II elongation factor ELL2*.

The QPCR (RQ) data were analysed using identical statistical procedures as described for the dry mass data, except that Kruskal-Wallis one-way analysis of variance on ranks was followed by a Tukey’s pairwise multiple comparison. *P* values < 0.05 were considered to be significant.

### Availability of supporting data

The full microarray dataset has been submitted to NCBI’s Gene Expression Omnibus (GEO) with the series accession number GSE68792. The link to this GEO archived dataset follows: http://www.ncbi.nlm.nih.gov/geo/query/acc.cgi?acc=GSE68792. Additional supporting data are included as additional files (Additional file 1: Table S1, Additional file 2: Table S3 and Additional file 3: Table S3).

## Additional files

Additional file 1: Table S1. Microarray-identified genes significantly up-regulated in RA-Zoo compared with both RA and RA-PH. (PDF 350kb) Additional file 2: Table S2. Microarray-identified genes significantly down-regulated in RA-Zoo compared with both RA and RA-PH. (PDF 310kb) Additional file 3: Table S3. Complete list of overrepresented and underrepresented GO terms in test set of genes (303 genes differentially expressed between RA-Zoo and both RA and RA-PH) compared with reference set of genes (20K cod microarray). (PDF 90kb)

## Abbreviations

APR: acute phase response
DE: differentially expressed
DHA: docosahexaenoic acid
dph: days post-hatch
EPA: eicosapentaenoic acid
FDR: false discovery rate
GEO: gene expression omnibus
GO: gene ontology
LC-PUFA: long-chain polyunsaturated fatty acids
QPCR: real-time quantitative polymerase chain reaction
RA: rotifer treatment group
RA-PH: rotifers with protein hydrolysate treatment group
RA-Zoo: rotifers with zooplankton treatment group
RNAseq: RNA sequencing
RQ: relative quantity
SAM: Significance Analysis of Microarrays
SSH: suppression subtractive hybridization
TH: thyroid hormone

## References

[1] Kjesbu OS, Taranger GL, Trippel EA (2006). Gadoid mariculture: development and future challenges. ICES J Mar Sci.

[2] Rosenlund G, Skretting M (2006). Worldwide status and perspective on gadoid culture. ICES J Mar Sci.

[3] Johansen SD, Coucheron DH, Andreassen M, Karlsen BO, Furmanek T, Jørgensen TE (2009). Large-scale sequence analyses of Atlantic cod. N Biotechnol.

[4] Bowman S, Hubert S, Higgins B, Stone C, Kimball J, Borza T, Bussey JT, Simpson G, Hall JR, Hori TS, Feng CY, Gamperl AK, Booman M, Rise M, Symonds J, Johnson SC, Rise ML (2011). An integrated approach to gene discovery and marker development in Atlantic cod (*Gadus morhua*). Mar Biotechnol.

[5] Beaugrand G, Brander KM, Lindley JA, Souissi S, Reid PC (2003). Plankton effect on cod recruitment in the North Sea. Nature.

[6] Kristiansen T, Stock C, Drinkwater KF, Curchitser EN (2014). Mechanistic insights into the effects of climate change on larval cod. Glob Change Biol.

[7] Rosenlund G, Halldórsson Ó (2007). Cod juvenile production: Research and commercial developments. Aquaculture.

[8] Kim H, Sakakura Y, Maruyama I, Nakamura T, Takiyama K, Fujiki H, Hagiwara A (2014). Feeding effect of selenium enriched rotifers on larval growth and development in red sea bream *Pagrus major*. Aquaculture.

[9] Beaugrand G, Kirby RR (2010). Climate, plankton and cod. Glob Change Biol.

[10] Nicholas D, Rochette S, Llope M, Licandro P (2014). Spatio-temporal variability of the North Sea cod recruitment in relation to temperature and zooplankton. PLoS One.

[11] Imsland AK, Foss A, Koedijk R, Folkvord A, Stefansson SO, Jonassen TM (2006). Short- and long-term differences in growth, feed conversion efficiency and deformities in juvenile Atlantic cod (*Gadus morhua*) startfed on rotifers of zooplankton. Aquac Res.

[12] Koedijk RM, Folkvord A, Foss A, Pittman K, Stefansson SO, Handeland S, Imsland AK (2010). The influence of first-feeding diet on the Atlantic cod *Gadus morhua* phenotype: survival, development and long-term consequences for growth. J Fish Biol.

[13] Koedijk RM, Le François NR, Blier PU, Foss A, Folkvord A, Ditlecadet D (2010). Ontogenetic effects of diet during early development on growth performance, myosin mRNA expression and metabolic enzyme activity in Atlantic cod juveniles reared at different salinities. Comp Biochem Physiol A Comp Physiol.

[14] Busch KET, Falk-Petersen I, Peruzzi S, Rist NA, Hamre K (2010). Natural zooplankton as larval feed in intensive rearing systems for juvenile production of Atlantic cod (*Gadus morhua* L.). Aquac Res.

[15] van der Meeren T, Olsen RE, Hamre K, Fyhn HJ (2008). Biochemical composition of copepods for evaluation of feed quality in production of juvenile marine fish. Aquaculture.

[16] Hamre K, Srivastava A, Rønnestad I, Mangor-Jensen A, Stoss J (2008). Several micronutrients in the rotifer *Brachionus* sp. may not fulfil the nutritional requirements of marine fish larvae. Aquacult Nutr.

[17] Hamre K, Mollan TA, Sæle Ø, Erstad B (2008). Rotifers enriched with iodine and selenium increase survival in Atlantic cod (*Gadus morhua*) larvae. Aquaculture.

[18] Katan T, Nash GW, Rise ML, Hall JR, Fernandes JMO, Boyce D, Johnsen CA, Gamperl AK (2016). A little goes a long way: improved growth in Atlantic cod (*Gadus morhua*) fed small amounts of wild zooplankton. Aquaculture.

[19] Booman M, Borza T, Hori TS, Feng CY, Higgins B, Culf A, Leger D, Chute I, Hall JR, Belkaid A, Rise M, Gamperl AK, Hubert S, Kimball J, Ouelette R, Johnson SC, Bowman S, Rise ML (2011). Development and experimental validation of a 20K Atlantic cod (*Gadus morhua*) oligonucleotide microarray based on a collection of over 150,000 ESTs. Mar Biotechnol.

[20] Star B, Nederbragt AJ, Jentoft S, Grimholt U, Malmstrøm M, Gregers TF (2011). The genome sequence of Atlantic cod reveals a unique immune system. Nature.

[21] Booman M, Xu Q, Rise ML (2014). Evaluation of the impact of camelina oil-containing diets on the expression of genes involved in the innate anti-viral immune response in Atlantic cod (*Gadus morhua*). Fish Shellfish Immunol.

[22] Hori TS, Gamperl AK, Booman M, Nash GW, Rise ML (2012). A moderate increase in ambient temperature modulates the Atlantic cod (*Gadus morhua*) spleen transcriptome response to intraperitoneal viral mimic injection. BMC Genomics.

[23] Hori TS, Gamperl AK, Nash GW, Booman M, Barat A, Rise ML (2013). The impact of a moderate chronic temperature increase on spleen immune-relevant gene transcription depends on whether Atlantic cod are stimulated with bacterial versus viral antigens. Genome.

[24] Rise ML, Nash GW, Hall JR, Booman M, Hori TS, Trippel EA, Gamperl AK (2014). Variation in embryonic mortality and maternal transcript expression among Atlantic cod (*Gadus morhua*) broodstock: a functional genomics study. Mar Genomics.

[25] Murray HM, Lall SP, Rajaselvam R, Boutilier LA, Flight RM, Blanchard B (2010). Effect of early introduction of microencapsulated diet to larval Atlantic halibut, *Hippoglossus hippoglossus* L. assessed by microarray analysis. Mar Biotechnol.

[26] Ferraresso S, Bonaldo A, Parma L, Cinotti S, Massi P, Bargelloni L, Gatta PP (2013). Exploring the larval transcriptome of the common sole (*Solea solea* L.). BMC Genomics.

[27] Penglase S, Edvardsen RB, Furmanek T, Rønnestad I, Karlsen Ø, van der Meeren T, Hamre K (2015). Diet affects the redox system in developing Atlantic cod (*Gadus morhua*) larvae. Redox Biology.

[28] Karlsen Ø, van der Meeren T, Rønnestad I, Mangor-Jensen A, Galloway TF, Kjørsvik E, Hamre K (2015). Copepods enhance nutritional status, growth and development in Atlantic cod (*Gadus morhua* L.) larvae – can we identify the underlying factors?. PeerJ.

[29] Hamre K, Yúfera M, Rønnestad I, Boglione C, Conceição LEC, Izquierdo M (2013). Fish larval nutrition and feed formulation: knowledge gaps and bottlenecks for advances in larval rearing. Rev Aquac.

[30] Sahlmann C, Sutherland BJG, Kortner TM, Koop BF, Krogdahl Å, Bakke AM (2013). Early response of gene expression in the distal intestine of Atlantic salmon (*Salmo salar* L.) during the development of soybean meal induced enteritis. Fish Shellfish Immunol.

[31] Higuchi M, Miura C, Iwai T, Miura T (2013). Trypsin regulates meiotic initiation in the Japanese eel (*Anguilla japonica*) by promoting the uptake of taurine into germ cells during spermatogenesis. Biol Reprod.

[32] Klungsøyr J, Tilseth S, Wilhelmsen S, Falk-Petersen S, Sargent JR (1989). Fatty acid composition as an indicator of food intake in cod larvae *Gadus morhua* from Lofoten. Northern Norway Mar Biol.

[33] McEvoy LA, Naess T, Bell JG, Lie Ø (1998). Lipid and fatty acid composition of normal and malpigmented Atlantic halibut (*Hippoglossus hippoglossus*) fed enriched *Artemia*: a comparison with fry fed wild copepods. Aquaculture.

[34] Evjemo JO, Reitan KI, Olsen Y (2003). Copepods as live food organisms in the larval rearing of halibut larvae (*Hippoglossus hippoglossus* L.) with special emphasis on the nutritional value. Aquaculture.

[35] Betancor MB, Caballero MJ, Terova G, Saleh R, Atalah E, Benítez-Santana T, Bell JG, Izquierdo M (2012). Selenium inclusion decreases oxidative stress indicators and muscle injuries in sea bass larvae fed high-DHA microdiets. Br J Nutr.

[36] Kalaimani N, Chakravarthy N, Shanmugham R, Thirunavukkarasu AR, Alavandi SV, Santiago TC (2008). Anti-oxidant status in embryonic, post-hatch and larval stages of Asian seabass (*Lates calcarifer*). Fish Physiol Biochem.

[37] Fernández-Díaz C, Kopecka J, Cañavate JP, Sarasquete C, Solé M (2006). Variations on development and stress defences in *Solea senegalensis* larvae fed on live and microencapsulated diets. Aquaculture.

[38] Hamre K, Penglase SJ, Rasinger JD, Skjærven KH, Olsvik PA (2014). Ontogeny of redox regulation in Atlantic cod (*Gadus morhua*) larvae. Free Radical Bio Med.

[39] Sies H (2014). Role of metabolic H_2_O_2_ generation: redox signaling and oxidative stress. J Biol Chem.

[40] National Research Council (NRC) (1993). Nutrient Requirements of Fish.

[41] Penglase S, Nordgreen A, van der Meeren T, Olsvik PA, Sæle Ø, Sweetman JW (2010). Increasing the level of selenium in rotifers (*Brachionus plicatilis* ‘Cayman’) enhances the mRNA expression and activity of glutathione peroxidase in cod (*Gadus morhua* L.) larvae. Aquaculture.

[42] Ribeiro ARA, Ribeiro L, Sæle Ø, Dinis MT, Moren M (2012). Iodine and selenium supplementation increased survival and changed thyroid hormone status in Senegalese sole (*Solea senegalensis*) larvae reared in a recirculation system. Fish Physiol Biochem.

[43] Wang C, Lovell RT (1997). Organic selenium sources, selenomethionine and selenoyeast, have higher bioavailability than an inorganic selenium source, sodium selenite, in diets for channel catfish (*Ictalurus punctatus*). Aquaculture.

[44] Hamilton SJ, Holley KM, Buhl KJ, Bullard FA (2005). Selenium impacts on razorback sucker, Colorado: Colorado River III. Larvae. Ecotox Environ Safe.

[45] Benner MJ, Drew RE, Hardy RW, Robison BD (2010). Zebrafish (*Danio rerio*) vary by strain and sex in their behavioral and transcriptional responses to selenium supplementation. Comp Biochem Physiol A Mol Integr Physiol.

[46] Hesketh J (2008). Nutrigenomics and selenium: gene expression patterns, physiological targets, and genetics. Annu Rev Nutr.

[47] Burk RF, Hill KE (2005). Selenoprotein P: an extracellular protein with unique physical characteristics and a role in selenium homeostasis. Annu Rev Nutr.

[48] Burk RF, Hill KE, Motley AK (2003). Selenoprotein metabolism and function: evidence for more than one function for selenoprotein P. J Nutr.

[49] Burk RF, Hill KE (2009). Selenoprotein P – expression, functions, and roles in mammals. Biochim Biophys Acta.

[50] Gresner P, Gromadzinska J, Jablonska E, Kaczmarski J, Wasowicz W (2009). Expression of selenoprotein-coding genes *SEPP1*, *SEP15* and *hGPX1* in non-small cell lung cancer. Lung Cancer.

[51] Rise ML, Jones SRM, Brown GD, von Schalburg KR, Davidson WS, Koop BF (2004). Microarray analyses identify molecular biomarkers of Atlantic salmon macrophage and hematopoietic kidney response to *Piscirickettsia salmonis* infection. Physiol Genomics.

[52] Xu Q, Feng CY, Hori TS, Plouffe DA, Buchanan JT, Rise ML (2013). Family-specific differences in growth rate and hepatic gene expression in juvenile triploid growth hormone (GH) transgenic Atlantic salmon (*Salmo salar*). Comp Biochem Physiol D Genomics Proteomics.

[53] Cray C, Zaias J, Altman NH (2009). Acute phase response in animals: a review. Comparative Med.

[54] Bayne CJ, Gerwick L (2001). The acute phase response and innate immunity of fish. Dev Comp Immunol.

[55] Lee J, Pooley NJ, Mohd-Adnan A, Martin SAM (2014). Cloning and characterisation of multiple ferritin isoforms in the Atlantic salmon (*Salmo salar*). PLoS One.

[56] Salehi M, Sohrabi Z, Ekramzadeh M, Fallahzadeh MK, Ayatollahi M, Geramizadeh B (2013). Selenium supplementation improves the nutritional status of hemodialysis patients: a randomized, double-blind, placebo-controlled trial. Nephrol Dial Transpl.

[57] Nordgreen A, Penglase S, Hamre K (2013). Increasing the levels of the essential trace elements Se, Zn, Cu and Mn in rotifers (*Brachionus plicatilis*) used as live feed. Aquaculture.

[58] Hamre K (2006). Nutrition in cod (*Gadus morhua*) larvae and juveniles. ICES J Mar Sci.

[59] Campinho MA, Galay-Burgos M, Sweeney GE, Power DM (2010). Coordination of deiodinase and thyroid hormone receptor expression during the larval to juvenile transition in sea bream (*Sparus aurata*, Linnaeus). Gen Comp Endocrinol.

[60] Orozco A, Valverde-R C (2005). Thyroid hormone deiodination in fish. Thyroid.

[61] Lu J, Holmgren A (2009). Selenoproteins. J Biol Chem.

[62] Rokita SE, Adler JM, McTamney PM, Watson JA (2010). Efficient use and recycling of the micronutrient iodide in mammals. Biochimie.

[63] Phatarphekar A, Buss JM, Rokita SE (2014). Iodotyrosine deiodinase: a unique flavoprotein present in organisms of diverse phyla. Mol BioSyst.

[64] Wu X, Wei C, Pan C, Duan Y, Huang K (2010). Regulation of expression and activity of selenoenzymes by different forms and concentrations of selenium in primary cultured chicken hepatocytes. Br J Nutr.

[65] Foroughi MA, Dehghani H, Mahdavi-Shahri N, Bassami MR (2013). Sodium selenite increases the transcript levels of iodothyronine deiodinases I and II in ovine and bovine fetal thyrocytes *in vitro*. J Trace Elem Med Biol.

[66] Penglase S, Harboe T, Sæle Ø, Helland S, Nordgreen A, Hamre K (2013). Iodine nutrition and toxicity in Atlantic cod (*Gadus morhua*) larvae. PeerJ.

[67] Galloway TF, Kjørsvik E, Kryvi H (1999). Muscle growth and development in Atlantic cod larvae (*Gadus morhua L.*) related to different somatic growth rates. J Exp Biol.

[68] Prickett, R, Boyce D, Monk J. Marine finfish hatchery in Canada uses clay to rear cod. St. John's, NL, Canada: The Cold Harvester, Newfoundland Aquaculture Spring Edition 2010;18–19.

[69] Brazma A, Hingamp P, Quackenbush J, Sherlock G, Spellman P, Stoeckert C (2001). Minimum information about a microarray experiment (MIAME) - toward standards for microarray data. Nat Genet.

[70] Conesa A, Gotz S, Garcia-Gomez JM, Terol J, Talon M, Robles M (2005). Blast2GO: a universal tool for annotation, visualization and analysis in functional genomics research. Bioinformatics.

[71] Sturn A, Quackenbush J, Trajanoski Z (2002). Genesis: cluster analysis of microarray data. Bioinformatics.

[72] Pfaffl MW (2001). A new mathematical model for relative quantification in real-time RT-PCR. Nucleic Acids Res.

