# Supplementary material for: Transcriptome profiling reveals that feeding wild zooplankton to larval Atlantic cod (Gadus morhua) influences suites of genes involved in oxidation-reduction, mitosis, and selenium homeostasis
Source: BMC Genomics. 2015 Nov 26;16:1016. doi: 10.1186/s12864-015-2120-1 (PMC4661974; doi:10.1186/s12864-015-2120-1)
Supplement: Additional file 1: Table S1. — Microarray-identified genes significantly up-regulated in RA-Zoo compared with both RA and RA-PH. (PDF 350kb) [file 12864_2015_2120_MOESM1_ESM.pdf]

Supplemental Table 1. Microarray-identified genes significantly up-regulated in RA-Zoo compared with both RA and RA-PH.

Probe identifier[1]	BLASTx identification			GO terms[3]			Microarray fold change in RA-Zoo versus:	
	**Best named BLASTx hit[2]**	**Accession #**	**E-Value**	**BP**	**MF**	**CC**	**RA**	**RA-PH**
42811	Trypsinogen H1_3a1 [*Dissostichus mawsoni*]	AEA08590	2.07E-120	proteolysis	serine-type endopeptidase activity	extracellular region	5.62	3.40
48948	No significant blastx hit			N/A			5.57	6.09
46089	Proline-serine-threonine phosphatase-interacting protein 2 [*Salmo salar*]	ACN10365	6.62E-161	cytokinesis	actin binding	actomyosin contractile ring	4.16	3.71
				cytoskeleton organization	protein phosphatase binding	cleavage furrow		
				-	-	stress fiber		
				-	-	membrane		
				-	-	cytosol		
54507	No significant blastx hit			N/A			3.42	2.35
42046	Selenoprotein Pa [*Oncorhynchus mykiss*]	CCX35038	1.97E-54	response to selenium ion	selenium binding	extracellular space	3.31	3.89
				selenium compound metabolic process	-	-		
				response to oxidative stress	-	-		
				post-embryonic development	-	-		
				brain development	-	-		
				growth	-	-		
				sexual reproduction	-	-		
				locomotory behavior	-	-		
46285	Vertebrate MID1 interacting-like protein [*Perca flavescens*]	ABW06867	2.17E-40	regulation of lipid biosynthetic process	protein binding	intracellular organelle part	3.24	2.46
				regulation of cellular biosynthetic process	-	intracellular non-membrane-bounded organelle		
				-	-	nucleus		
45626	No significant blastx hit			N/A			3.04	2.02
46912	Unnamed protein product [*Oncorhynchus mykiss*]	CDQ57913	2.07E-82	activation of signaling protein activity involved in unfolded protein response	-	endoplasmic reticulum lumen	2.99	2.77
50644	No significant blastx hit			N/A			2.75	2.84
39070	Nicotinamide riboside kinase 2 [*Esox lucius*]	ACO13872	1.69E-102	pyridine nucleotide biosynthetic process	protein binding	intracellular	2.70	2.67
				integrin-mediated signaling pathway	nucleotide binding	-		
				negative regulation of myoblast differentiation	kinase activity	-		
				cell-substrate adherens junction assembly	-	-		
				phosphorylation	-	-		
				basement membrane assembly	-	-		
37395	Glutathione peroxidase 1b [*Sparus aurata*]	AFY97791	1.81E-101	hydrogen peroxide catabolic process	glutathione binding	cytosol	2.57	2.68
				triglyceride metabolic process	SH3 domain binding	mitochondrion		
				response to symbiotic bacterium	glutathione peroxidase activity	nucleus		
				intrinsic apoptotic signaling pathway in response to oxidative stress	phospholipid-hydroperoxide glutathione peroxidase activity	-		
				response to gamma radiation	selenium binding	-		
				skeletal muscle tissue regeneration	endopeptidase inhibitor activity	-		
				response to selenium ion	-	-		
				temperature homeostasis	-	-		
				purine nucleotide catabolic process	-	-		
				endothelial cell development	-	-		
				sensory perception of sound	-	-		

				vasodilation	-	-		
				glutathione metabolic process	-	-		
				myotube differentiation	-	-		
				protein oxidation	-	-		
				angiogenesis involved in wound healing	-	-		
				blood vessel endothelial cell migration	-	-		
				UV protection	-	-		
				regulation of mammary gland epithelial cell proliferation	-	-		
				fat cell differentiation	-	-		
				myoblast proliferation	-	-		
				negative regulation of inflammatory response to antigenic stimulus	-	-		
				skeletal muscle fiber development	-	-		
				regulation of neuron apoptotic process	-	-		
				interaction with symbiont	-	-		
				negative regulation of cysteine-type endopeptidase activity involved in apoptotic process	-	-		
				release of cytochrome c from mitochondria	-	-		
				cell redox homeostasis	-	-		
				positive regulation of protein kinase B signaling cascade	-	-		
				regulation of proteasomal protein catabolic process	-	-		
				response to lipid hydroperoxide	-	-		
				heart contraction	-	-		
				response to toxic substance	-	-		
				purine nucleobase metabolic process	-	-		
				negative regulation of apoptotic process	-	-		
				response to xenobiotic stimulus	-	-		
				apoptotic process	-	-		
				regulation of gene expression, epigenetic	-	-		
44254	Selenoprotein L [*Salmo salar*]	NP_001180383	8.76E-100	N/A			2.52	2.11
36623	Complement component C9 [*Xiphophorus hellerii*]	AEJ08068	7.76E-09	response to stimulus	-	-	2.48	2.68
48737	No significant blastx hit			N/A			2.36	2.67
38178	Mucin 2 [*Sparus aurata*]	AFP97545	4.84E-81	negative regulation of cell migration	protein dimerization activity	proteinaceous extracellular matrix	2.24	1.94
				apoptotic process	-	cytoplasm		
				cellular protein metabolic process	-	nucleus		
				response to hormone stimulus	-	-		
				response to transforming growth factor beta stimulus	-	-		
				cellular response to tumor necrosis factor	-	-		
				negative regulation of cell proliferation	-	-		
				response to vitamin A	-	-		
				response to lipopolysaccharide	-	-		
47523	PREDICTED: up-regulator of cell proliferation-like, partial [*Pundamilia nyererei*]	XP_005755152	3.76E-82	cellular process	hydrolase activity, acting on acid anhydrides	membrane	2.23	2.81
				-	nucleotide binding	-		
44483	Hemoglobin beta 5 [*Gadus morhua*]	ACV69854	4.00E-53	oxygen transport	heme binding	hemoglobin complex	2.23	2.02
				-	oxygen transporter activity	-		
				-	iron ion binding	-		

				-	oxygen binding	-		
47108	PREDICTED: apolipoprotein B-100-like [*Cynoglossus semilaevis*]	XP_008311263	5.85E-11	N/A			2.20	1.77
52451	No significant blastx hit			N/A			2.05	1.55
46230	Nattectin precursor [*Anoplopoma fimbria*]	ACQ58341	7.10E-31	N/A			2.04	2.13
36678	Selenoprotein Ja [*Salmo salar*]	NP_001180426	0.00E+00	N/A	-	-	2.03	1.82
37064	Glutathione peroxidase 3 (plasma) precursor [*Danio rerio*]	NP_001131027	4.60E-79	hydrogen peroxide catabolic process	glutathione peroxidase activity	extracellular space	1.92	2.44
				lipid metabolic process	transcription factor binding	-		
				oxidation-reduction process	glutathione binding	-		
				response to corticosterone stimulus	selenium binding	-		
				female pregnancy	-	-		
				glutathione metabolic process	-	-		
				response to lipid hydroperoxide	-	-		
				response to molecule of fungal origin	-	-		
				response to drug	-	-		
				protein homotetramerization	-	-		
				response to selenium ion	-	-		
47023	No significant blastx hit			N/A			1.90	1.56
42143	SHC SH2 domain-binding protein 1 [*Danio rerio*]	NP_956137	1.22E-48	-	SH2 domain binding	-	1.89	1.69
54034	No significant blastx hit			N/A			1.88	1.61
46732	No significant blastx hit			N/A			1.88	2.01
46870	DNA excision repair protein ERCC-6-like [*Danio rerio*]	NP_001093563	1.43E-09	cellular process	heterocyclic compound binding	chromosome, centromeric region	1.86	1.76
				-	organic cyclic compound binding	-		
49405	No significant blastx hit			N/A			1.80	1.76
42084	Aurora kinase B [*Danio rerio*]	NP_997731	2.47E-33	negative regulation of B cell apoptotic process	protein binding	chromatin	1.79	1.59
				protein localization to kinetochore	protein serine/threonine/tyrosine kinase activity	chromocenter		
				negative regulation of transcription from RNA polymerase II promoter	metal ion binding	cytosol		
				multicellular organismal development	histone kinase activity (H3-S10 specific)	spindle		
				mitotic prometaphase	ATP binding	midbody		
				regulation of chromosome segregation	-	chromosome passenger complex		
				cleavage furrow formation	-	condensed nuclear chromosome, centromeric region		
				attachment of spindle microtubules to kinetochore	-	-		
				positive regulation of cytokinesis	-	-		
				protein autophosphorylation	-	-		
				cellular response to UV	-	-		
				anaphase-promoting complex-dependent proteasomal ubiquitin-dependent protein catabolic process	-	-		
				spindle midzone assembly involved in mitosis	-	-		
				histone H3-S10 phosphorylation	-	-		
46497	MIS18 binding protein 1 a [*Xenopus laevis*]	NP_0012432	3.94E-07	N/A			1.77	1.50

53197	No significant blastx hit			N/A			1.77	1.53
38929	Maternal embryonic luecine zipper kinase v4 [*Homo sapiens*]	BAF74635	2.57E-10	protein autophosphorylation	protein serine/threonine kinase activity	cell cortex	1.76	1.58
				regulation of heart contraction	calcium ion binding	plasma membrane		
				neural precursor cell proliferation	lipid binding	-		
				G2/M transition of mitotic cell cycle	ATP binding	-		
				erythrocyte development	protein binding	-		
				positive regulation of apoptotic process	non-membrane spanning protein tyrosine kinase activity	-		
50072	No significant blastx hit			N/A			1.74	1.49
39819	Nuclear receptor subfamily 4 group A member 1 [*Danio rerio*]	NP_001002173	5.58E-162	positive regulation of endothelial cell proliferation	RNA polymerase II core promoter proximal region sequence-specific DNA binding transcription factor activity involved in positive regulation of transcription	transcription factor complex	1.74	1.47
				transcription initiation from RNA polymerase II promoter	zinc ion binding	-		
				neurotrophin TRK receptor signaling pathway	steroid hormone receptor activity	-		
				cellular response to vascular endothelial growth factor stimulus	ligand-activated sequence-specific DNA binding RNA polymerase II transcription factor activity	-		
				epidermal growth factor receptor signaling pathway	protein heterodimerization activity	-		
				negative regulation of cysteine-type endopeptidase activity involved in apoptotic process	sequence-specific DNA binding	-		
				steroid hormone mediated signaling pathway	protein homodimerization activity	-		
				phosphatidylinositol-mediated signaling	-	-		
				apoptotic process	-	-		
				response to inorganic substance	-	-		
				intracellular receptor signaling pathway	-	-		
				positive regulation of transcription from RNA polymerase II promoter	-	-		
				endothelial cell chemotaxis	-	-		
				fibroblast growth factor receptor signaling pathway	-	-		
				cell migration involved in sprouting angiogenesis	-	-		
52979	No significant blastx hit			N/A			1.72	1.72
39037	Iodotyrosine dehalogenase 1 precursor [*Anoplopoma fimbria*]	ACQ57841	4.15E-24	thyroid hormone generation	iodide peroxidase activity	integral to membrane	1.71	1.54
				oxidation-reduction process	-	plasma membrane		
				cellular nitrogen compound metabolic process	-	-		
47768	PREDICTED: cell division cycle-associated protein 3 isoform X2 [*Poecilia reticulata*]	XP_008429618	4.17E-35	N/A			1.66	1.53
37946	Voltage-dependent anion-selective channel protein 2 [*Salmo salar*]	NP_001239278	3.83E-175	negative regulation of intrinsic apoptotic signaling pathway	voltage-gated anion channel activity	pore complex	1.66	1.63
				fin regeneration	protein binding	mitochondrial inner membrane		
				negative regulation of protein	nucleotide binding	mitochondrial nucleoid		

				polymerization				
				transmembrane transport	porin activity	mitochondrial outer membrane		
				regulation of anion transport	-	-		
54680	No significant blastx hit			N/A			1.66	1.52
45663	No significant blastx hit			N/A			1.66	1.64
40996	Selenoprotein U [*Oryzias latipes*]	NP_001180474	1.62E-37	regulation of osteoclast differentiation	antioxidant activity	mitochondrion	1.65	1.78
				oxidation-reduction process	-	-		
51459	No significant blastx hit			N/A			1.64	1.36
38840	S-phase kinase-associated protein 2 (p45) [*Danio rerio*]	NP_001076306	6.82E-82	S phase of mitotic cell cycle	identical protein binding	cytosol	1.64	1.52
				regulation of cell cycle	ubiquitin-protein ligase activity	SCF ubiquitin ligase complex		
				protein polyubiquitination	-	nucleoplasm		
				G2/M transition of mitotic cell cycle	-	-		
				positive regulation of intracellular estrogen receptor signaling pathway	-	-		
				anaphase-promoting complex-dependent proteasomal ubiquitin-dependent protein catabolic process	-	-		
				G1 phase of mitotic cell cycle	-	-		
				G1/S transition of mitotic cell cycle	-	-		
				cellular response to cell-matrix adhesion	-	-		
				positive regulation of smooth muscle cell proliferation	-	-		
46257	No significant blastx hit			N/A			1.63	1.41
36708	Cyclin B1 [*Oncorhynchus mykiss*]	NP_001118130	1.18E-86	mitotic prometaphase	patched binding	cytosol	1.61	1.57
				G1/S transition of mitotic cell cycle	protein kinase binding	nucleoplasm		
				cell division	-	spindle pole		
				mitotic metaphase plate congression	-	centrosome		
				anaphase-promoting complex-dependent proteasomal ubiquitin-dependent protein catabolic process	-	condensed nuclear chromosome outer kinetochore		
				positive regulation of attachment of spindle microtubules to kinetochore	-	-		
				mitotic spindle checkpoint	-	-		
				mitotic spindle stabilization	-	-		
				positive regulation of ubiquitin-protein ligase activity involved in mitotic cell cycle	-	-		
				positive regulation of mitotic cell cycle	-	-		
				multicellular organismal development	-	-		
				G2/M transition of mitotic cell cycle	-	-		
				regulation of cyclin-dependent protein serine/threonine kinase activity	-	-		
				cellular response to chemical stimulus	-	-		
				anatomical structure development	-	-		
36709	G2/mitotic-specific cyclin-B1 [*Salmo salar*]	ACI68598	3.94E-91	regulation of gene expression	patched binding	cytosol	1.60	1.66
				cell development	protein kinase binding	nucleoplasm		
				mitotic prometaphase	kinase activity	spindle pole		
				G1/S transition of mitotic cell cycle	-	centrosome		
				response to abiotic stimulus	-	condensed nuclear chromosome outer kinetochore		
				cell division	-	-		

				mitotic metaphase plate congression	-	-		
				regulation of nucleobase-containing compound metabolic process	-	-		
				cardiac muscle tissue development	-	-		
				negative regulation of macromolecule metabolic process	-	-		
				nucleic acid metabolic process	-	-		
				regulation of chromosome organization	-	-		
				anaphase-promoting complex-dependent proteasomal ubiquitin-dependent protein catabolic process	-	-		
				positive regulation of attachment of spindle microtubules to kinetochore	-	-		
				mitotic spindle checkpoint	-	-		
				gamete generation	-	-		
				mitotic spindle stabilization	-	-		
				positive regulation of ubiquitin-protein ligase activity involved in mitotic cell cycle	-	-		
				positive regulation of mitotic cell cycle	-	-		
				cellular response to organic substance	-	-		
				muscle structure development	-	-		
				developmental growth	-	-		
				G2/M transition of mitotic cell cycle	-	-		
				response to stress	-	-		
				regulation of cyclin-dependent protein serine/threonine kinase activity	-	-		
37919	Inner centromere protein [*Oryzias latipes*]	NP_001153903	3.53E-58	cytokinesis	protein kinase binding	chromosome passenger complex	1.59	1.38
				spindle assembly	-	cytosol		
				chromosome segregation	-	centromeric heterochromatin		
				mitotic prometaphase	-	synaptonemal complex		
				-	-	kinetochore		
				-	-	spindle		
52245	No significant blastx hit			N/A			1.59	1.34
36423	Cell division cycle protein 20 homolog [*Salmo salar*]	NP_001133251	0.00E+00	positive regulation of ubiquitin-protein ligase activity involved in mitotic cell cycle	protein C-terminus binding	nucleoplasm	1.59	1.68
				positive regulation of synapse maturation	enzyme binding	anaphase-promoting complex		
				mitotic prometaphase	-	cytosol		
				positive regulation of synaptic plasticity	-	centrosome		
				mitotic spindle assembly checkpoint	-	perinuclear region of cytoplasm		
				negative regulation of ubiquitin-protein ligase activity involved in mitotic cell cycle		spindle pole		
				cell division	-	-		
				positive regulation of cell proliferation	-	-		
				activation of anaphase-promoting complex activity	-	-		
				regulation of meiosis	-	-		
				anaphase-promoting complex-dependent proteasomal ubiquitin-dependent protein catabolic process		-		
				regulation of dendrite development	-	-		
40140	Dolichyl-diphosphooligosaccharide--protein glycosyltransferase subunit STT3A [*Salmo salar*]	NP_001135197	2.42E-132	co-translational protein modification	protein binding	oligosaccharyltransferase complex	1.58	1.39

				protein N-linked glycosylation via asparagine	dolichyl-diphosphooligosaccharide-protein glycotransferase activity	integral to membrane		
				post-translational protein modification	-	-		
43176	AF506219_1valyl-tRNA synthetase [*Danio rerio*]	AAM34663	1.55E-91	response to virus	valine-tRNA ligase activity	cytoplasmic part	1.57	1.61
				valyl-tRNAaminoacylation	aminoacyl-tRNA editing activity	-		
				translational elongation	ATP binding	-		
				-	protein binding	-		
40684	5-formyltetrahydrofolate cyclo-ligase [*Ictalurus furcatus*]	ADO28036	1.75E-91	tetrahydrofolate metabolic process	5-formyltetrahydrofolate cyclo-ligase activity	cytosol	1.56	1.34
				folic acid-containing compound biosynthetic process	folic acid binding	mitochondrion		
				cell surface receptor signaling pathway	transmembrane signaling receptor activity	Golgi apparatus		
				formate metabolic process	ATP binding	plasma membrane		
48811	No significant blastx hit			N/A			1.56	1.47
37918	Inner centromere protein [*Oryzias latipes*]	NP_001153903	7.56E-15	cytokinesis	protein kinase binding	chromosome passenger complex	1.55	1.43
				spindle assembly	-	cytosol		
				chromosome segregation	-	centromeric heterochromatin		
				mitotic prometaphase	-	synaptonemal complex		
				-	-	kinetochore		
				-	-	spindle		
36707	Cyclin A2 [*Danio rerio*]	AAH68323	2.92E-09	Ras protein signal transduction	calcium channel activity	nucleoplasm	1.55	1.52
				mitotic G2 DNA damage checkpoint	protein kinase binding	integral to membrane		
				organ regeneration	-	pronucleus		
				response to glucagon stimulus	-	-		
				cell division	-	-		
				regulation of cyclin-dependent protein serine/threonine kinase activity	-	-		
				retina development in camera-type eye	-	-		
				calcium ion transmembrane transport	-	-		
				positive regulation of fibroblast proliferation	-	-		
				response to estradiol stimulus	-	-		
45225	PREDICTED: uncharacterized protein LOC102782392 isoform X2 [*Neolamprologus brichardi*]	XP_006805322	3.18E-96	N/A			1.55	1.43
54914	No significant blastx hit			N/A			1.55	1.39
39900	FK506 binding protein 1-like [*Danio rerio*]	NP_001005594	1.65E-68	regulation of activin receptor signaling pathway	type I transforming growth factor beta receptor binding	terminal cisterna	1.54	1.49
				transforming growth factor beta receptor signaling pathway	transforming growth factor beta-activated receptor activity	cytosol		
				protein refolding	FK506 binding	calcium channel complex		
				positive regulation of I-kappaB kinase/NF-kappaB cascade	ryanodine-sensitive calcium-release channel activity	axon		
				positive regulation of protein binding	ion channel binding	sarcoplasmic reticulum membrane		
				regulation of immune response	cyclic nucleotide binding	-		
				response to hydrogen peroxide	peptidyl-prolylcis-trans isomerase activity	-		

				brain development	SMAD binding	-		
				'de novo' protein folding	activin binding	-		
				heart trabecula formation	-	-		
				protein peptidyl-prolyl isomerization	-	-		
				regulation of voltage-gated calcium channel activity	-	-		
				positive regulation of protein ubiquitination	-	-		
				negative regulation of protein phosphorylation	-	-		
				cytokine-mediated signaling pathway	-	-		
				embryonic heart tube development	-	-		
				regulation of ryanodine-sensitive calcium-release channel activity	-	-		
				negative regulation of heart rate	-	-		
				regulation of cardiac muscle contraction by regulation of the release of sequestered calcium ion	-	-		
				embryo development ending in birth or egg hatching	-	-		
				response to caffeine	-	-		
				ventricular cardiac muscle tissue morphogenesis	-	-		
				protein maturation by protein folding	-	-		
				negative regulation of insulin secretion involved in cellular response to glucose stimulus	-	-		
				smooth muscle contraction	-	-		
				limb development	-	-		
				extracellular fibril organization	-	-		
				response to vitamin E	-	-		
				response to redox state	-	-		
				neuronal action potential propagation	-	-		
				T cell proliferation	-	-		
				beta-amyloid formation	-	-		
				negative regulation of protein phosphatase type 2B activity	-	-		
				response to retinoic acid	-	-		
				SMAD protein complex assembly	-	-		
				lung development	-	-		
39611	TBC1 domain family member 5 [*Danio rerio*]	NP_956905	1.22E-62	positive regulation of RabGTPase activity	RabGTPase activator activity	retromer complex	1.53	1.41
				-	protein binding	intracellular		
42092	Chain B, Crystal Structure Of Polo-like Kinase 1 [*Danio rerio*]	4J7B_B	3.47E-31	regulation of mitotic anaphase	protein serine/threonine kinase activity	midbody	1.52	1.58
				mitotic prometaphase	ATP binding	cytosol		
				G2 DNA damage checkpoint	protein kinase binding	nucleoplasm		
				polar body extrusion after meiotic divisions	anaphase-promoting complex binding	spindle midzone		
				protein localization to chromatin	microtubule binding	spindle microtubule		
				activation of mitotic anaphase-promoting complex activity	-	spindle pole		
				mitotic prophase	-	centrosome		
				negative regulation of cyclin-dependent protein serine/threonine kinase activity	-	condensed nuclear chromosome outer kinetochore		
				response to antibiotic	-	-		

				protein destabilization	-	-		
				regulation of protein binding	-	-		
				microtubule bundle formation	-	-		
				negative regulation of transcription from RNA polymerase II promoter	-	-		
				peptidyl-serine phosphorylation	-	-		
				cell proliferation	-	-		
				centrosome organization	-	-		
				G2/M transition of mitotic cell cycle	-	-		
				negative regulation of apoptotic process	-	-		
				positive regulation of peptidyl-threonine phosphorylation	-	-		
				oocyte maturation	-	-		
50859	No significant blastx hit			N/A			1.52	1.69
40625	Small nuclear ribonucleoprotein F, partial [*Bos mutus*]	ELR54499	1.53E-43	termination of RNA polymerase II transcription	RNA binding	cytosol	1.52	1.40
				spliceosomalsnRNP assembly	-	U7 snRNP		
				histone mRNA metabolic process	-	catalytic step 2 spliceosome		
				ncRNA metabolic process	-	nucleoplasm		
				-	-	U12-type spliceosomal complex		
40045	Chain B, Crystal Structure Of Polo-like Kinase 1 [*Danio rerio*]	4J7B_B	1.50E-45	regulation of mitotic anaphase	protein serine/threonine kinase activity	midbody	1.51	1.60
				mitotic prometaphase	ATP binding	cytosol		
				G2 DNA damage checkpoint	protein kinase binding	nucleoplasm		
				polar body extrusion after meiotic divisions	anaphase-promoting complex binding	spindle midzone		
				protein localization to chromatin	microtubule binding	spindle microtubule		
				activation of mitotic anaphase-promoting complex activity	-	spindle pole		
				mitotic prophase	-	centrosome		
				negative regulation of cyclin-dependent protein serine/threonine kinase activity	-	condensed nuclear chromosome outer kinetochore		
				response to antibiotic	-	-		
				protein destabilization	-	-		
				regulation of protein binding	-	-		
				microtubule bundle formation	-	-		
				negative regulation of transcription from RNA polymerase II promoter	-	-		
				peptidyl-serine phosphorylation	-	-		
				cell proliferation	-	-		
				centrosome organization	-	-		
				G2/M transition of mitotic cell cycle	-	-		
				negative regulation of apoptotic process	-	-		
				positive regulation of peptidyl-threonine phosphorylation	-	-		
				oocyte maturation	-	-		
43805	Protein Spindly [*Danio rerio*]	NP_001098412	7.53E-81	mitotic metaphase plate congression	kinetochore binding	spindle pole	1.50	1.34
				cell division	-	condensed chromosome outer kinetochore		
				protein localization to kinetochore	-	cytoplasmic part		
				establishment of mitotic spindle orientation	-	nucleus		
53578	No significant blastx hit			N/A			1.50	1.43
41630	Putative iodothyronine deiodinase type 1	CAF02303	4.41E-44	hormone metabolic process	oxidoreductase activity	endoplasmic reticulum	1.49	1.86

	[*Sparus aurata*]							
				cellular metabolic process	-	membrane part		
				single-organism metabolic process	-	-		
48506	No significant blastx hit			N/A			1.49	1.52
38727	Kinetochore protein Spc25 [*Osmerus mordax*]	ACO09526	2.04E-56	mitotic prometaphase	protein binding	Ndc80 complex	1.49	1.40
				translation	-	condensed nuclear chromosome outer kinetochore		
				mitotic spindle organization	-	endoplasmic reticulum membrane		
				energy reserve metabolic process	-	cytosol		
				establishment of mitotic spindle orientation	-	-		
				attachment of spindle microtubules to kinetochore	-	-		
				regulation of insulin secretion	-	-		
				mitotic sister chromatid segregation	-	-		
				small molecule metabolic process	-	-		
				SRP-dependent cotranslational protein targeting to membrane	-	-		
50048	No significant blastx hit			N/A			1.48	1.26
36946	Dual specificity protein kinase Ttk [*Danio rerio*]	NP_778207	1.51E-149	positive regulation of pathway-restricted SMAD protein phosphorylation	protein serine/threonine/tyrosine kinase activity	spindle	1.48	1.37
				mitotic spindle organization	protein tyrosine kinase activity	-		
				mitotic spindle assembly checkpoint	metal ion binding	-		
				cell division	protein serine/threonine kinase activity	-		
				anatomical structure homeostasis	ATP binding	-		
				fin regeneration	-	-		
				female meiosis chromosome segregation	-	-		
				positive regulation of cell proliferation	-	-		
				peptidyl-serine phosphorylation	-	-		
				male meiosis chromosome segregation	-	-		
				embryo development	-	-		
40111	PREDICTED: protein regulator of cytokinesis 1-like isoform X1 [*Stegastes partitus*]	XP_0082879 42	1.25E-11	N/A			1.48	1.44
41486	Serine/threonine-protein kinase 6 [*Dicentrarchus labrax*]	CBN80821	9.97E-106	regulation of protein stability	protein kinase binding	spindle pole centrosome	1.47	1.45
				cell division	protein serine/threonine/tyrosine kinase activity	cytosol		
				protein autophosphorylation	kinesin binding	midbody		
				cell projection organization	microtubule binding	perinuclear region of cytoplasm		
				peptidyl-serine phosphorylation	protein serine/threonine kinase activity	spindle microtubule		
				phosphatidylinositol-mediated signaling	ubiquitin protein ligase binding	nucleus		
				spindle assembly involved in mitosis	ATP binding	-		
				positive regulation of mitosis	-	-		
				anaphase-promoting complex-dependent proteasomal ubiquitin-dependent protein catabolic process	-	-		
				regulation of centrosome cycle	-	-		
				response to estradiol stimulus	-	-		

42045	Fep15 selenoprotein precursor [*Oryzias latipes*]	NP_001182373	5.58E-58	-	-	cytoplasmic part	1.47	1.53
				-	-	intracellular membrane-bounded organelle		
41451	Protein disulfide-isomerase a4 [*Perca flavescens*]	ADX97231	2.24E-104	cell redox homeostasis	electron carrier activity	cell surface	1.47	1.34
				glycerol ether metabolic process	protein binding	endoplasmic reticulum lumen		
				protein secretion	protein disulfide isomerase activity	melanosome		
				oxidation-reduction process	protein disulfide oxidoreductase activity	-		
44994	Kinesin-like protein KIF2C [*Danio rerio*]	NP_001108065	6.64E-16	microtubule depolymerization	microtubule motor activity	cytoplasmic microtubule	1.47	1.53
				cell division	microtubule plus-end binding	condensed chromosome kinetochore		
				microtubule-based movement	ATP binding	nucleus		
				mitosis	-	-		
				regulation of chromosome segregation	-	-		
				establishment or maintenance of microtubule cytoskeleton polarity	-	-		
44207	No significant blastx hit			N/A			1.47	1.36
42544	Thioredoxin reductase 3a [*Oncorhynchus mykiss*]	CCX35035	5.38E-76	transport	protein disulfide oxidoreductase activity	nucleolus	1.47	1.35
				hydrogen peroxide catabolic process	NAD(P)H oxidase activity	cytosol		
				NADPH oxidation	flavin adenine dinucleotide binding	neuronal cell body		
				response to selenium ion	selenatereductase activity	endoplasmic reticulum		
				cellular response to hyperoxia	mercury ion binding	mitochondrion		
				cellular lipid metabolic process	NADP binding	-		
				spermatogenesis	electron carrier activity	-		
				selenocysteine metabolic process	thioredoxin-disulfide reductase activity	-		
				cell proliferation	protein homodimerization activity	-		
				benzene-containing compound metabolic process	-	-		
				placenta development	-	-		
				electron transport chain	-	-		
				cell redox homeostasis	-	-		
				methylmercury metabolic process	-	-		
				response to axon injury	-	-		
				signal transduction	-	-		
				nucleobase-containing small molecule interconversion	-	-		
				response to drug	-	-		
				positive regulation of cell death	-	-		
				cell differentiation	-	-		
				glutathione metabolic process	-	-		
				halogen metabolic process	-	-		
				mesoderm formation	-	-		
				protein tetramerization	-	-		
				cellular response to copper ion	-	-		
39333	Nicotinamide riboside kinase 2 [*Esox lucius*]	ACO14184	1.28E-97	NAD biosynthetic process	metal ion binding	intracellular	1.47	1.41
				integrin-mediated signaling pathway	ATP binding	-		
				negative regulation of myoblast	protein binding	-		

				differentiation				
				cell-substrate adherens junction assembly	ribosylnicotinamide kinase activity	-		
				phosphorylation	-	-		
				basement membrane assembly	-	-		
42051	Selenoprotein T2 precursor [*Danio rerio*]	NP_001091957	4.74E-70	selenocysteine incorporation	selenium binding	-	1.45	1.54
				cell redox homeostasis	-	-		
42043	Selenoprotein H [*Danio rerio*]	NP_835234	1.93E-27	N/A			1.45	1.68
50960	No significant blastx hit			N/A			1.45	1.31
53555	No significant blastx hit			N/A			1.44	1.41
42268	U2 small nuclear ribonucleoprotein B [*Osmerus mordax*]	ACO09361	6.43E-120	mRNA splicing, via spliceosome	snRNA binding	U2 snRNP	1.44	1.46
				-	protein binding	nucleoplasm		
				-	nucleotide binding	catalytic step 2 spliceosome		
49031	No significant blastx hit			N/A			1.44	1.46
42245	Small nuclear ribonucleoprotein G [*Salmo salar*]	ACM09010	2.37E-41	oxidation-reduction process	RNA binding	cytosol	1.42	1.38
				termination of RNA polymerase II transcription	protein binding	U7 snRNP		
				spliceosomal complex assembly	dihydrolipoyl dehydrogenase activity	catalytic step 2 spliceosome		
				spliceosomalsnRNP assembly	-	nucleoplasm		
				histone mRNA metabolic process	-	U12-type spliceosomal complex		
				ncRNA metabolic process	-	-		
50073	No significant blastx hit			N/A			1.42	1.41
36791	Cytosolic nonspecific dipeptidase [*Oreochromis niloticus*]	NP_001266471	4.07E-90	xenobiotic metabolic process	metal ion binding	cytosol	1.42	1.39
				proteolysis	carboxypeptidase activity	-		
				glutathione biosynthetic process	dipeptidase activity	-		
				sulfur amino acid metabolic process	metallopeptidase activity	-		
				-	tripeptidase activity	-		
42623	TRAF-interacting protein [*Dicentrarchus labrax*]	CBN80995	2.60E-31	-	metal ion binding	-	1.42	1.60
41459	FAM136A [*Salmo salar*]	ACI69219	1.20E-33	-	-	mitochondrion	1.41	1.51
36563	Coiled-coil domain-containing protein 43 [*Osmerus mordax*]	ACO10099	3.47E-75	N/A			1.41	1.42
48765	PREDICTED: uncharacterized protein LOC103355395 [*Stegastes partitus*]	XP_008277405	1.47E-83	N/A			1.40	1.37
42091	Serine/threonine-protein kinase PLK1 [*Xenopus (Silurana) tropicalis*]	NP_998844	1.43E-15	regulation of mitotic anaphase	protein serine/threonine kinase activity	midbody	1.39	1.39
				mitotic prometaphase	ATP binding	cytosol		
				G2 DNA damage checkpoint	protein kinase binding	nucleoplasm		
				polar body extrusion after meiotic divisions	anaphase-promoting complex binding	spindle midzone		
				protein localization to chromatin	microtubule binding	spindle microtubule		
				activation of mitotic anaphase-promoting complex activity	-	spindle pole		
				mitotic prophase	-	centrosome		
				negative regulation of cyclin-dependent protein serine/threonine kinase activity	-	condensed nuclear chromosome outer kinetochore		
				response to antibiotic	-	-		
				protein destabilization	-	-		
				regulation of protein binding	-	-		

				microtubule bundle formation	-	-		
				negative regulation of transcription from RNA polymerase II promoter	-	-		
				peptidyl-serine phosphorylation	-	-		
				cell proliferation	-	-		
				centrosome organization	-	-		
				G2/M transition of mitotic cell cycle	-	-		
				negative regulation of apoptotic process	-	-		
				positive regulation of peptidyl-threonine phosphorylation	-	-		
				oocyte maturation	-	-		
36824	DEAD/H (Asp-Glu-Ala-Asp/His) box polypeptide 19 (DBP5 homolog, yeast) [*Danio rerio*]	AAH44541	1.83E-115	mRNA export from nucleus	RNA binding	nuclear membrane	1.39	1.33
				protein transport	ATP-dependent helicase activity	nuclear pore		
				-	ATP binding	cytoplasm		
51089	No significant blastx hit			N/A			1.39	1.32
39171	No significant blastx hit			N/A	-	-	1.38	1.39
39909	Periostin isoform 1 precursor [*Danio rerio*]	NP_001071254	2.74E-130	regulation of Notch signaling pathway	heparin binding	proteinaceous extracellular matrix	1.38	1.27
				muscle attachment	-	-		
				tissue development	-	-		
				cell adhesion	-	-		
				extracellular matrix organization	-	-		
47624	PREDICTED: uncharacterized protein LOC100709344 [*Oreochromis niloticus*]	XP_003447720	5.14E-37	N/A			1.37	1.32
54053	No significant blastx hit			N/A			1.37	1.48
40270	Actin-like 6A, isoform CRA_b [*Mus musculus*]	EDL35007	4.02E-46	histone H4 acetylation	protein binding	SWI/SNF complex	1.37	1.28
				regulation of transcription from RNA polymerase II promoter	transcription coactivator activity	npBAF complex		
				DNA recombination	chromatin binding	Ino80 complex		
				regulation of growth	ATP binding	NuA4 histone acetyltransferase complex		
				signal transduction	-	plasma membrane		
				chromatin remodeling	-	-		
				nervous system development	-	-		
				histone H2A acetylation	-	-		
				neural retina development	-	-		
				DNA repair	-	-		
54702	No significant blastx hit			N/A			1.37	1.46
35542	Lrpprc protein [*Danio rerio*]	AAI39520	1.03E-14	mitochondrion transport along microtubule	microtubule binding	nuclear membrane	1.37	1.35
				transcription, DNA-dependent	RNA binding	microtubule		
				-	actin filament binding	condensed nuclear chromosome		
				-	single-stranded DNA binding	mitochondrial nucleoid		
				-	beta-tubulin binding	perinuclear region of cytoplasm		
36838	Density-regulated protein [*Esox lucius*]	ACO14199	2.29E-93	translational initiation	protein binding	-	1.37	1.49
				-	translation initiation factor activity	-		
43817	No significant blastx hit			N/A			1.36	1.24
52227	No significant blastx hit			N/A			1.36	1.33
46293	No significant blastx hit			N/A			1.36	1.28

39030	Transmembrane protein 209 [*Danio rerio*]	NP_001257902	9.18E-20	-	-	integral to membrane	1.36	1.27
44206	tRNA (uracil(54)-C(5))-methyltransferase homolog-B [*Danio rerio*]	A4QP75.2	4.52E-53	methylation	RNA methyltransferase activity	mitochondrion	1.36	1.51
				RNA processing	-	-		
53712	No significant blastx hit			N/A			1.36	1.24
38123	Probable proline dehydrogenase 2 [*Dicentrarchus labrax*]	CBN81374	2.17E-32	locomotory behavior	spectrin binding	membrane raft	1.36	1.34
				glomerular visceral epithelial cell development	proline dehydrogenase activity	mitochondrial matrix		
				cell adhesion	alpha-actinin binding	slit diaphragm		
				glomerular basement membrane development	protein domain specific binding	mitochondrial inner membrane		
				regulation of excretion	-	protein complex		
				glutamate biosynthetic process	-	cell projection		
				myoblast fusion	-	integral to plasma membrane		
				JNK cascade	-	-		
				oxidation-reduction process	-	-		
				phototaxis	-	-		
				positive regulation of actin filament polymerization	-	-		
				proline catabolic process to glutamate	-	-		
40745	NUP160 protein, partial [*Homo sapiens*]	AAH08700	3.61E-23	transmembrane transport	nucleocytoplasmic transporter activity	cytosol	1.34	1.31
				carbohydrate metabolic process	protein binding	nuclear pore outer ring		
				cytokine-mediated signaling pathway	-	kinetochore		
				mitotic prometaphase	-	-		
				mRNA export from nucleus	-	-		
				viral process	-	-		
				small molecule metabolic process	-	-		
				regulation of glucose transport	-	-		
				protein transport	-	-		
43404	Centrin-3 [*Anoplopoma fimbria*]	ACQ58795	1.48E-76	mitosis	G-protein beta/gamma-subunit complex binding	photoreceptor connecting cilium	1.34	1.40
				centrosome cycle	microtubule binding	intermediate filament cytoskeleton		
				cell division	calcium ion binding	microtubule basal body		
				-	-	centriole		
48902	No significant blastx hit			N/A			1.33	1.59
46976	PREDICTED: DNA-(apurinic or apyrimidinic site) lyase-like [*Danio rerio*]	XP_686479	1.19E-10	N/A			1.33	1.55
44890	No significant blastx hit			N/A			1.33	1.28
41087	Nucleoporin NDC1-like protein [*Cricetulus griseus*]	ERE83000	7.74E-16	nuclear pore complex assembly	structural constituent of nuclear pore	nuclear membrane	1.33	1.29
				nuclear pore distribution	-	nuclear pore		
				protein transport	-	-		
				mRNA transport	-	-		
48760	No significant blastx hit			N/A			1.33	1.28
36816	ATP-dependent RNA helicase DDX39 [*Salmo salar*]	NP_001134851	3.60E-105	RNA secondary structure unwinding	ATP-dependent protein binding	U4 snRNP	1.33	1.25
				spliceosomal complex assembly	ATP-dependent RNA helicase activity	spliceosomal complex		
				intronless viral mRNA export from host nucleus	ATP binding	nuclear speck		
				-	U4 snRNA binding	U6 snRNP		
				-	U6 snRNA binding	transcription export complex		

				-	-	cytoplasm		
54882	No significant blastx hit			N/A			1.33	1.40
54133	No significant blastx hit			N/A			1.33	1.37
52194	No significant blastx hit			N/A			1.32	1.38
38722	No significant blastx hit			N/A			1.32	1.26
51659	No significant blastx hit			N/A			1.31	1.29
52246	No significant blastx hit			N/A			1.31	1.30
39739	60S ribosome subunit biogenesis protein NIP7 homolog [*Tetraodon nigroviridis*]	Q4T2X8	1.82E-117	ribosome assembly	RNA binding	nucleolus	1.31	1.37
				-	protein binding	-		
53169	No significant blastx hit			N/A			1.30	1.36
50162	No significant blastx hit			N/A			1.30	1.33
43247	WD repeat protein 57 [*Osmerus mordax*]	ACO09549	1.45E-100	mRNA splicing, via spliceosome	nucleic acid binding	cytoplasm	1.30	1.34
				-	protein binding	nucleolus		
				-	-	U5 snRNP		
				-	-	nucleoplasm		
				-	-	small nucleolarribonucleoprotein complex		
				-	-	catalytic step 2 spliceosome		
38645	Fatty acid-binding protein 2b [*Lates calcarifer*]	AGL33438	1.25E-77	transport	lipid binding	-	1.30	1.26
				-	transporter activity	-		
47776	No significant blastx hit			N/A			1.30	1.29
43465	Chromatin accessibility complex protein 1 [*Osmerus mordax*]	ACO09866	1.93E-33	chromatin remodeling	DNA-directed DNA polymerase activity	epsilon DNA polymerase complex	1.30	1.29
				-	sequence-specific DNA binding	CHRAC		
				-	protein heterodimerization activity	-		
36704	Cyclic AMP phosphoprotein [*Scophthalmus maximus*]	ABJ98640	5.85E-51	positive regulation of Ras protein signal transduction	protein phosphatase type 2A regulator activity	cytoplasm	1.29	1.26
				G2/M transition of mitotic cell cycle	protein phosphatase 2A binding	-		
				positive regulation of glucose import	protein phosphatase inhibitor activity	-		
				cell division	potassium channel regulator activity	-		
				positive regulation of gluconeogenesis	receptor binding	-		
				negative regulation of catalytic activity	protein kinase binding	-		
				mitosis	-	-		
				dopamine receptor signaling pathway	-	-		
41063	Tetratricopeptide repeat protein 27 [*Danio rerio*]	NP_775392.2	4.31E-51	N/A			1.29	1.33
52121	No significant blastx hit			N/A			1.29	1.29
43241	WD repeat-containing protein 1-A [*Salmo salar*]	ACN58683	4.50E-68	platelet activation	actin binding	cytosol	1.29	1.26
				actin filament fragmentation	catalytic activity	actin filament		
				metabolic process	-	extracellular region		
				sensory perception of sound	-	nucleus		
				platelet degranulation	-	plasma membrane		
43286	Serine/threonine-protein kinase 6 [*Dicentrarchus labrax*]	CBN80821	1.20E-14	N/A			1.29	1.37
39109	Mitochondrial import inner membrane translocase subunit Tim13 [*Osmerus mordax*]	ACO09710	2.87E-14	chaperone-mediated protein transport	zinc ion binding	mitochondrial inner membrane	1.28	1.32
				cellular protein metabolic process	-	mitochondrial		

						intermembrane space protein transporter complex		
				protein import into mitochondrial inner membrane	-	-		
				sensory perception of sound	-	-		
44499	Hemoglobin alpha 1 chain [*Gadus morhua*]	ACJ66341	1.63E-98	oxygen transport	oxygen transporter activity	hemoglobin complex	1.28	1.29
				response to hypoxia	iron ion binding	-		
				-	heme binding	-		
				-	oxygen binding	-		
48392	No significant blastx hit			N/A			1.27	1.25
52149	No significant blastx hit			N/A			1.27	1.28
52974	No significant blastx hit			N/A			1.27	1.26
41294	Prefoldin subunit 5 [*Osmerus mordax*]	ACO09647	3.21E-10	protein folding	protein binding	cytoplasm	1.26	1.23
				negative regulation of canonical Wnt receptor signaling pathway	-	-		
				retina development in camera-type eye	-	-		
				negative regulation of transcription, DNA-dependent	-	-		
41458	FAM136A [*Salmo salar*]	ACI69219	5.71E-68	-	-	mitochondrion	1.26	1.35
43399	Peptidyl-tRNA hydrolase 2, mitochondrial precursor [*Salmo salar*]	ACI69416	3.28E-84	negative regulation of anoikis	aminoacyl-tRNA hydrolase activity	mitochondrion	1.26	1.36
				metabolic process	protein binding	-		
45415	FAM44B [*Salmo salar*]	ACI69898	1.39E-15	mitosis	-	microtubule organizing center	1.25	1.27
				cell division	-	condensed chromosome kinetochore		
43327	Dehydrogenase/reductase SDR family member 13 precursor [*Salmo salar*]	ACM08484	1.52E-23	oxidation-reduction process	oxidoreductase activity	-	1.23	1.34
				-	nucleotide binding	-		
50611	No significant blastx hit			N/A			1.23	1.30
53575	No significant blastx hit			N/A			1.23	1.18

[1]Probe identifier (ID) numbers are 5-digit unique identifiers for the 50mer probes on the Atlantic cod 20K microarray (Booman et al. 2011).
[2]The BLASTx hit with the lowest E-value and a protein name (e.g. not "unnamed protein product" or "predicted") is shown.
[3]GO terms in this table were collected using Blast2GO (see Methods).
