# Supplementary material for: Transcriptome profiling reveals that feeding wild zooplankton to larval Atlantic cod (Gadus morhua) influences suites of genes involved in oxidation-reduction, mitosis, and selenium homeostasis
Source: BMC Genomics. 2015 Nov 26;16:1016. doi: 10.1186/s12864-015-2120-1 (PMC4661974; doi:10.1186/s12864-015-2120-1)
Supplement: Additional file 2: Table S2. — Microarray-identified genes significantly down-regulated in RA-Zoo compared with both RA and RA-PH. (PDF 310kb) [file 12864_2015_2120_MOESM2_ESM.pdf]

Supplemental Table 2. Microarray-identified genes significantly down-regulated in RA-Zoo compared with both RA and RA-PH.

Probe identifier[1]	BLASTx identification			GO terms[3]			Microarray fold change in RA-Zoo versus:	
	Best named BLASTx hit[2]	Accession #	E-Value	BP	MF	CC	RA	RA-PH
48680	Zinc finger MYM-type protein 1 [*Zootermopsis nevadensis*]	KDR18839	3.49E-17	-	binding	-	-5.24	-2.60
45497	No significant blastx hit			N/A			-3.73	-3.92
55296	No significant blastx hit			N/A			-3.48	-3.37
37713	Flavin containing monooxygenase 5 [*Danio rerio*]	CAI21028	2.32E-49	xenobiotic metabolic process	flavin adenine dinucleotide binding	integral to membrane	-3.15	-4.26
				NADPH oxidation	NADP binding	intrinsic to endoplasmic reticulum membrane		
				toxin metabolic process	N,N-dimethylanilinemonooxygenase activity	-		
				oxygen metabolic process	-	-		
				organic acid metabolic process	-	-		
				drug metabolic process	-	-		
37900	Cytochrome P450 CYP2Y3 [*Danio rerio*]	AAX37329	6.47E-90	oxidation-reduction process	heme binding	-	-3.14	-2.61
				-	oxidoreductase activity, acting on paired donors, with incorporation or reduction of molecular oxygen, reduced flavin or flavoprotein as one donor, and incorporation of one atom of oxygen	-		
				-	iron ion binding	-		
				-	electron carrier activity	-		
44856	T-cell immunoglobulin and mucin domain-containing protein 4 precursor [*Salmo salar*]	ACN10511	2.34E-25	-	-	cell part	-3.09	-2.25
48697	Renalase [*Chelonia mydas*]	EMP24623	6.02E-31	N/A			-3.07	-3.19
41986	DNA-damage-inducible transcript 4 protein [*Dicentrarchus labrax*]	CBN81525	9.89E-88	neuron migration	14-3-3 protein binding	mitochondrion	-3.06	-1.89
				negative regulation of glycolysis	-	-		
				neurotrophin TRK receptor signaling pathway	-	-		
				negative regulation of intracellular protein kinase cascade	-	-		
				positive regulation of neuron death	-	-		
				cell proliferation	-	-		
				brain development	-	-		
				response to hypoxia	-	-		
				negative regulation of TOR signaling cascade	-	-		
				reactive oxygen species metabolic process	-	-		
				protein complex disassembly	-	-		
				intrinsic apoptotic signaling pathway in response to DNA damage by p53 class mediator	-	-		
				negative regulation of peptidyl-threonine phosphorylation	-	-		

				negative regulation of peptidyl-serine phosphorylation	-	-		
				neuron differentiation	-	-		
37117	Eukaryotic translation initiation factor 4E [*Osmerus mordax*]	ACO08860	3.08E-121	translational initiation	translation initiation factor activity	mRNA cap binding complex	-3.05	-4.11
				cytokine-mediated signaling pathway	-	cytosol		
				regulation of translation	-	-		
53601	No significant blastx hit			N/A			-2.94	-1.82
54710	No significant blastx hit			N/A			-2.48	-2.49
49314	No significant blastx hit			N/A			-2.47	-1.64
46572	PREDICTED: synaptic vesicle glycoprotein 2B-like [*Poecilia reticulata*]	XP_008409421	4.14E-18	N/A			-2.42	-3.25
50424	No significant blastx hit			N/A			-2.42	-1.94
49265	No significant blastx hit			N/A			-2.37	-2.24
47220	No significant blastx hit			N/A			-2.36	-1.87
42755	SLC6A19 [*Dicentrarchus labrax*]	AGL33763	2.02E-94	amino acid transmembrane transport	neutral amino acid transmembrane transporter activity	integral to plasma membrane	-2.31	-2.78
				neutral amino acid transport	neurotransmitter:sodiumsymporter activity	-		
				neurotransmitter transport	-	-		
				response to nutrient	-	-		
37207	Ferritin middle chain [*Oplegnathus fasciatus*]	BAM37460	5.37E-97	cellular iron ion homeostasis	ferric iron binding	-	-2.27	-1.89
				iron ion transport	ferroxidase activity	-		
				oxidation-reduction process	-	-		
37201	Ferritin, middle subunit [*Salmo salar*]	ACI66864	2.69E-12	cellular iron ion homeostasis	ferric iron binding	-	-2.25	-1.86
				iron ion transport	ferroxidase activity	-		
				oxidation-reduction process	-	-		
53338	No significant blastx hit			N/A			-2.20	-1.96
37397	Glutathione S-transferase pi [*Danio rerio*]	NP_571809	7.78E-99	negative regulation of leukocyte proliferation	glutathione binding	cytosol	-2.19	-2.28
				xenobiotic metabolic process	drug binding	plasma membrane		
				negative regulation of interleukin-1 beta production	dinitrosyl-iron complex binding	TRAF2-GSTP1 complex		
				positive regulation of superoxide anion generation	nitric oxide binding	nucleus		
				oligodendrocyte development	JUN kinase binding	-		
				cellular response to epidermal growth factor stimulus	S-nitrosoglutathione binding	-		
				negative regulation of tumor necrosis factor production	kinase regulator activity	-		
				negative regulation of stress-activated MAPK cascade	glutathione transferase activity	-		
				common myeloid progenitor cell proliferation	-	-		
				glutathione metabolic process	-	-		
				organ regeneration	-	-		
				response to ethanol	-	-		
				negative regulation of ERK1 and ERK2 cascade	-	-		
				negative regulation of I-kappaB kinase/NF-kappaB cascade	-	-		
				response to amino acid stimulus	-	-		
				negative regulation of JUN kinase activity	-	-		
				cellular response to lipopolysaccharide	-	-		

				cellular response to insulin stimulus	-	-		
				response to L-ascorbic acid	-	-		
				negative regulation of nitric-oxide synthase biosynthetic process	-	-		
				response to reactive oxygen species	-	-		
				negative regulation of acute inflammatory response	-	-		
				cellular response to glucocorticoid stimulus	-	-		
				negative regulation of tumor necrosis factor-mediated signaling pathway	-	-		
				nitric oxide storage	-	-		
				negative regulation of necrotic cell death	-	-		
				response to estradiol stimulus	-	-		
				response to toxic substance	-	-		
				cellular response to cell-matrix adhesion	-	-		
				response to mercury ion	-	-		
				negative regulation of apoptotic process	-	-		
				negative regulation of fibroblast proliferation	-	-		
				negative regulation of peroxidase activity	-	-		
				negative regulation of monocyte chemotactic protein-1 production	-	-		
37194	High affinity immunoglobulin epsilon receptor subunit gamma precursor [*Esox lucius*]	ACO13538	4.09E-23	neutrophil chemotaxis	IgG binding	membrane raft	-2.15	-1.85
				positive regulation of interleukin-6 production	IgE binding	Fc-epsilon receptor I complex		
				positive regulation of type I hypersensitivity	IgE receptor activity	integral to plasma membrane		
				regulation of platelet activation	-	external side of plasma membrane		
				positive regulation of type III hypersensitivity	-	-		
				negative regulation of mast cell apoptotic process	-	-		
				positive regulation of mast cell cytokine production	-	-		
				positive regulation of interleukin-10 production	-	-		
				phagocytosis, engulfment	-	-		
				positive regulation of type IIa hypersensitivity	-	-		
				antigen processing and presentation of exogenous peptide antigen via MHC class I	-	-		
				defense response to bacterium	-	-		
				neutrophil activation involved in immune response	-	-		
				serotonin secretion by platelet	-	-		
				antigen processing and presentation of exogenous peptide antigen via MHC class II	-	-		
				Fc receptor mediated stimulatory signaling pathway	-	-		
				Fc-epsilon receptor signaling pathway	-	-		
				positive regulation of phagocytosis	-	-		
				integrin-mediated signaling pathway	-	-		

				positive regulation of tumor necrosis factor production	-	-		
				positive regulation of mast cell degranulation	-	-		
41216	Nuclear protein 1 [*Salmo salar*]	ACI66304	3.80E-31	response to stimulus	-	-	-2.14	-1.62
				organ development	-	-		
				regulation of cellular process	-	-		
50497	No significant blastx hit			N/A			-2.11	-1.71
36522	TPA_exp: claudin 15 [*Salmo salar*]	DAA06160	1.04E-29	tube formation	structural molecule activity	integral to membrane	-2.07	-1.88
				digestive tract morphogenesis	-	tight junction		
				-	-	plasma membrane		
52639	No significant blastx hit			N/A			-2.06	-1.66
39283	Peroxiredoxin-1 [*Anoplopoma fimbria*]	ACQ58049	3.73E-125	skeletal system development	heme binding	mitochondrial matrix	-2.05	-2.51
				regulation of stress-activated MAPK cascade	thioredoxin peroxidase activity	nuclear euchromatin		
				hydrogen peroxide catabolic process	protein homodimerization activity	melanosome		
				erythrocyte homeostasis	-	nucleolus		
				natural killer cell mediated cytotoxicity	-	cytosol		
				cell proliferation	-	peroxisomal matrix		
				removal of superoxide radicals	-	-		
				regulation of NF-kappaB import into nucleus	-	-		
				oxidation-reduction process	-	-		
48620	No significant blastx hit			N/A			-2.03	-1.80
42540	Thioredoxin-interacting protein [*Salmo salar*]	ACN10667	1.80E-92	negative regulation of multicellular organismal metabolic process	ubiquitin protein ligase binding	mitochondrial intermembrane space	-2.02	-2.10
				response to glucose stimulus	enzyme inhibitor activity	cytosol		
				negative regulation of transcription from RNA polymerase II promoter	-	nucleus		
				negative regulation of catalytic activity	-	-		
				regulation of cell proliferation	-	-		
				response to hydrogen peroxide	-	-		
				response to mechanical stimulus	-	-		
				response to calcium ion	-	-		
				nucleotide-binding domain, leucine rich repeat containing receptor signaling pathway	-	-		
				response to progesterone stimulus	-	-		
				positive regulation of apoptotic process	-	-		
				negative regulation of cell division	-	-		
				response to drug	-	-		
				platelet-derived growth factor receptor signaling pathway	-	-		
				fat pad development	-	-		
				keratinocyte differentiation	-	-		
				negative regulation of heat generation	-	-		
				cellular response to tumor cell	-	-		
				protein import into nucleus	-	-		
				innate immune response	-	-		
				response to estradiol stimulus	-	-		
				negative regulation of locomotion involved in locomotory behavior	-	-		
				positive regulation of adrenergic receptor signaling pathway	-	-		
51680	No significant blastx hit			N/A			-2.01	-1.49

51325	No significant blastx hit			N/A			-1.99	-1.64
36536	Coagulation factor V precursor [*Danio rerio*]	NP_001007209	6.24E-15	platelet activation	copper ion binding	platelet alpha granule lumen	-1.98	-2.04
				intracellular receptor signaling pathway	oxidoreductase activity	cell surface		
				oxidation-reduction process	protein binding	extracellular region		
				negative regulation of cell growth	-	integral to plasma membrane		
				response to bacterium	-	-		
				cell adhesion	-	-		
				platelet degranulation	-	-		
38154	Ependymin-1 precursor [*Anoplopoma fimbria*]	ACQ58356	1.14E-41	N/A			-1.98	-1.69
41963	RNA-binding protein 5 [*Dicentrarchus labrax*]	CBN80582	1.45E-39	spliceosomal complex assembly	mRNA binding	spliceosomal complex	-1.97	-1.65
				positive regulation of apoptotic process	zinc ion binding	nucleolus		
				negative regulation of cell proliferation	DNA binding	nucleoplasm		
				regulation of alternative mRNA splicing, via spliceosome	protein binding	-		
				-	nucleotide binding	-		
41321	Probable ATP-dependent RNA helicase DDX41 [*Danio rerio*]	NP_957339	2.93E-104	multicellular organismal development	protein binding	nucleolus	-1.96	-1.81
				defense response to virus	zinc ion binding	catalytic step 2 spliceosome		
				apoptotic process	ATP-dependent helicase activity	endoplasmic reticulum		
				cellular response to interferon-beta	DNA binding	-		
				positive regulation of transcription from RNA polymerase II promoter	RNA binding	-		
				mRNA splicing, via spliceosome	ATP binding	-		
37609	Hepcidin precursor [*Gadus morhua*]	ACA42770	5.32E-37	response to bacterium	-	-	-1.94	-2.44
50272	No significant blastx hit			N/A			-1.92	-1.71
45407	Putative MFS-type transporter [*Cricetulus griseus*]	ERE83697	4.07E-28	N/A			-1.92	-2.08
38166	Microsomal glutathione S-transferase 3 [*Oncorhynchus mykiss*]	ACO07847	1.25E-73	response to organonitrogen compound	glutathione transferase activity	endoplasmic reticulum	-1.88	-1.99
				glutathione biosynthetic process	glutathione peroxidase activity	-		
54501	No significant blastx hit			N/A			-1.86	-1.92
50198	No significant blastx hit			N/A			-1.85	-1.60
42481	Solute carrier family 6, member 6 [*Salmo salar*]	NP_001117102	1.53E-104	amino acid transmembrane transport	taurine:sodiumsymporter activity	integral to plasma membrane	-1.84	-1.77
				neurotransmitter transport	beta-alanine transmembrane transporter activity	-		
				taurine transport	taurine binding	-		
				cellular amino acid metabolic process	neurotransmitter:sodiumsymporter activity	-		
				beta-alanine transport	-	-		
39084	No significant blastx hit			N/A			-1.83	-1.75
53958	No significant blastx hit			N/A			-1.80	-1.58
54703	No significant blastx hit			N/A			-1.80	-1.97
52998	No significant blastx hit			N/A			-1.79	-1.63
35893	Aminopeptidase N [*Thunnus thynnus*]	CAX33862	2.36E-55	angiogenesis	zinc ion binding	vesicle membrane	-1.75	-1.36
				regulation of systemic arterial blood pressure by renin-angiotensin	peptide binding	endoplasmic reticulum-Golgi intermediate compartment		
				negative regulation of renal sodium excretion	receptor activity	external side of plasma membrane		
				cell proliferation	metalloaminopeptidase activity	apical part of cell		

				cell differentiation	-	integral to plasma membrane		
				cellular aromatic compound metabolic process	-	vesicle lumen		
				proteolysis	-	-		
				cell migration	-	-		
51975	No significant blastx hit			N/A			-1.75	-1.49
54100	No significant blastx hit			N/A			-1.75	-1.70
45123	Solute carrier family 2, facilitated glucose transporter member 11 [*Dicentrarchus labrax*]	CBN81089	3.40E-51	hexose transmembrane transport	glucose transmembrane transporter activity	integral to plasma membrane	-1.74	-1.46
				glucose transport	-	nuclear envelope		
				urate metabolic process	-	-		
52430	No significant blastx hit			N/A			-1.72	-1.42
55219	No significant blastx hit			N/A			-1.72	-1.64
51438	No significant blastx hit			N/A			-1.72	-1.70
52781	No significant blastx hit			N/A			-1.72	-1.81
36142	Beta2-microglobulin [*Gadus morhua*]	CAA10762	7.04E-57	N/A			-1.72	-1.74
42318	SLC6A19 [*Dicentrarchus labrax*]	AGL33763	1.04E-113	amino acid transmembrane transport	neutral amino acid transmembrane transporter activity	integral to plasma membrane	-1.71	-1.53
				neutral amino acid transport	neurotransmitter:sodiumsymporter activity	-		
				neurotransmitter transport	-	-		
				response to nutrient	-	-		
51681	No significant blastx hit			N/A			-1.70	-2.02
38972	No significant blastx hit			N/A			-1.69	-1.56
52730	No significant blastx hit			N/A			-1.68	-1.66
52763	No significant blastx hit			N/A			-1.67	-1.44
49979	No significant blastx hit			N/A			-1.65	-1.50
42067	Sequestosome-1 [*Pteropus alecto*]	ELK03198	3.57E-18	positive regulation of protein phosphorylation	protein kinase C binding	nucleoplasm	-1.65	-1.46
				protein heterooligomerization	zinc ion binding	cytosol		
				neurotrophin TRK receptor signaling pathway	transcription cofactor activity	late endosome		
				macroautophagy	receptor tyrosine kinase binding	pre-autophagosomal structure		
				regulation of Ras protein signal transduction	K63-linked polyubiquitin binding	endoplasmic reticulum		
				apoptotic process	SH2 domain binding	-		
				endosomal transport	protein serine/threonine kinase activity	-		
				ubiquitin-dependent protein catabolic process	protein homodimerization activity	-		
				protein localization	-	-		
				cell differentiation	-	-		
				positive regulation of transcription from RNA polymerase II promoter	-	-		
				regulation of I-kappaB kinase/NF-kappaB cascade	-	-		
38461	DEAD (Asp-Glu-Ala-Asp) box polypeptide 21 [*Salmo salar*]	ACH85363	1.46E-37	response to exogenous dsRNA	double-stranded RNA binding	nucleolus	-1.64	-1.47
				response to virus	ATP-dependent helicase activity	plasma membrane		
				-	DNA binding	-		
				-	ATP binding	-		
52364	No significant blastx hit			N/A			-1.64	-1.44
49188	No significant blastx hit			N/A			-1.63	-1.60

51062	No significant blastx hit			N/A			-1.63	-1.84
47474	WSC domain-containing protein 2, partial [*Columba livia*]	EMC86879	5.75E-10	-	sulfotransferase activity	-	-1.63	-1.30
48418	No significant blastx hit			N/A			-1.62	-1.56
39218	Myosin light chain 2 [*Gadus chalcogrammus*]	BAB18578	3.45E-88	-	calcium ion binding	myosin complex	-1.61	-1.32
35888	Alpha-1-microglobulin [*Ctenopharyngodon idella*]	ABW37741	3.30E-43	regulation of biological process	protein binding	intracellular membrane-bounded organelle	-1.61	-1.31
				protein catabolic process	serine-type endopeptidase inhibitor activity	extracellular space		
				cellular process	-	cell surface		
54616	No significant blastx hit			N/A			-1.60	-1.39
38478	Cytochrome P450 3A [*Scophthalmus maximus*]	AEV42266	0.00E+00	response to xenobiotic stimulus	metal ion binding	organelle membrane	-1.60	-1.52
				-	oxidoreductase activity, acting on paired donors, with incorporation or reduction of molecular oxygen, reduced flavin or flavoprotein as one donor, and incorporation of one atom of oxygen	endoplasmic reticulum		
49726	No significant blastx hit			N/A			-1.60	-1.42
36829	Dehydrogenase/reductase SDR family member 1 [*Salmo salar*]	NP_001134326	1.87E-59	oxidation-reduction process	oxidoreductase activity	mitochondrial inner membrane	-1.60	-2.29
				-	nucleotide binding	endoplasmic reticulum		
54091	No significant blastx hit			N/A			-1.59	-1.67
36141	Beta2-microglobulin [*Gadusmorhua*]	CAA10762	7.68E-58	N/A			-1.59	-1.69
37115	Eukaryotic translation initiation factor 4E-binding protein 2 [*Cyprinus carpio 'jian'*]	ADN92457	5.24E-56	lung development	translation repressor activity	cytoplasm	-1.59	-1.42
				cAMP-mediated signaling	translation initiation factor activity	nucleolus		
				insulin receptor signaling pathway	eukaryotic initiation factor 4E binding	protein complex		
				response to ethanol	-	-		
				positive regulation of mitotic cell cycle	-	-		
				negative regulation of translational initiation	-	-		
				TOR signaling cascade	-	-		
				negative regulation of protein complex assembly	-	-		
				G1/S transition of mitotic cell cycle	-	-		
37719	Glutamate-cysteine ligase catalytic subunit, partial [*Oryzias melastigma*]	AEZ55098	5.34E-110	regulation of mitochondrial depolarization	ADP binding	cytosol	-1.58	-1.45
				response to nitrosative stress	protein heterodimerization activity	glutamate-cysteine ligase complex		
				L-ascorbic acid metabolic process	glutamate binding	-		
				response to arsenic-containing substance	coenzyme binding	-		
				negative regulation of transcription, DNA-dependent	magnesium ion binding	-		
				positive regulation of proteasomal ubiquitin-dependent protein catabolic process	ATP binding	-		
				cysteine metabolic process	glutamate-cysteine ligase activity	-		
				cell redox homeostasis	-	-		
				xenobiotic metabolic process	-	-		
				response to heat	-	-		
				glutamate metabolic process	-	-		

				negative regulation of protein ubiquitination	-	-		
				negative regulation of neuron apoptotic process	-	-		
				glutathione biosynthetic process	-	-		
				response to hormone stimulus	-	-		
				regulation of blood vessel size	-	-		
				response to oxidative stress	-	-		
45770	No significant blastx hit			N/A			-1.58	-1.44
38012	Carbonic anhydrase 6 [*Columba livia*]	EMC79327	1.09E-81	one-carbon metabolic process	carbonate dehydratase activity	cytoplasm	-1.58	-1.52
				bicarbonate transport	zinc ion binding	extracellular space		
50732	No significant blastx hit			N/A			-1.57	-1.64
52891	No significant blastx hit			N/A			-1.57	-1.67
52943	No significant blastx hit			N/A			-1.56	-1.52
51178	No significant blastx hit			N/A			-1.56	-1.36
49795	No significant blastx hit			N/A			-1.56	-1.44
52056	No significant blastx hit			N/A			-1.54	-1.33
46236	SH3 domain-binding glutamic acid-rich-like protein 3 [*Esox lucius*]	ACO14404	4.63E-19	-	-	cytoplasm	-1.53	-1.49
51345	No significant blastx hit			N/A			-1.53	-1.45
44066	Omega class glutathione S-transferase [*Oplegnathus fasciatus*]	ADY80021	5.96E-117	L-ascorbic acid metabolic process	glutathione transferase activity	cytosol	-1.52	-1.63
				xenobiotic catabolic process	glutathione dehydrogenase (ascorbate) activity	-		
				cellular response to arsenic-containing substance	-	-		
43915	AN1-type zinc finger protein 2B [*Danio rerio*]	NP_956811	1.67E-92	-	zinc ion binding	endoplasmic reticulum	-1.52	-1.47
				-	protein binding	-		
37065	Epididymal secretory protein E1 precursor [*Osmerus mordax*]	ACO09051	1.09E-79	response to virus	enzyme binding	lysosome	-1.52	-1.61
				cholesterol efflux	cholesterol binding	extracellular region		
				regulation of isoprenoid metabolic process	-	endoplasmic reticulum		
				glycolipid transport	-	-		
				intracellular cholesterol transport	-	-		
				phospholipid transport	-	-		
				cholesterol homeostasis	-	-		
				cholesterol metabolic process	-	-		
42541	Thioredoxin interacting protein a [*Danio rerio*]	NP_956381	6.90E-106	response to glucose stimulus	ubiquitin protein ligase binding	mitochondrial intermembrane space	-1.52	-1.93
				negative regulation of transcription from RNA polymerase II promoter	enzyme inhibitor activity	cytosol		
				negative regulation of catalytic activity	-	nucleus		
				regulation of cell proliferation	-	-		
				response to hydrogen peroxide	-	-		
				response to mechanical stimulus	-	-		
				response to calcium ion	-	-		
				nucleotide-binding domain, leucine rich repeat containing receptor signaling pathway	-	-		
				response to progesterone stimulus	-	-		
				positive regulation of apoptotic process	-	-		
				negative regulation of cell division	-	-		
				response to drug	-	-		
				platelet-derived growth factor receptor signaling pathway	-	-		

				keratinocyte differentiation	-	-		
				cellular response to tumor cell	-	-		
				protein import into nucleus	-	-		
				innate immune response	-	-		
				response to estradiol stimulus	-	-		
				cell cycle	-	-		
36389	CCAAT/enhancer-binding protein delta [*Salmo salar*]	ACM09181	1.41E-47	mammary gland epithelial cell differentiation	RNA polymerase II core promoter proximal region sequence-specific DNA binding transcription factor activity involved in positive regulation of transcription	cytoplasm	-1.51	-1.62
				response to lipopolysaccharide	protein heterodimerization activity	nuclear matrix		
				mammary gland epithelial cell proliferation	transcription regulatory region DNA binding	nuclear chromatin		
				negative regulation of apoptotic process	RNA polymerase II distal enhancer sequence-specific DNA binding transcription factor activity	-		
				embryonic placenta development	sequence-specific DNA binding	-		
				brown fat cell differentiation	protein homodimerization activity	-		
				immune response	glucocorticoid receptor binding	-		
				negative regulation of transcription, DNA-dependent	transcription factor binding	-		
				regulation of interleukin-6 biosynthetic process	-	-		
				acute-phase response	-	-		
				response to endoplasmic reticulum stress	-	-		
				inner ear development	-	-		
				apoptotic process	-	-		
				positive regulation of transcription from RNA polymerase II promoter	-	-		
				positive regulation of osteoblast differentiation	-	-		
				cellular response to amino acid stimulus	-	-		
				neuron differentiation		-		
48816	No significant blastx hit			N/A			-1.51	-1.44
45427	Dehydrogenase/reductase SDR family member 12 [*Salmo salar*]	ACI69912	4.56E-127	oxidation-reduction process	sequence-specific DNA binding transcription factor activity	-	-1.50	-1.39
				regulation of transcription, DNA-dependent	oxidoreductase activity	-		
				-	nucleotide binding	-		
53417	No significant blastx hit			N/A			-1.50	-1.33
48777	No significant blastx hit			N/A			-1.50	-1.47
36745	Cytochrome b5 [*Coryphaenoides armatus*]	CAE75863	6.80E-56	L-ascorbic acid metabolic process	stearoyl-CoA 9-desaturase activity	mitochondrial outer membrane	-1.50	-1.40
				fatty acid metabolic process	cytochrome-c oxidase activity	endoplasmic reticulum membrane		
				oxidation-reduction process	metal ion binding	-		
				response to cadmium ion	electron carrier activity	-		
				-	enzyme binding	-		
				-	heme binding	-		
				-	aldo-ketoreductase (NADP) activity	-		
54843	No significant blastx hit			N/A			-1.50	-1.44
44061	Short chain dehydrogenase reductase [*Sparus aurata*]	ACD80264	1.03E-78	metabolic process	-	-	-1.49	-1.36
51068	No significant blastx hit			N/A			-1.49	-1.41
38487	Pentraxin [*Gadus morhua*]	ACZ06557	4.34E-108	N/A			-1.49	-1.33
50886	No significant blastx hit			N/A			-1.49	-1.39

48351	No significant blastx hit			N/A			-1.49	-1.39
37711	Flavin containing monooxygenase 5 [*Danio rerio*]	CAI21028	3.83E-71	oxidation-reduction process	flavin adenine dinucleotide binding	intrinsic to endoplasmic reticulum membrane	-1.48	-1.42
				-	NADP binding	integral to membrane		
				-	N,N-dimethylanilinemonooxygenase activity	-		
42825	PREDICTED: tubulin polymerization-promoting protein family member 3-like [*Stegastes partitus*]	XP_008303281	6.36E-17	microtubule bundle formation	tubulin binding	cytoplasm	-1.47	-1.25
				-	calcium ion binding	microtubule		
35941	Antithrombin protein precursor [*Salmo salar*]	NP_001117104	4.54E-101	oxidation-reduction process	serine-type endopeptidase inhibitor activity	extracellular space	-1.47	-1.34
				response to nutrient	protease binding	ribonucleoside-diphosphatereductase complex		
				negative regulation of endopeptidase activity	ribonucleoside-diphosphatereductase activity, thioredoxin disulfide as acceptor	plasma membrane		
				DNA replication	heparin binding	-		
				regulation of proteolysis	-	-		
				blood coagulation	-	-		
				negative regulation of inflammatory response	-	-		
53843	No significant blastx hit			N/A			-1.46	-1.56
36002	Clec10a protein [*Danio rerio*]	AAI48181	2.51E-29	positive regulation of natural killer cell mediated cytotoxicity	-	membrane	-1.45	-1.56
				regulation of protein stability	-	-		
				lipid homeostasis	-	-		
				glycoprotein metabolic process	-	-		
50385	No significant blastx hit			N/A			-1.45	-1.42
54635	No significant blastx hit			N/A			-1.44	-1.39
40257	Beta-microseminoprotein precursor [*Esox lucius*]	ACO13654	2.08E-16	N/A			-1.44	-1.45
50537	No significant blastx hit			N/A			-1.43	-1.31
37394	Glutathione peroxidase 6 [*Dicentrarchus labrax*]	CBN81531	1.31E-76	hydrogen peroxide catabolic process	glutathione peroxidase activity	extracellular space	-1.43	-1.30
				response to lipid hydroperoxide	transcription factor binding	-		
				protein homotetramerization	selenium binding	-		
54377	No significant blastx hit			N/A			-1.43	-1.45
49550	No significant blastx hit			N/A			-1.42	-1.41
49400	No significant blastx hit			N/A			-1.42	-1.29
42207	Folate receptor alpha precursor [*Anoplopoma fimbria*]	ACQ58933	3.12E-101	transmembrane transport	folic acid transporter activity	extrinsic to membrane	-1.42	-1.43
				folic acid metabolic process	folic acid binding	brush border		
				folic acid transport	receptor activity	anchored to plasma membrane		
				posttranslational protein targeting to membrane	-	-		
37198	High affinity immunoglobulin epsilon receptor subunit gamma precursor [*Salmo salar*]	ACI69533	3.16E-17	neutrophil chemotaxis	IgG binding	Fc-epsilon receptor I complex	-1.42	-1.42
				positive regulation of interleukin-6 production	IgE binding	integral to plasma membrane		
				positive regulation of type I hypersensitivity	IgE receptor activity	external side of plasma membrane		
				regulation of platelet activation	-	-		

				positive regulation of type III hypersensitivity	-	-		
				negative regulation of mast cell apoptotic process	-	-		
				positive regulation of mast cell cytokine production	-	-		
				positive regulation of interleukin-10 production	-	-		
				phagocytosis, engulfment	-	-		
				positive regulation of type IIa hypersensitivity	-	-		
				antigen processing and presentation of exogenous peptide antigen via MHC class I	-	-		
				defense response to bacterium	-	-		
				neutrophil activation involved in immune response	-	-		
				serotonin secretion by platelet	-	-		
				antigen processing and presentation of exogenous peptide antigen via MHC class II	-	-		
				Fc receptor mediated stimulatory signaling pathway	-	-		
				Fc-epsilon receptor signaling pathway	-	-		
				positive regulation of phagocytosis	-	-		
				integrin-mediated signaling pathway	-	-		
				positive regulation of tumor necrosis factor production	-	-		
				positive regulation of mast cell degranulation	-	-		
42465	Transaldolase [*Osmerus mordax*]	ACO08950	2.68E-151	xylulose biosynthetic process	monosaccharide binding	cytosol	-1.41	-1.50
				pentose-phosphate shunt, non-oxidative branch	sedoheptulose-7-phosphate:D-glyceraldehyde-3-phosphate glyceronetransferase activity	intracellular membrane-bounded organelle		
				glyceraldehyde-3-phosphate metabolic process	-	-		
				fructose 6-phosphate metabolic process	-	-		
				energy reserve metabolic process	-	-		
43593	Cytosolic Fe-S cluster assembly factor nubp2 [*Dicentrarchus labrax*]	CBN81315	3.00E-108	-	metal ion binding	microtubule organizing center	-1.40	-1.45
				-	protein binding	nucleus		
				-	ATP binding	-		
				-	4 iron, 4 sulfur cluster binding	-		
				-	nucleoside-triphosphatase activity	-		
51838	No significant blastx hit			N/A			-1.40	-1.45
51555	PREDICTED: tripartite motif-containing protein 44-like [*Xiphophorus maculatus*]	XP_005812057	1.01E-11	N/A			-1.39	-1.32
53629	No significant blastx hit			N/A			-1.39	-1.31
41215	Serine/threonine-protein kinase WNK4 [*Tupaia chinensis*]	ELW54714	3.36E-06	-	transferase activity, transferring phosphorus-containing groups	-	-1.39	-1.46
37619	Homogentisate 1,2-dioxygenase [*Dicentrarchus labrax*]	CBN81879	3.58E-107	tyrosine catabolic process	nutrient reservoir activity	cytosol	-1.39	-1.46
				cellular nitrogen compound metabolic process	metal ion binding	-		
				L-phenylalanine catabolic process	homogentisate 1,2-dioxygenase activity	-		
				oxidation-reduction process	-	-		

51316	No significant blastx hit			N/A			-1.39	-1.40
52139	No significant blastx hit			N/A			-1.38	-1.29
42392	Methylsterol monooxygenase 1 [*Xenopus (Silurana) tropicalis*]	NP_001072809	1.88E-32	fatty acid biosynthetic process	C-4 methylsterol oxidase activity	integral to membrane	-1.38	-1.50
				oxidation-reduction process	iron ion binding	endoplasmic reticulum membrane		
				cholesterol biosynthetic process	-	plasma membrane		
36350	Catalase [*Oreochromis niloticus*]	AEE40963	4.82E-11	response to copper ion	metal ion binding	peroxisome	-1.38	-1.49
				hydrogen peroxide catabolic process	heme binding	-		
				oxidation-reduction process	catalase activity	-		
43452	Peroxisomal carnitine O-octanoyltransferase [*Danio rerio*]	NP_001018161	3.47E-21	response to organonitrogen compound	receptor binding	mitochondrion	-1.38	-1.23
				medium-chain fatty acid metabolic process	carnitine O-octanoyltransferase activity	peroxisomal matrix		
				fatty acid beta-oxidation using acyl-CoA oxidase	-	-		
				generation of precursor metabolites and energy	-	-		
				response to drug	-	-		
				medium-chain fatty acid transport	-	-		
				long-chain fatty acid transport	-	-		
47925	PREDICTED: arginine/serine-rich protein 1 [*Stegastes partitus*]	XP_008282446	3.32E-15	N/A			-1.38	-1.41
43684	Adrenodoxin-like protein, mitochondrial precursor [*Danio rerio*]	NP_001070132	8.01E-78	transport	metal ion binding	mitochondrion	-1.37	-1.35
				electron transport chain	electron carrier activity	-		
				-	2 iron, 2 sulfur cluster binding	-		
36315	Carnitine octanoyltransferase [*Takifugu rubripes*]	AAO20903	3.54E-123	response to organonitrogen compound	receptor binding	mitochondrion	-1.37	-1.30
				medium-chain fatty acid metabolic process	carnitine O-octanoyltransferase activity	peroxisomal matrix		
				fatty acid beta-oxidation using acyl-CoA oxidase	-	-		
				generation of precursor metabolites and energy	-	-		
				response to drug	-	-		
				medium-chain fatty acid transport	-	-		
				long-chain fatty acid transport	-	-		
48550	PREDICTED: asialoglycoprotein receptor 1-like [*Stegastes partitus*]	XP_008298537	1.94E-15	N/A			-1.36	-1.28
54516	No significant blastx hit			N/A			-1.36	-1.22
50233	No significant blastx hit			N/A			-1.36	-1.36
48043	No significant blastx hit			N/A			-1.36	-1.46
53344	No significant blastx hit			N/A			-1.35	-1.28
39641	Calcium channel, voltage-dependent, gamma subunit 8a [*Danio rerio*]	NP_001017733	1.83E-08	synaptic transmission	voltage-gated calcium channel activity	endocytic vesicle membrane	-1.35	-1.49
				calcium ion transport	-	alpha-amino-3-hydroxy-5-methyl-4-isoxazolepropionic acid selective glutamate receptor complex		
				regulation of alpha-amino-3-hydroxy-5-methyl-4-isoxazole propionate selective glutamate receptor activity	-	voltage-gated calcium channel complex		
				-	-	postsynaptic density		
36458	Ceruloplasmin [*Chionodraco rastrospinosus*]	CAL92184	1.70E-114	lung development	copper ion binding	extracellular space	-1.34	-1.31

				oxidation-reduction process	chaperone binding	anchored to plasma membrane		
				liver development	ferroxidase activity	-		
				response to nutrient	-	-		
				cellular iron ion homeostasis	-	-		
				plasma membrane copper ion transport	-	-		
				response to copper ion	-	-		
				aging	-	-		
37932	Parvalbumin, thymic [*Salmo salar*]	NP_001134150	1.62E-28	-	calcium ion binding	axon	-1.34	-1.29
				-	-	cytoplasm		
38462	Nucleolar RNA helicase 2 [*Heterocephalus glaber*]	EHB15506	3.24E-32	response to virus	ATP-dependent RNA helicase activity	nucleolus	-1.33	-1.36
				rRNA processing	DNA binding	plasma membrane		
				response to exogenous dsRNA	double-stranded RNA binding	-		
				-	ATP binding	-		
				-	protein binding	-		
36140	Beta2-microglobulin [*Gadus morhua*]	CAA10762	1.43E-12	antigen processing and presentation of peptide antigen via MHC class I	-	extracellular region	-1.33	-1.56
				-	-	MHC class I protein complex		
46935	No significant blastx hit			N/A			-1.32	-1.33
37870	Mid1-interacting protein 1 [*Epinephelus coioides*]	AEG78348	6.17E-54	negative regulation of microtubule depolymerization	protein C-terminus binding	cytosol	-1.30	-1.27
				positive regulation of fatty acid biosynthetic process	-	microtubule		
				positive regulation of ligase activity	-	nucleus		
				protein polymerization	-	-		
45640	No significant blastx hit			N/A			-1.29	-1.33
52693	No significant blastx hit			N/A			-1.28	-1.35
49157	No significant blastx hit			N/A			-1.27	-1.38
54365	No significant blastx hit			N/A			-1.27	-1.39
44792	Proteinase-activated receptor-2a precursor [*Salmo salar*]	NP_001136193	8.80E-82	establishment of endothelial barrier	heterotrimeric G-protein binding	integral to plasma membrane	-1.26	-1.40
				elevation of cytosolic calcium ion concentration	peptidase activity	Golgi apparatus		
				negative regulation of JNK cascade	receptor binding	pseudopodium		
				positive regulation of I-kappaB kinase/NF-kappaB cascade	thrombin receptor activity	-		
				mature dendritic cell differentiation	G-protein beta-subunit binding	-		
				positive regulation of actin filament depolymerization	G-protein alpha-subunit binding	-		
				thrombin receptor signaling pathway	-	-		
				T cell activation involved in immune response	-	-		
				positive regulation of toll-like receptor 4 signaling pathway	-	-		
				positive regulation of ERK1 and ERK2 cascade	-	-		
				positive regulation of renin secretion into blood stream	-	-		
				positive regulation of superoxide anion generation	-	-		
				positive regulation of cytokine secretion involved in immune response	-	-		
				neutrophil activation	-	-		

				positive regulation of glomerular filtration	-	-		
				positive regulation of pseudopodium assembly	-	-		
				positive regulation of eosinophil degranulation	-	-		
				interleukin-1 beta secretion	-	-		
				negative regulation of toll-like receptor 3 signaling pathway	-	-		
				defense response to virus	-	-		
				proteolysis	-	-		
				positive regulation of toll-like receptor 2 signaling pathway	-	-		
				positive regulation of Rho protein signal transduction	-	-		
				interferon-gamma secretion	-	-		
				positive regulation of transcription from RNA polymerase II promoter	-	-		
				interleukin-10 secretion	-	-		
				negative regulation of chemokine secretion	-	-		
				positive regulation of interleukin-8 secretion	-	-		
				positive regulation of leukocyte chemotaxis	-	-		
				leukocyte proliferation	-	-		
				positive regulation of neutrophil mediated killing of gram-negative bacterium	-	-		
				positive regulation of phagocytosis, engulfment	-	-		
				negative regulation of tumor necrosis factor-mediated signaling pathway	-	-		
				regulation of blood coagulation	-	-		
				chemokine (C-C motif) ligand 2 secretion	-	-		
				positive regulation of JNK cascade	-	-		
				positive regulation of toll-like receptor 3 signaling pathway	-	-		
				positive regulation of vasodilation	-	-		
				positive regulation of interleukin-6 secretion	-	-		
				positive regulation of phosphatidylinositol 3-kinase cascade	-	-		
				positive regulation of positive chemotaxis	-	-		
49841	No significant blastx hit			N/A			-1.26	-1.28
49324	No significant blastx hit			N/A			-1.24	-1.24
44712	No significant blastx hit			N/A			-1.22	-1.27
50025	No significant blastx hit			N/A			-1.20	-1.34
43903	Bloodthirsty-related gene family, member 30 [*Danio rerio*]	NP_956716	9.75E-71	-	protein binding	intracellular	-1.18	-1.24
				-	zinc ion binding	integral to membrane		

[1]Probe identifier (ID) numbers are 5-digit unique identifiers for the 50mer probes on the Atlantic cod 20K microarray (Booman et al. 2011).
[2]The BLASTx hit with the lowest E-value and a protein name (e.g. not "unnamed protein product" or "predicted") is shown.
[3]GO terms in this table were collected using Blast2GO (see Methods).
