# Supplementary material for: Transcriptome profiling reveals that feeding wild zooplankton to larval Atlantic cod (Gadus morhua) influences suites of genes involved in oxidation-reduction, mitosis, and selenium homeostasis
Source: BMC Genomics. 2015 Nov 26;16:1016. doi: 10.1186/s12864-015-2120-1 (PMC4661974; doi:10.1186/s12864-015-2120-1)
Supplement: Additional file 3: Table S3. — Complete list of overrepresented and underrepresented GO terms in test set of genes (303 genes differentially expressed between RA-Zoo and both RA and RA-PH) compared with reference set of genes (20K cod microarray). (PDF 90kb) [file 12864_2015_2120_MOESM3_ESM.pdf]

Supplemental Table 3. Complete list of overrepresented and underrepresented GO terms in test set of genes (303 genes differentially expressed between RA-Zoo and both RA and RA-PH) compared with reference set of genes (20K cod microarray)

GO ID	GO Term	GO Category[1]	FDR	Over/Underrepresented
GO:0000780	condensed nuclear chromosome, centromeric region	C	3.47E-06	over
GO:0000940	condensed chromosome outer kinetochore	C	4.56E-06	over
GO:0000942	condensed nuclear chromosome outer kinetochore	C	1.08E-05	over
GO:0000778	condensed nuclear chromosome kinetochore	C	1.08E-05	over
GO:0000775	chromosome, centromeric region	C	1.08E-05	over
GO:0000776	kinetochore	C	1.44E-05	over
GO:0000779	condensed chromosome, centromeric region	C	2.24E-05	over
GO:0000777	condensed chromosome kinetochore	C	7.38E-05	over
GO:0000922	spindle pole	C	1.82E-03	over
GO:0000793	condensed chromosome	C	2.27E-03	over
GO:0005819	spindle	C	2.47E-03	over
GO:0000794	condensed nuclear chromosome	C	3.07E-03	over
GO:0044454	nuclear chromosome part	C	6.26E-03	over
GO:0030532	small nuclear ribonucleoprotein complex	C	1.43E-02	over
GO:0005683	U7 snRNP	C	1.94E-02	over
GO:0044427	chromosomal part	C	2.44E-02	over
GO:0032133	chromosome passenger complex	C	2.70E-02	over
GO:0000228	nuclear chromosome	C	2.76E-02	over
GO:0032998	Fc-epsilon receptor I complex	C	3.55E-02	over
GO:0032997	Fc receptor complex	C	3.55E-02	over
GO:0005694	chromosome	C	3.81E-02	over
GO:0016209	antioxidant activity	F	3.47E-06	over
GO:0016684	oxidoreductase activity, acting on peroxide as acceptor	F	1.17E-04	over
GO:0004601	peroxidase activity	F	1.17E-04	over
GO:0016491	oxidoreductase activity	F	1.31E-04	over
GO:0008430	selenium binding	F	1.08E-03	over
GO:0004602	glutathione peroxidase activity	F	1.29E-02	over
GO:0010997	anaphase-promoting complex binding	F	1.94E-02	over
GO:1900750	oligopeptide binding	F	2.70E-02	over
GO:0043295	glutathione binding	F	2.70E-02	over
GO:0008017	microtubule binding	F	3.55E-02	over
GO:0072341	modified amino acid binding	F	3.55E-02	over
GO:0019863	IgE binding	F	3.55E-02	over
GO:0019767	IgE receptor activity	F	3.55E-02	over
GO:0019763	immunoglobulin receptor activity	F	3.55E-02	over
GO:0015631	tubulin binding	F	3.71E-02	over
GO:0000236	mitotic prometaphase	P	1.08E-05	over
GO:0042744	hydrogen peroxide catabolic process	P	1.44E-05	over
GO:0051301	cell division	P	5.30E-05	over
GO:0000226	microtubule cytoskeleton organization	P	2.04E-04	over
GO:0042743	hydrogen peroxide metabolic process	P	7.09E-04	over
GO:0007017	microtubule-based process	P	1.13E-03	over
GO:0007067	mitosis	P	1.82E-03	over
GO:0000280	nuclear division	P	1.90E-03	over
GO:0072593	reactive oxygen species metabolic process	P	2.12E-03	over
GO:0042542	response to hydrogen peroxide	P	2.27E-03	over
GO:0034502	protein localization to chromosome	P	2.44E-03	over
GO:0048285	organelle fission	P	2.61E-03	over
GO:0070301	cellular response to hydrogen peroxide	P	2.62E-03	over
GO:0055114	oxidation-reduction process	P	4.08E-03	over
GO:0007051	spindle organization	P	6.22E-03	over
GO:0006749	glutathione metabolic process	P	7.85E-03	over
GO:0000302	response to reactive oxygen species	P	1.06E-02	over
GO:0045787	positive regulation of cell cycle	P	1.15E-02	over
GO:0008608	attachment of spindle microtubules to kinetochore	P	1.15E-02	over
GO:0010799	regulation of peptidyl-threonine phosphorylation	P	1.15E-02	over
GO:0031145	anaphase-promoting complex-dependent proteasomal ubiquitin-dependent protein catabolic process	P	1.25E-02	over
GO:0006982	response to lipid hydroperoxide	P	1.25E-02	over
GO:0040038	polar body extrusion after meiotic divisions	P	1.25E-02	over
GO:0090007	regulation of mitotic anaphase	P	1.25E-02	over
GO:0043302	positive regulation of leukocyte degranulation	P	1.25E-02	over
GO:0000088	mitotic prophase	P	1.25E-02	over
GO:0002888	positive regulation of myeloid leukocyte mediated immunity	P	1.25E-02	over
GO:0051488	activation of anaphase-promoting complex activity	P	1.29E-02	over
GO:0007052	mitotic spindle organization	P	1.43E-02	over
GO:0051785	positive regulation of nuclear division	P	1.62E-02	over
GO:0045840	positive regulation of mitosis	P	1.62E-02	over
GO:0006518	peptide metabolic process	P	1.68E-02	over
GO:1902099	regulation of metaphase/anaphase transition of cell cycle	P	1.71E-02	over
GO:0030071	regulation of mitotic metaphase/anaphase transition	P	1.71E-02	over
GO:0007092	activation of mitotic anaphase-promoting complex activity	P	1.94E-02	over
GO:0051983	regulation of chromosome segregation	P	1.94E-02	over
GO:0010269	response to selenium ion	P	2.43E-02	over
GO:0007143	female meiosis	P	2.43E-02	over
GO:0007091	metaphase/anaphase transition of mitotic cell cycle	P	2.48E-02	over
GO:0044784	metaphase/anaphase transition of cell cycle	P	2.48E-02	over
GO:0000086	G2/M transition of mitotic cell cycle	P	2.70E-02	over

GO:0007080	mitotic metaphase plate congression	P	2.70E-02	over
GO:0051310	metaphase plate congression	P	2.70E-02	over
GO:0051261	protein depolymerization	P	2.70E-02	over
GO:0034614	cellular response to reactive oxygen species	P	3.18E-02	over
GO:0000079	regulation of cyclin-dependent protein serine/threonine kinase activity	P	3.40E-02	over
GO:0007059	chromosome segregation	P	3.55E-02	over
GO:0006979	response to oxidative stress	P	3.55E-02	over
GO:0001812	positive regulation of type I hypersensitivity	P	3.55E-02	over
GO:0001810	regulation of type I hypersensitivity	P	3.55E-02	over
GO:0001805	positive regulation of type III hypersensitivity	P	3.55E-02	over
GO:0001803	regulation of type III hypersensitivity	P	3.55E-02	over
GO:0001802	type III hypersensitivity	P	3.55E-02	over
GO:0032765	positive regulation of mast cell cytokine production	P	3.55E-02	over
GO:0033026	negative regulation of mast cell apoptotic process	P	3.55E-02	over
GO:0033025	regulation of mast cell apoptotic process	P	3.55E-02	over
GO:0033024	mast cell apoptotic process	P	3.55E-02	over
GO:0033023	mast cell homeostasis	P	3.55E-02	over
GO:0016068	type I hypersensitivity	P	3.55E-02	over
GO:0034453	microtubule anchoring	P	3.71E-02	over
GO:0002705	positive regulation of leukocyte mediated immunity	P	3.71E-02	over
GO:0006575	cellular modified amino acid metabolic process	P	3.80E-02	over
GO:0045736	negative regulation of cyclin-dependent protein serine/threonine kinase activity	P	4.08E-02	over
GO:0033206	meiotic cytokinesis	P	4.08E-02	over
GO:0001556	oocyte maturation	P	4.08E-02	over
GO:0071168	protein localization to chromatin	P	4.08E-02	over
GO:0031648	protein destabilization	P	4.08E-02	over
GO:0007019	microtubule depolymerization	P	4.09E-02	over
GO:0009607	response to biotic stimulus	P	4.10E-02	over
GO:0007088	regulation of mitosis	P	4.74E-02	over
GO:0051783	regulation of nuclear division	P	4.74E-02	over
GO:0032989	cellular component morphogenesis	P	7.74E-03	under
GO:0044270	cellular nitrogen compound catabolic process	P	1.06E-02	under
GO:0034655	nucleobase-containing compound catabolic process	P	1.25E-02	under
GO:1901135	carbohydrate derivative metabolic process	P	1.25E-02	under
GO:1901657	glycosyl compound metabolic process	P	1.56E-02	under
GO:0009116	nucleoside metabolic process	P	2.04E-02	under
GO:0009259	ribonucleotide metabolic process	P	2.67E-02	under
GO:0019693	ribose phosphate metabolic process	P	2.67E-02	under
GO:0000902	cell morphogenesis	P	2.76E-02	under
GO:0046700	heterocycle catabolic process	P	2.93E-02	under
GO:0019439	aromatic compound catabolic process	P	2.93E-02	under
GO:0009150	purine ribonucleotide metabolic process	P	3.40E-02	under
GO:0009119	ribonucleoside metabolic process	P	3.40E-02	under
GO:0009653	anatomical structure morphogenesis	P	3.40E-02	under
GO:1901136	carbohydrate derivative catabolic process	P	3.80E-02	under
GO:0046128	purine ribonucleoside metabolic process	P	3.81E-02	under
GO:0042278	purine nucleoside metabolic process	P	3.81E-02	under
GO:1901361	organic cyclic compound catabolic process	P	4.58E-02	under

[1]GO categories: C (Cellular Component), F (Molecular Function), and P (Biological Process)
